# Palladium supported on a novel ordered mesoporous polypyrrole/carbon nanocomposite as a powerful heterogeneous catalyst for the aerobic oxidation of alcohols to carboxylic acids and ketones on water†

**DOI:** 10.1039/c9ra10941b

**Published:** 2020-04-03

**Authors:** Nasim Ganji, Babak Karimi, Sepideh Najafvand-Derikvandi, Hojatollah Vali

**Affiliations:** Department of Chemistry, Institute for Advanced Studies in Basic Sciences (IASBS) PO-Box 45195-1159, Gava-zang Zanjan 45137-6731 Iran karimi@iasbs.ac.ir bkarimi48@gmail.com; Research Center for Basic Sciences & Modern Technologies (RBST), Institute for Advanced Studies in Basic Sciences (IASBS) Zanjan 45137-66731 Iran; Department of Anatomy and Cell Biology and Facility for Electron Microscopy Research McGill University Montreal Quebec, H3A 2A7 Canada

## Abstract

Preparation of an ordered mesoporous polypyrrole/carbon (PPy/OMC) composite has been described through a two-step nanocasting process using KIT-6 as a template. Characterization of the PPy/OMC nanocomposite by various analysis methods such as TEM, XRD, TGA, SEM and N_2_ sorption confirmed the preparation of a material with ordered mesoporous structure, uniform pore size distribution, high surface area and high stability. This nanocomposite was then used for the immobilization of palladium nanoparticles. The nanoparticles were almost uniformly distributed on the support with a narrow particle size of 20–25 nm, confirmed by various analysis methods. Performance of the Pd@PPy/OMC catalyst was evaluated in the aerobic oxidation of various primary and secondary alcohols on water as a green solvent, giving the corresponding carboxylic acids and ketones in high yields and excellent selectivity. The catalyst could also be reused for at least 10 reaction runs without losing its catalytic activity and selectivity. High catalytic efficiency of the catalyst can be attributed to a strong synergism between the PPy/OMC and that of supported Pd nanoparticles.

## Abstract 2

Aerobic oxidation of alcohols to carboxylic acids and ketones in water using palladium species supported on an ordered mesoporous polypyrrole/carbon nanocomposite.

## Graphical abstract

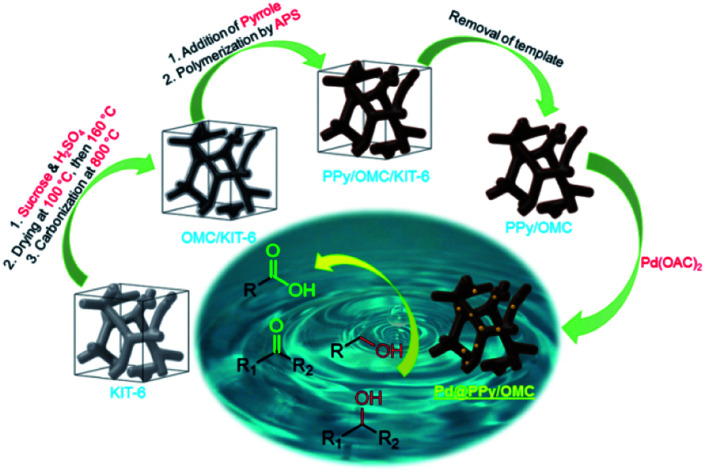

## Introduction

Oxidation of alcohols to carbonyl compounds is one of the most fundamental chemical reactions in organic synthesis both in academic and industrial areas,^1–5^ because the corresponding products have wide applications in synthetic chemistry as important precursors and intermediates. Among all the oxidized products, carboxylic acids are used in large scale for the preparation of valuable compounds such as pharmaceuticals, agrochemicals, polymers and commercial products.^6,7^ Global production of aryl carboxylic acids is estimated to be more than 620 kilotons by 2023.^8^ However, large scale synthesis of these carbonyl compounds still relies on the oxidation of alcohols with stoichiometric amounts of hazardous and expensive oxidants,^9,10^ such as permanganate, chromate and chlorite in halogenated solvents, resulting in the generation of the significant amounts of waste materials. Therefore, the development of mild, safe and environmentally friendly catalytic approaches based on the use of green reagents and solvents, and especially using molecular oxygen as a clean, atom economic and sustainable oxidizing reagent would provide noteworthy alternatives to the traditional oxidation methods.^11–21^ Additionally, a remarkable number of catalysts especially metal-based systems have successfully been developed for the aerobic oxidation of alcohols to corresponding carbonyl compounds, owing to the importance of the use of catalyst from the perspective of the principles of green chemistry.

One of the most significant metals for alcohol oxidation is palladium. Over the years, various homogeneous Pd catalysts, have extensively been examined in the aerobic oxidation of alcohols.^22–29^ It is worth noting that most of the efficient homogeneous catalysts in this transformation have containing nitrogen groups. Earlier mechanistic studies revealed that Pd(0) intermediates are stabilized through the coordination with the nitrogen-containing ligands, prior to their aerobic reoxidation to Pd(ii) particles. While, Pd catalysts without the ligands were extensively deactivated as a result of the Pd black formation.^30,31^ Moreover, scientists have focused on the development of efficient heterogeneous Pd catalysts for the aerobic oxidation of alcohols, because of disadvantages of homogeneous catalysts,^32–42^ including the necessity to high catalyst loadings and excess of ligands or bases as well as difficulties in removing and recycling expensive catalysts or ligands from the reaction mixture. Although, during the recent years a lot of progress has been achieved in the field of heterogeneous Pd catalysts. However, the majority of these reports have concentrated on the conversion of alcohols to aldehydes and ketones and only a few heterogeneous Pd catalysts have been presented for the oxidation of alcohols to carboxylic acids, for example, Pd/carbon,^37^ Pd–Bi–Te/activated carbon^39–41^ and Pd/DNA-MMT.^42^ Unfortunately, most of these heterogeneous Pd systems suffer from the necessity to promoters, low catalytic activity, limited substrate scope, high reaction temperature and/or wasteful organic solvents. In fact, the preparation of carboxylic acids through the direct oxidation of alcohols using heterogeneous Pd catalysts is still a live challenge. Therefore, the development of an ideal system based on a heterogeneous Pd catalyst along with the use of molecular oxygen as a green oxidant and also water as a green solvent would be very valuable.

Evidently, the nature of solid support is one of the most important factors in achieving highly efficient heterogeneous metal-based catalysts, because the support can play different roles in the catalytic process and also can influence the electron configuration of metal species.^43^ Among the wide range of solid supports, polymers and porous materials are of the most commonly reported solid supports.^44–47^ During the years, various polymers, especially polymers having nitrogen-based functional groups in their framework were applied for the immobilization of metal nanoparticles, taking the advantage of the high affinity of nitrogen functional groups to metal particles.^48^

Another extensively employed supports are porous materials, especially ordered mesoporous materials that owing to their attractive features including high surface areas, suitable and tunable pore sizes, narrow pore size distributions and accessible porous frameworks (which simplify the diffusion of reactant and product molecules) have been used as support in different chemical transformations.^49–52^ Mesoporous carbon materials are an interesting category of these porous materials that have been developed and applied in various fields because of their excellent chemical and thermal stability.^53–56^ However, from the view of application for the preparation of heterogeneous metal-based catalysts, introducing functional groups having high affinity for metal particles to the surface of porous carbons is necessary in most cases, because the interaction between the metal nanoparticles and the unmodified carbon surface is typically weak. This weak interaction can give rise to leaching and aggregation of deposited metal particles during the catalytic processes. In this connection, the modification of porous carbon materials has attracted the attention of researchers.^57,58^ One of the best functionalization approaches presented in this area is the polymer-based modification because resultant polymer-carbon composites could have the extraordinary and combined properties of the individual components and also benefit from the synergistic effects between them.^59,60^

Polypyrrole (PPy) is one of the most promising polymers for the preparation of polymer–carbon nanocomposites. This polymer has attracted more and more attention during the recent years because of its extraordinary properties such as the facile synthesis through chemical and electrochemical processes, excellent environmental stability, high conductivity, biocompatibility and low price.^61,62^ This polymer can functionalize the surface structure of carbon materials and also can lead to the higher affinity of materials for metal species by its high coordinating capability, derived from nitrogen atoms and π electrons of pyrrole units. In this regard, some research groups have recently focused on the development of mesoporous PPy–carbon composites. For example, Pinnavaia and co-workers reported the synthesis of a series of polypyrrole-ordered mesoporous carbon composites (PPy–OMCs) by incorporating PPy into a mesostructured carbon.^63^ In another report by Choi *et al.*, the surface of an ordered mesoporous carbon was selectively modified with the PPy.^64^ Moreover, in 2008 a strategy was presented for the preparation of a number of PPy–OMC composites by the introduction of a PPy layer into the pore structure of a mesoporous carbon through the vapor infiltration of pyrrole and its subsequent chemical polymerization.^65^ However, if PPy chains occupy the spaces of mesopores, the open and ordered pore structures that are highly desirable for catalytic applications will be destroyed to a great extent. Therefore, the preparation of PPy–carbon composites with controlled open mesostructure that also benefit from electronic and chemical properties of both of the involved materials, in an easy and cost-effective way is still a fundamental challenge.

**Scheme 1:**
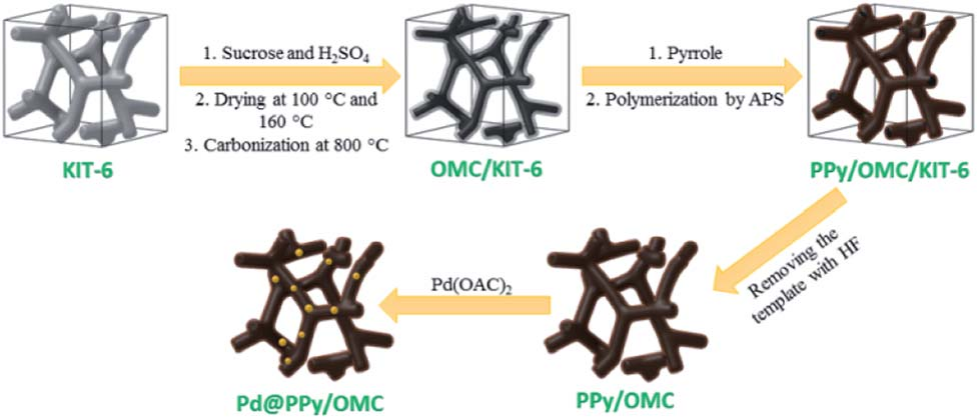
A schematic illustration of the synthetic procedure for the preparation of the Pd@PPy/OMC catalyst.

In this line, herein, we wish to represent a synthetic strategy for the preparation of an ordered mesoporous PPy–carbon composite, so that, its mesopore structure remains intact and is kept from being blocked up by PPy chains during the polymerization. In this approach, first the mesopores of an ordered mesoporous silica template (KIT-6) are partially filled with sucrose as a carbon precursor and then carbonized at 800 °C. In following, pores of the OMC/KIT-6 composite generated during the carbonization process are filled with a solution containing pyrrole monomers and then the monomers are polymerized using an oxidant. Finally, the silica template is removed using a solution of hydrofluoric acid (HF) to obtain the ordered mesoporous PPy/carbon nanocomposite, as outlined in Scheme 1. We selected PPy in this work because of its excellent electronic properties and this fact that pyrrole monomers can easily be adsorbed on carbon surfaces due to the molecular similarity between the pyrrole and carbon structure, as well as this point that PPy layers able to stick on solid surfaces strongly.^64^

Moreover, with the aim of profiting advantages of the mesoporous structure and also phenomenal features of the PPy/carbon composite as a support, palladium species are immobilized on this material and subsequently examined in the aerobic oxidation of alcohols in an aqueous medium. To the best of our knowledge, there is no report for the use of ordered mesoporous PPy/carbon composites in chemical transformations.

## Experimental

### Characterization

N_2_ sorption analysis was performed by a Belsorp analyzer (BELMAX, Japan) at 77 K. Samples were degassed at 373 K for 10 h before the measurements. Specific surface area of the materials was obtained from the relative pressure range 0.05–0.15 and their pore size distribution (PSD) was estimated by the use of the Barrett–Joyner–Halenda (BJH) method from the adsorption branch. Additionally, total pore volumes were assigned using the adsorbed volume at *P*/*P*_0_ ≈ 0.995. Pore structure of the samples was studied by Philips CM-200 and Titan Krios TEM instruments. Surface morphology of the materials was investigated using high resolution scanning electron microscopy (HRSEM) images taken by a TeScan-Mira III ultrahigh resolution cold field emission scanning electron microscope. X-ray powder diffraction (XRD) analysis was performed using a XPERT-PRO MPD diffractometer (Cu_Kα_ radiation) in the range of 0.8 to 10°. XPS spectra of the materials were recorded on a Kratos Analytical X-ray photoelectron spectrometer. Thermogravimetric analysis (TGA) was obtained using a NETZSCH STA 409 PC/PG instrument (Germany) at scan rates of 10 K min^−1^, with typically 5 mg sample from 25 to 800 °C under both of the N_2_ and O_2_ atmosphere. Nitrogen content of the materials was determined by elemental analysis (C, H, N) using the vario-EL CHNS instrument. Fourier transform infrared (FTIR) spectra of the materials were attained using a Bruker vector 22 instrument in the range of 400 and 4000 cm^−1^. Yield of oxidation reactions was determined using a Varian CP-3800 gas chromatograph instrument (GC) equipped with a capillary column and a flame-ionization detector (FID) using an internal standard method.

### Chemicals

Pluronic P123 (EO_20_PO_70_EO_20_, EO = ethylene oxide, PO = propylene oxide) was obtained from Aldrich. Pd(OAc)_2_ was purchased from Acros Organics. Tetraethyl orthosilicate (TEOS), hydrochloric acid (37%), sulfuric acid (98%) and also solvents were obtained from Merck Company and were utilized without further purification. Pyrrole was purchased from Aldrich and distilled under vacuum for more purification.

### Preparation of an ordered mesoporous silica template (KIT-6)

KIT-6, an ordered mesoporous silica template, was prepared according to the procedures reported in literature.^66,67^ In this connection, 5.5 g of P123 and 11.0 g of hydrochloric acid (37 wt%) were added to 201 g of deionized water and the resulting mixture was stirred for 6 h at 35 °C to obtain a homogeneous solution. Then, 5.5 g of *n*-butanol was poured into the solution and after 1 h, 11.44 g of TEOS was also added to it. Stir of the mixture was continued at 35 °C for 24 h. Next, the mixture was transferred into a teflon-lined autoclave and placed under static condition at 130 °C for 36 h. Content of the autoclave was filtered and washed with plenty of deionized water and ethanol. Finally, KIT-6 was obtained as a white solid by removing the P123 template through the calcination at 550 °C for 5 h under the air atmosphere.

### Synthesis of the OMC/KIT-6 composite

Inspired by the Hao's work,^68^ an OMC/KIT-6 composite was obtained as follows. The synthesized KIT-6 template was dried at 100 °C for 12 h and was also degassed under vacuum for 1 h to remove moisture. 1.25 g sucrose and 0.14 g H_2_SO_4_ (98 wt%) were added to 5 mL deionized water and the resulting solution was poured into a flask containing 1 gr degassed KIT-6 and the mixture was then sonicated for 45 min. The mixture was placed in an oven at 100 °C, and after 6 h, temperature of the oven increased to 160 °C and the material was heated in this temperature for 6 h. The brown solid was then carbonized at 800 °C for 1 h under the argon atmosphere to obtain the OMC/KIT-6 composite.

### Preparation of the PPy/OMC composite

Channels of the OMC/KIT-6 composite generated during the carbonization process could be used for deposition of PPy chains through the primary impregnation of Py monomers into the channels and then their *in situ* polymerization. For this purpose, the OMC/KIT-6 composite was degassed under vacuum for 30 min. The composite was then impregnated with an H_2_O : EtOH (1 : 1) solution containing an appropriate amount of Py monomers, calculated according to the pore volume of the OMC/KIT-6 composite. Flask of the reaction was placed into an ultrasonic bath for 45 min. The mixture was subsequently stirred for 24 h, with the aim of further penetration of Py monomers into the pores of the OMC/KIT-6 material. Extra solvents of the mixture were then removed under vacuum at 45 °C. Resulting solid was cooled in an ice bath and then, 200 mL HCl solution (1 mol L^−1^) containing ammonium persulfate (APS) as an oxidizing agent (molar ratio APS : Py 1.2 : 1) was added to it. The mixture was stirred at 0–5 °C for 24 h to complete the polymerization process. Finally, the black product was filtered and washed with plenty of deionized water and ethanol. Silica template of the as-synthesized PPy/OMC/KIT-6 composite was removed using a 10 wt% HF aqueous solution. Finally, the PPy/OMC composite was washed with deionized water and ethanol, and dried at 80 °C for 12 h.

In addition, an OMC material was obtained by removing the KIT-6 template from the initial OMC/KIT-6 composite to compare with the PPy/OMC composite.

### Preparation of the Pd@PPy/OMC catalyst

100 mg of the PPy/OMC composite was dispersed in 7 mL THF and sonicated for 45 min. Subsequently, 4.5 mg Pd(OAc)_2_ (0.02 mmol) dissolved in 3 mL THF was dropwisely added to the suspension and the mixture was stirred at room temperature for 12 h. Finally, the obtained Pd@PPy/OMC catalyst was filtered and washed with THF several times and was dried at 80 °C for 12 h.

### Oxidation of primary alcohols using the Pd@PPy/OMC catalyst

Primary alcohol (0.5 mmol), Pd@PPy/OMC (0.2 mol% of Pd), NaOH (30 mg, 0.75 equiv.) and H_2_O (0.5 mL) were added into a two-necked flask. A condenser containing a balloon filled with pure oxygen was placed on the reaction flask. After stirring at 80 °C for an appropriate time, the mixture was quenched with some drop of HCl and then filtered. The solution was extracted with H_2_O and ethyl acetate, and its organic layer was dried using anhydrous Na_2_SO_4_. Yield of the reaction was directly obtained by GC, without further purification of the product.

### Oxidation of secondary alcohols using the Pd@PPy/OMC catalyst

Secondary alcohol (0.5 mmol), Pd@PPy/OMC (1 mol%), NaOH (90 mg, 2.25 equiv.) and H_2_O (0.5 mL) were mixed in a two-necked flask. A condenser containing a balloon filled with pure oxygen was placed on the reaction flask. After stirring at 90 °C for an appropriate time, the reaction mixture was filtered and the solution was extracted with ethyl acetate and H_2_O. Finally, the organic layer containing the product was dried over anhydrous Na_2_SO_4_. Yield of the reaction was directly obtained by GC, without further purification of the product.

## Results and discussion

**Fig. 1:**
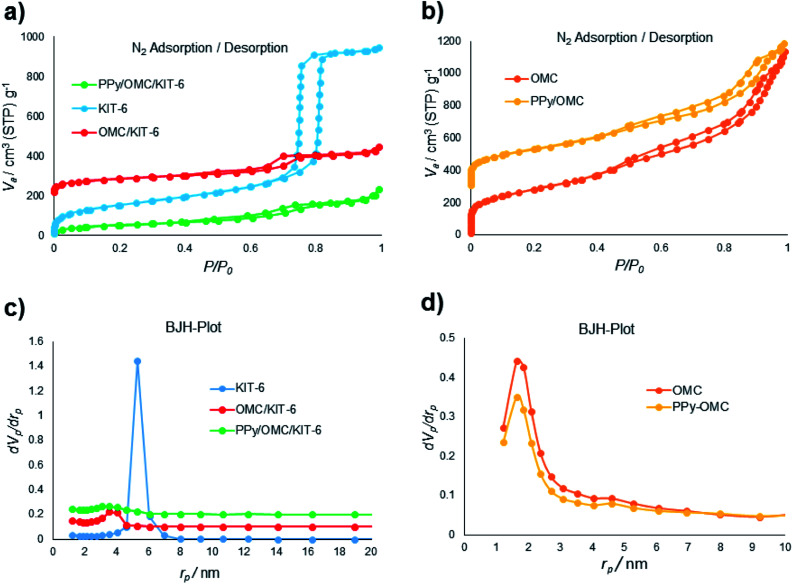
(a) and (b) N_2_ adsorption–desorption isotherms of the materials; (c) and (d) pore size distributions (PSDs) of the materials evaluated using the BJH method.

N_2_ sorption isotherms and pore size distributions (PSDs) of the materials (KIT-6, OMC/KIT-6, PPy/OMC/KIT-6, PPy/OMC and OMC (a mesoporous carbon prepared by the removal of KIT-6 from the OMC/KIT-6 composite for comparing its features with the PPy/OMC composite)) have been demonstrated in Fig. 1. All the isotherms display a hysteresis loop at the relative pressure of 0.4–0.8, showing a concentrated distribution of mesopores in these materials. Also, all of them possess characteristics of a type IV isotherm. Surface area and textural parameters of these materials have been summarized in Table 1. Surface areas were calculated according to the BET equation, and PSDs were evaluated through the BJH method using adsorption branches of the isotherms. Sharp capillary condensation in the isotherm of KIT-6 revealed the uniform distribution of mesopores. BET surface area and total pore volume of KIT-6 were 550 m^2^ g^−1^ and 1.46 cm^3^ g^−1^, respectively (Fig. 1a). As can be seen in the N_2_ isotherm and PSD of the OMC/KIT-6 composite, the one-time impregnation of sucrose into the pores of KIT-6 and its carbonization can create a space between KIT-6 and OMC, which is then available for the impregnation of pyrrole monomers. Decreasing both of the surface area and total pore volume from 288 m^2^ g^−1^ and 0.37 cm^3^ g^−1^ for the OMC/KIT-6 composite to 188 m^2^ g^−1^ and 0.32 cm^3^ g^−1^ for the PPy/OMC/KIT-6 composite confirmed the successful impregnation and polymerization of pyrrole monomers into the pores generated during the carbonization process (Fig. 1a and c). Comparing the isotherms and PSDs of the OMC and PPy/OMC samples demonstrated that the isotherm of the OMC has almost been maintained after the PPy addition, except for a slight decrease in the nitrogen uptake. Pore sizes of both of them were similar, so that their distribution curves showed a peak at 1.64 nm (Fig. 1b and d). Moreover, the acceptable distribution of mesopores for the PPy/OMC sample confirmed the open mesopore structure of the PPy/OMC composite and therefore the absence of pore blocking with PPy chains in the framework. This matter was ascribed to the presence of the temporarily silica template during the *in situ* polymerization of pyrrole inside the mesopores. BET surface areas and total pore volumes of the OMC and PPy/OMC samples were as follows: 1000 m^2^ g^−1^ and 1.75 cm^3^ g^−1^ for the OMC material and 822 m^2^ g^−1^ and 1.39 cm^3^ g^−1^ for the PPy/OMC composite, respectively (Table 1).

**Table 1:** Textural parameters of the materials

Sample	a *S*_BET_ (m^2^ g^−1^)	b *V*_t_ (m^3^ g^−1^)	c *D*_BJH_ (nm)
KIT-6	550	1.46	10.6
OMC/KIT-6	288	0.38	7.1
PPy/OMC/KIT-6	188	0.32	7.1
OMC	1000	1.75	3.3
PPy/OMC	822	1.39	3.3
Pd@PPy/OMC	628	1.18	3.3
Re–Pd@PPy/OMC	613	1.17	3.3

a *S*_BET_ = specific surface area calculated from the linear part of the BET plot, (*P*/*P*_0_ ≈ 0.05–0.15).

b *V*_t_ = total pore volume determined according to N_2_ adsorbed at *P*/*P*_0_ ≈ 0.995.

c *D*_BJH_ = average pore diameter calculated using BJH method.

**Fig. 2:**
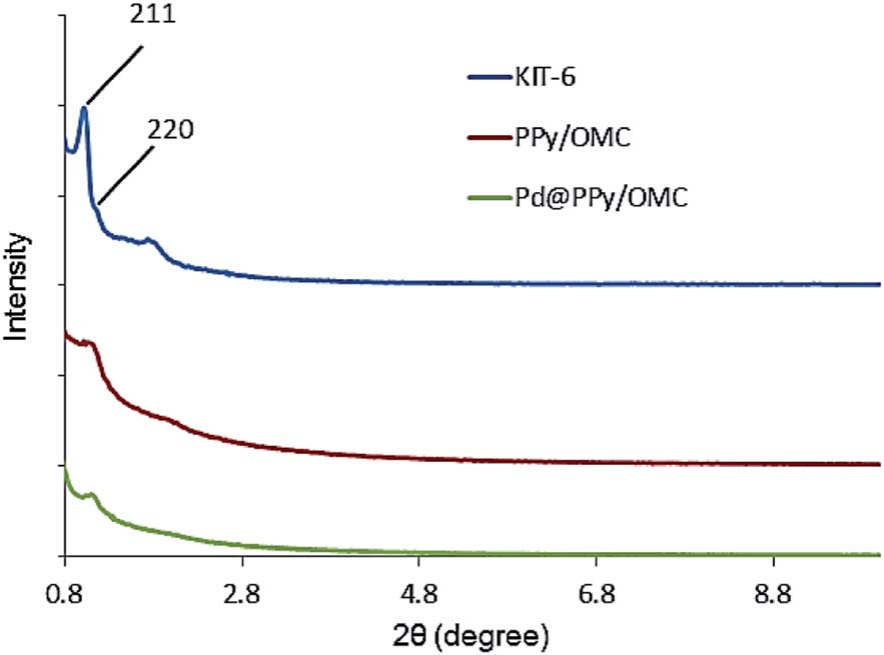
Low-angle X-ray diffraction patterns of KIT-6, PPy/OMC and Pd@PPy/OMC.

Low-angle X-ray diffraction (XRD) was used to confirm the ordered mesoporous structure of the synthesized composite (Fig. 2). Well-resolved diffraction peaks observed for the KIT-6 template were characteristics of a highly ordered bicontinuous cubic *Ia*3*d* symmetry structure.^69^ The PPy/OMC displayed the XRD pattern of a cubic structure, which was similar to the KIT-6 pattern,^70^ confirming that mesostructure of the composite is an inverse replica of the KIT-6 pore structure. However, the PPy/OMC replica revealed some decrease in the intensity of its peaks as compared with the KIT-6, illustrating a partial loss of the long-range order of structure during the nanocasting process.^71^ In addition, the slight shift of the low-angle XRD peak of 211 toward higher angles for the PPy/OMC composite may be related to the contraction of the structure after replication; the phenomenon that has been reported in some previous articles.^70,72^

**Fig. 3:**
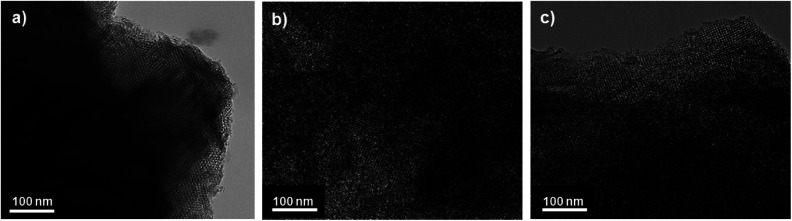
TEM images of (a) KIT-6, (b) OMC and (c) PPy/OMC.

Pore structures of the KIT-6, OMC and PPy/OMC materials were also investigated by using the HRTEM, as shown in Fig. 3. The well-ordered arrangement of pores and the uniform size of channels in the HRTEM image of KIT-6 confirmed that the template has been well synthesized (Fig. 3a). Additionally, the sufficiency of the one-time impregnation method for preparing a mesoporous carbon with stable ordered mesostructure was verified by checking the HRTEM image of the OMC sample (Fig. 3b). Moreover, the comparison between the HRTEM images of the OMC and PPy/OMC materials revealed that the order of the OMC mesostructure was not destroyed by the addition of PPy, the result which is in good agreement with the XRD and N_2_ sorption data (Fig. 3c).

**Fig. 4:**
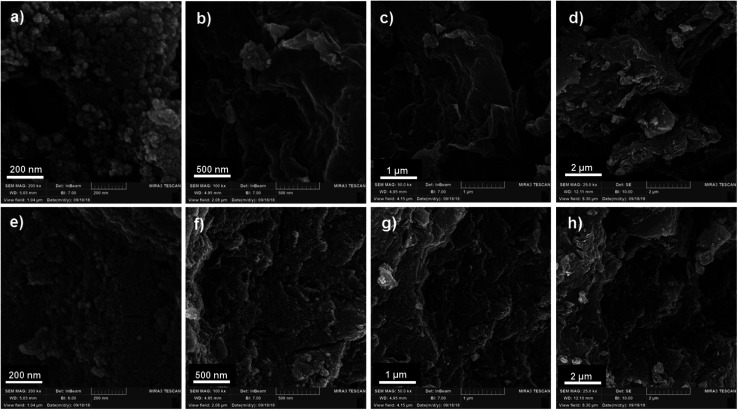
Typical FESEM images of OMC (a: 200 nm, b: 500 nm, c: 1 μm and d: 2 μm) and PPy/OMC (e: 200 nm, f: 500 nm, g: 1 μm and h: 2 μm).

Field emission scanning electron microscopy (FESEM) was used to investigate the morphology and surface state of the OMC and PPy/OMC materials (Fig. 4). SEM Images of the OMC and PPy/OMC samples, both indicate a cauliflower-like structure consisted of agglomerated particles (Fig. 4a and e), and a nanosheet-like morphology (Fig. 4b–d and f–h). This similarity proves the structural stability of the material during the polymerization process and removal of the template. However, the presence of PPy on the surface of the OMC has made the surface rougher than that of the OMC, as shown in Fig. 4c and g.

**Fig. 5:**
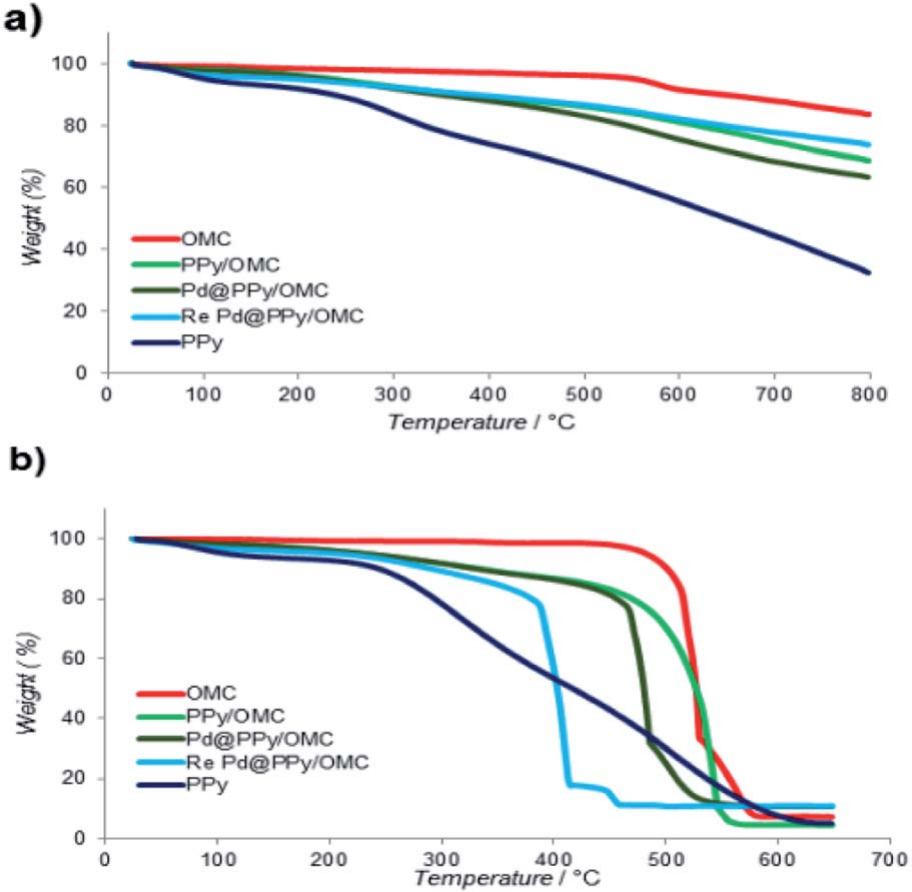
Thermogravimetric analysis curves under (a) nitrogen and (b) oxygen atmospheres.

Thermogravimetric analysis (TGA) was used in order to evaluate the thermal stability of the composite and also the loading amount of PPy on the OMC. This analysis was performed in both of the nitrogen and oxygen atmosphere, in the temperature range of 25–800 °C with heating rate of 10 °C min^−1^ (Fig. 5). Weight loss appeared in all samples at *T* < 150 °C was related to the removal of water and other volatile compounds from the materials. Broad weight loss observed for the PPy/OMC and PPy samples in the whole temperature range could be related to the wide molecular weight distribution of the PPy chains (Fig. 5a). However, the PPy/OMC nanocomposite showed a much better thermal stability in comparison with PPy, as it is clearly observable from Fig. 5a. It should be noted that when the analysis is carried out in the inert atmosphere, PPy chains convert to a nitrogen-containing carbon material. Due to the stability of the OMC at *T* < 480 °C under the O_2_ atmosphere, the weight loss in the PPy/OMC curve at the temperature range of 150–480 °C can be attributed to the thermal decomposition of PPy chains. Therefore, the weight loss at this temperature range was used for estimation the loading amount of the polymer on the OMC that was approximated to be around 14.6% (Fig. 5b). Chemical composition of the PPy/OMC nanocomposite was also determined by the elemental analysis that showed the weight percentage of 0.62% for nitrogen.

**Fig. 6:**
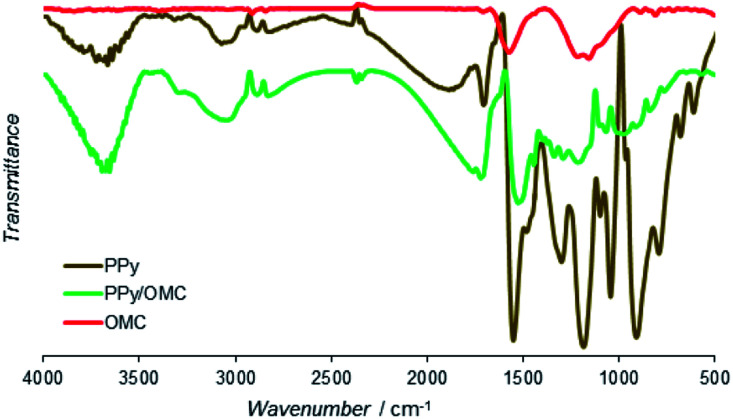
Infrared spectra of PPy, OMC and PPy/OMC powders.

Functional groups of the PPy, PPy/OMC and OMC materials were characterized by the Fourier-transform infrared spectroscopy (Fig. 6). Spectrum of PPy showed main bands similar to the PPy samples reported earlier.^73^ Bands at 1553 and 1474 cm^−1^ were related to C=C and C–N stretching vibration modes, respectively. In addition, the band at 1185 cm^−1^ was assigned to the C–C breathing vibration mode. Moreover, the bands at 1303, 1095 and 1042 cm^−1^ were attributed to the =C–H in-plane vibration modes of the pyrrole ring and the bands at 790 and 913 cm^−1^ were corresponding to the =C–H out-of-plane vibration modes.^74^ There were not many visible peaks in the FTIR spectrum of the OMC because of its high carbon content and strong C–C bonds.^75^ However, the small peaks at 2921 and 2852 cm^−1^ and the peaks at 1715, 1571 and 1213 cm^−1^ could be related to the stretching vibrations of C–H, C=O, C=C, and C–O bonds, respectively. Spectrum of PPy/OMC is similar to the spectrum of PPy, only with lower intensities of the peaks, as can be seen in Fig. 6. Characteristic vibration bands at 1521, 1444, 1067 and 805 cm^−1^ verified the presence of PPy in the composite very well. The slight shift observed in the position of some peaks may be related to interactions between the OMC and PPy layers.

**Fig. 7:**
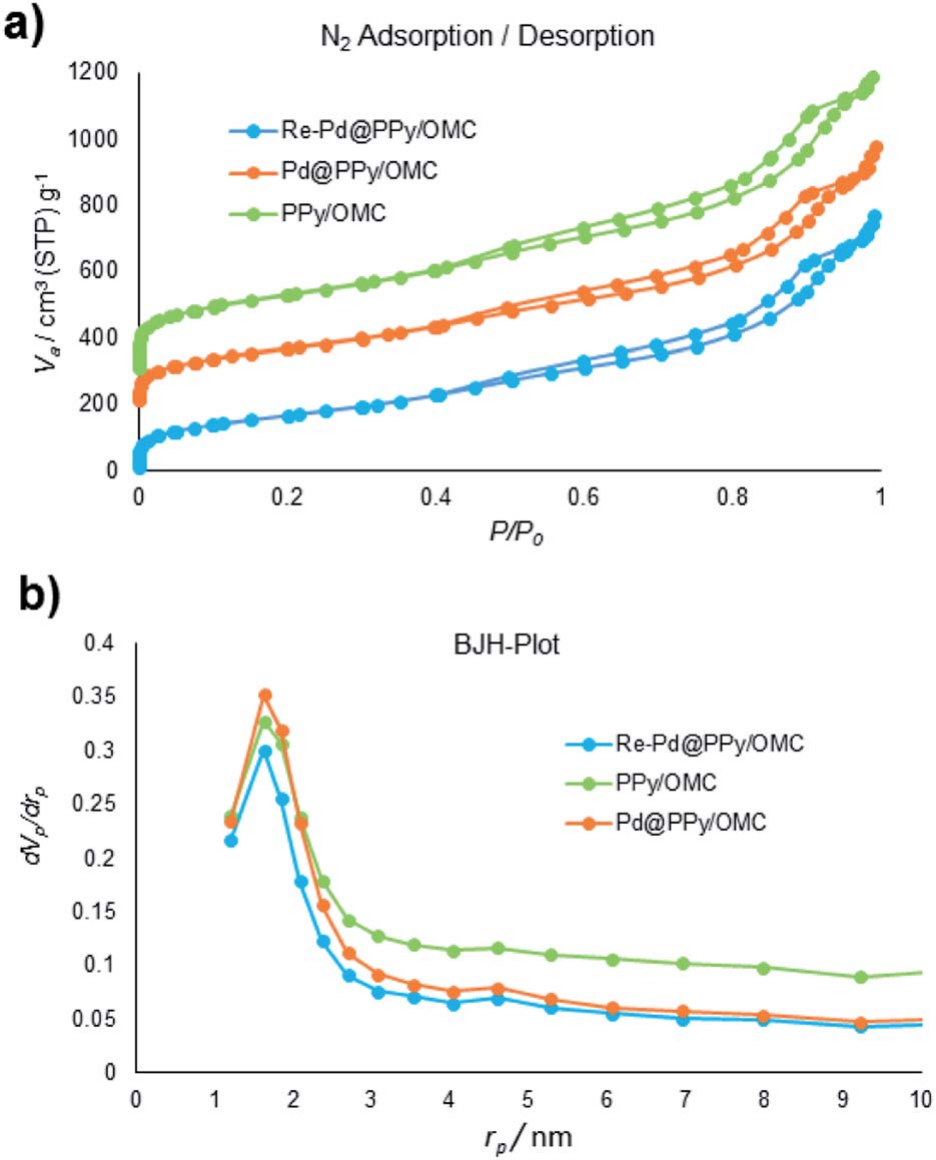
(a) N_2_ adsorption isotherms of the samples, and (b) pore size distributions (PSDs) of samples evaluated by the BJH method.

Next, we turned our attention into the investigation of the performance of the PPy/OMC composite as support for immobilizing palladium species and the application of the resulting catalyst in organic transformations. For this purpose, the Pd@PPy/OMC catalyst was prepared by the slow addition of an appropriate concentration of Pd(OAc)_2_ to a uniform mixture of the PPy/OMC composite dispersed in THF. A catalyst with well-dispersed Pd particles on the surface was expected to be achieved due to the presence of nitrogen in the support structure. This material was thoroughly characterized by the XPS, TEM, XRD, AAS and N_2_ sorption analyses. Nitrogen adsorption–desorption isotherm of the Pd@PPy/OMC catalyst provided a strong evidence for the successful immobilization of the Pd species on the PPy/OMC material, by showing an appreciable decrease in the BET surface area and total pore volume from 822 m^2^ g^−1^ and 1.39 cm^3^ g^−1^ for the PPy/OMC composite to 628 m^2^ g^−1^ and 1.18 cm^3^ g^−1^ for the Pd@PPy/OMC catalyst, respectively (Table 1). N_2_ sorption analysis also demonstrated that the open mesostructure and the structural order of the parent composite can be preserved during the deposition of Pd species (Fig. 7). Therefore, it is expected that enough spaces would still be existed in the catalyst nanospaces for the diffusion of substrates.

**Fig. 8:**
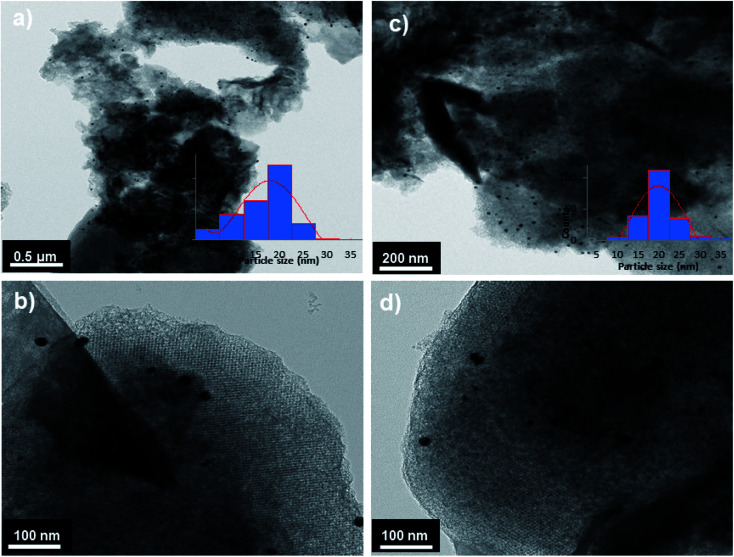
TEM images of Pd@PPy/OMC (a and b) and Re–Pd@PPy/OMC (c and d) samples including the particle-size distribution (insets).

Pd@PPy/OMC catalyst also showed a low angle XRD pattern similar to the pattern observed for the PPy/OMC support, confirming the preservation of the ordered cubic structure of the composite during deposition of the palladium species (Fig. 2). However, the relatively weaker intensity of the peaks for the supported palladium catalyst compared to the mesoporous composite can be attributed to the penetration of the palladium species into the pores of the PPy/OMC composite. Moreover, the TEM technique revealed the presence of Pd nanoparticles, and at the same time maintaining the order of the mesostructure (Fig. 8a and b). TEM images of the catalyst and the pattern of particle-size distribution, shown in the inset of Fig. 8a, demonstrated that the Pd species are almost uniformly distributed on the PPy/OMC material, with a narrow range of 20–25 nm. Pd content in the catalyst was quantitatively determined using the atomic absorption spectroscopy (AAS) of the acid-washed Pd@PPy/OMC sample and showed the loading amount of 0.2 mmol g^−1^.

According to the TGA data, the Pd@PPy/OMC catalyst demonstrated thermal stability close to thermal stability of the PPy/OMC composite. This matter confirmed the stability of the composite after deposition of Pd particles (Fig. 5). However, a slightly higher thermal stability observed for the PPy/OMC composite compared to the Pd@PPy/OMC catalyst under the O_2_ atmosphere (the PPy/OMC was decomposed at 480 °C, whereas the Pd@PPy/OMC was decomposed at 465 °C) was attributed to the fact that the presence of metal nanoparticles can effectively catalyze decomposition of materials.^76,77^

**Fig. 9:**
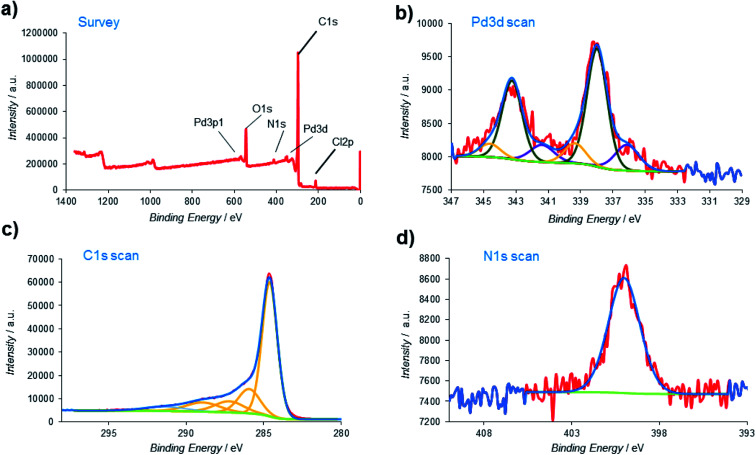
(a) XPS survey spectrum of the Pd@PPy/OMC; high-resolution XPS spectrum of (b) palladium; (c) carbon and (d) nitrogen.

X-ray photoelectron spectroscopy (XPS), one of the most effective analysis methods for the identification of various species on heterogeneous surfaces, was also employed to obtain additional insights about the nature of the Pd@PPy/OMC material at atomic scale (Fig. 9). The signals located at around 200, 284, 340, 400, and 532 eV in the XPS survey spectrum were assigned to Cl, C, Pd, N and O, respectively (Fig. 9a). High resolution Pd 3d XPS spectrum displayed three groups of signals. The first pair of the peaks at lower binding energies 336.13 and 341.39 eV were attributed to the Pd 3d_5/2_ and Pd 3d_3/2_ of Pd(0). The second pair of Pd signals, *i.e.* the 337.99 and 343.25 eV peaks were assigned to the Pd 3d_5/2_ and Pd 3d_3/2_ of Pd(ii) with lower charge density.^78,79^ Surprisingly, another pair of signals can be seen at higher binding energies (the 339.36 and 344.62 eV peaks). These peaks might be related to the Pd particles that directly interact with existing electron deficient groups on the surface of the mesoporous carbon (Fig. 9b).^80^ However, the Pd 3d XPS spectrum revealed that dominant component is Pd(ii) (69.14%). As shown in Fig. 9c, the C 1s peak was deconvoluted into five peaks. The main peak at 284.62 eV was originated from the C–C/C=C carbon groups, while the other weak peaks at 285.95, 287.22, 288.93 and 291.37 eV were attributed to the carbon atoms bound to the oxygen and nitrogen atoms in the composite (C–N, C–O–C, C=O and –COOH groups).^81,82^ Moreover, the binding energy at 400.1 eV in the N 1s spectrum was corresponding to the nitrogen atom in the PPy part (Fig. 9d).^83^ The presence of the Cl 2p peak in the survey spectrum might be related to the existence of Cl^−^ dopants.

After the successful preparation and characterization of the Pd@PPy/OMC catalyst, the performance of this material was evaluated in the aerobic oxidation of alcohols in the presence of molecular oxygen, with highlighting the possibility of using water as an ideal green solvent. In order to optimize reaction conditions, the effect of diverse variables such as catalyst amount, type and quantity of base and amount of water as well as the reaction temperature was examined on the aerobic oxidation of benzyl alcohol as a test substrate, as shown in Table 2. We started our investigations with the oxidation reaction of benzyl alcohol (1 mmol) with the Pd@PPy/OMC catalyst (0.4 mol% [Pd]) on 4 mL H_2_O at 80 °C temperature. Under these conditions, no progress was observed even after stirring for 12 h (Table 2, entries 1 and 2). Therefore, we studied the influence of adding different bases such as K_2_CO_3_, Na_2_CO_3_ and NaOH to the reaction vessel. NaOH (1.5 equiv.) showed the best result among these various bases by giving 73% benzoic acid as the main product (Table 2, entries 3–5). It should be noted that except NaOH, the other bases led to very poor yields and selectivity under these conditions. Another important point is the formation of the carboxylic acid in almost all of the examined conditions, most likely resulted from the high catalytic activity of the catalyst. This high catalytic activity may be attributed to the binding of Pd species to strong withdrawing groups onto the surface of the PPy/OMC composite. Remarkably, we found that decreasing the amount of water to less than 1 mL results in significantly higher yields and can improve the repeatability of the reaction (Table 2, entries 5–7). This diminution is also noteworthy from the environmental point of view. In the next step, we investigated the impact of different amounts of the catalyst on the reaction process, as shown in Table 2, entries 7–9. Yields and selectivity of the oxidation reaction did not changed by reducing the amount of the catalyst from 0.4 to 0.2 mol% of [Pd] (Table 1, entries 7 and 8). However, further decreasing of the amount of catalyst (0.1 mol% [Pd]) resulted in a lower yields (entry 9). Screening the quantity of the base and reaction temperature showed that their decrease can result in a noticeable reduction in the yields (Table 2, entries 10–13). In addition, the reaction time was optimized and two hours was chosen as the optimum time of the reaction by giving the quantitative yield of benzoic acid (Table 2, entries 14–16). After these screening experiments, the reaction condition in accordance with entry 15 was selected as the optimal reaction conditions. We also showed that the bare PPy/OMC support do not show any activity for the aerobic oxidation of benzyl alcohol under the optimal experimental conditions (Table 2, entry 17). It is worth mentioning higher increasing in the catalyst amount the to 0.6 mol%, did not beneficial in improving the catalyst performances (Table 2, entry 18). To further prove the important role of PPy/OMC support of our catalytic system, we also performed an oxidation reactions in the presence of a homogeneous Pd catalyst (Pd(OAc)_2_). Interestingly, the reaction using Pd(OAc)_2_ (0.6 mol% [Pd]) showed a weak performance in the oxidation of benzyl alcohol, most probably because of the Pd black formation (Table 2, entries 19 and 20).

**Table 2:** Optimization of the aerobic oxidation reaction of benzyl alcohol using the Pd@PPy/OMC catalyst

Entry	Catalyst (mol% Pd)^b^	Base (equiv.)	Solvent (mL)	*T* (°C)	Time (h)	Yield of benzaldehyde^a^ (%)	Yield of benzoic acid^a^ (%)	TON^c^
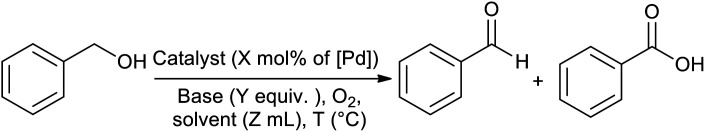								
1	Pd@PPy/OMC (0.4)	—	H_2_O (4)	80	2	0	0	—
2	Pd@PPy/OMC (0.4)	—	H_2_O (4)	80	12	2	0	—
3	Pd@PPy/OMC (0.4)	Na_2_CO_3_ (2)	H_2_O (4)	80	12	30	21	105
4	Pd@PPy/OMC (0.4)	K_2_CO_3_ (2)	H_2_O (4)	80	12	25	35	175
5	Pd@PPy/OMC (0.4)	NaOH (1.5)	H_2_O (4)	80	12	18	73	365
6	Pd@PPy/OMC (0.4)	NaOH (1.5)	H_2_O (2)	80	12	10	90	450
7	Pd@PPy/OMC (0.4)	NaOH (1.5)	H_2_O (1)	80	12	0	100	499
8	Pd@PPy/OMC (0.2)	NaOH (1.5)	H_2_O (1)	80	12	0	100	1000
9	Pd@PPy/OMC (0.1)	NaOH (1.5)	H_2_O (1)	80	12	31	54	1080
10	Pd@PPy/OMC (0.2)	NaOH (2)	H_2_O (1)	80	12	1	99	990
11	Pd@PPy/OMC (0.2)	NaOH (1)	H_2_O (1)	80	12	35	48	480
12	Pd@PPy/OMC (0.2)	NaOH (1.5)	H_2_O (1)	70	12	39	14	139
13	Pd@PPy/OMC (0.2)	NaOH (1.5)	H_2_O (1)	90	12	0	100	1000
14	Pd@PPy/OMC (0.2)	NaOH (1.5)	H_2_O (1)	80	6	0	100	1000
**15**	**Pd@PPy/OMC (0.2)**	**NaOH (1.5)**	**H**_**2**_ **O (1)**	**80**	**2**	**1**	**99**	**990**
16	Pd@PPy/OMC (0.2)	NaOH (1.5)	H_2_O (1)	80	1	15	73	730
17	PPy/OMC	NaOH (1.5)	H_2_O (1)	80	2	8	0	—
18	Pd@PPy/OMC (0.6)	NaOH (1.5)	H_2_O (1)	80	1.5	1	99	330
19	Pd(OAc)_2_ (0.6)	NaOH (1.5)	H_2_O (1)	80	2	18	1	—
20	Pd(OAc)_2_(0.6)	NaOH (1.5)	H_2_O (1)	80	12	32	15	50
21^d^	Pd@PPy/OMC (0.2)	NaOH (1.5)	H_2_O (1)	80	2	57	33	330
22^e^	Pd@PPy/OMC (0.2)	NaOH (1.5)	H_2_O (1)	80	2	27	67	670

a GC yield using internal standard method.

b The catalyst was prepared by the impregnation of 0.02 mmol Pd(ii) in 100 mg PPy/OMC nanocomposite.

c
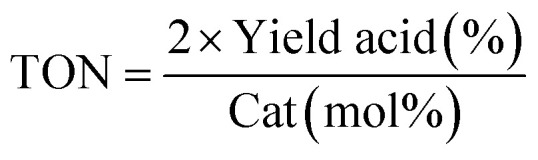
, although, due to some limitations, the reactivity differences have been compared in all data at the high end conversions, a more realistic comparison could be achieved at lower initial conversion.

d The catalyst was prepared by the impregnation of 0.04 mmol Pd(ii) in 100 mg PPy/OMC nanocomposite.

e The catalyst was prepared by the impregnation of 0.06 mmol Pd(ii) in 100 mg PPy/OMC nanocomposite.

Finally, we also investigated a series of Pd@PPy/OMC catalysts having different initial loadings of Pd in the oxidation reaction under the reaction condition similar to entry 15 (Table 2, entries 21 and 22). As can be seen in Table 2, entry 23, by increasing the initial loading of Pd to 0.6 mmol g^−1^ the yields of reaction dropped remarkably. Hence, 0.2 mmol g^−1^ was selected as the best loading for preparation of catalyst.

With the optimized reaction conditions in hand, we studied the possibility of using the Pd@PPy/OMC catalyst for the aerobic oxidation of diverse types of primary and secondary alcohols to their corresponding carbonyl compounds by testing a wide range of substrates (Table 3). As shown in Table 3, diverse types of primary benzylic alcohols, both electron rich and electron deficient derivatives, displayed an admirable reactivity and afforded the corresponding carboxylic acids in excellent yields and selectivities (>90%) (Table 3, entries 1–16). It is noteworthy that this catalyst system also showed excellent performance in the oxidation of 4-(methylthio)benzyl alcohol that is a highly challenging substrate in metal-catalyzed aerobic oxidation systems, because this substrate typically deactivates metal catalysts by its strong coordination to metal centers (Table 3, entry 5).^84^ Another remarkable feature of this protocol was its capability to oxidize benzylic alcohols bearing sterically demanding substituents such as 2-methyl-, 2-chloro- and 2-metoxy groups, although some slight modifications were required to achieve high efficiency and excellent yields (Table 3, entries 13–16). Cinnamyl alcohol as a complex substrate that can undergo several side reactions, because of its active C=C bond, was mainly oxidized to benzoic acid (Table 3, entry 17). The catalytic system also demonstrated outstanding activity in the oxidation of less reactive primary aliphatic alcohols to the corresponding carboxylic acids in good to excellent yields, though it was necessary to increase the temperature and amount of the catalyst to ensure their complete conversions (Table 3, entries 18–21). To the best of our knowledge, such efficient aerobic oxidation of different primary alcohols to carboxylic acids, including challenging substrates, using a heterogeneous Pd catalyst possessing a low loading of metal and under such green reaction conditions has not been reported to date. Despite the low reactivity of secondary alcohols, we discovered that the presented system was also applicable for the oxidation of secondary benzylic and cyclic aliphatic alcohols on pure water, giving the ketones in excellent yields, just by some modifications in the amount of catalyst and NaOH (Table 2, entries 22–26). Unfortunately, 2-adamantanol and open-chain secondary alcohols proved to be very sluggish substrates, obtaining the corresponding ketones in poor yields, even when the catalyst with 2 mol% [Pd] was employed (Table 3, entries 27–29).

**Table 3:** Substrate scope for the aerobic alcohol oxidation with the Pd@PPy/OMC catalyst

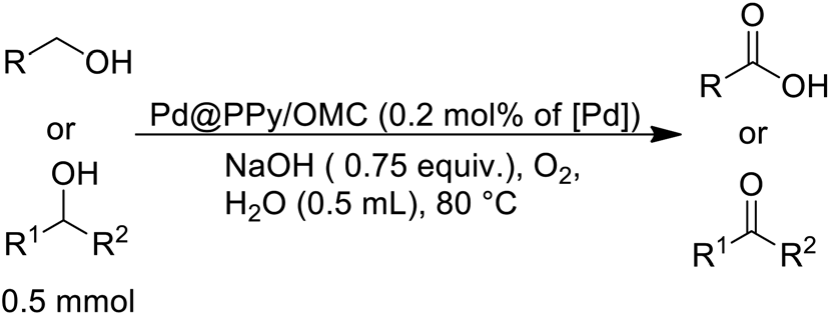					
Entry	Substrate	Product	Time (h)	Yield (%)	TON
1	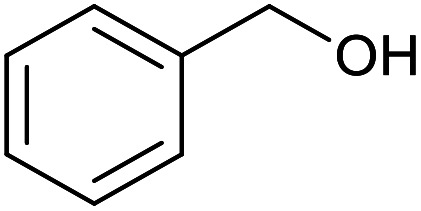	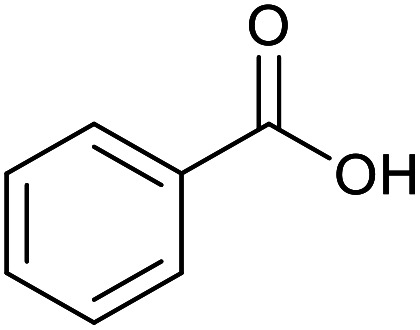	2	99	990
2	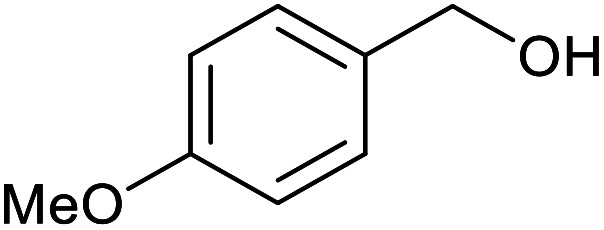	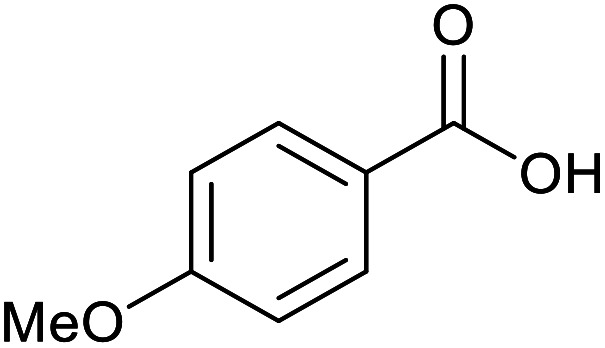	8	98	980
3	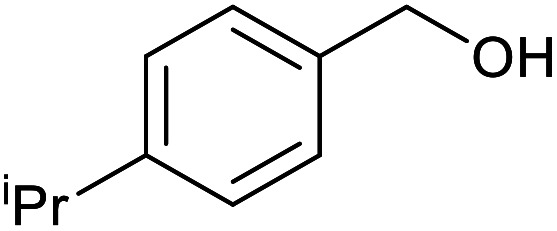	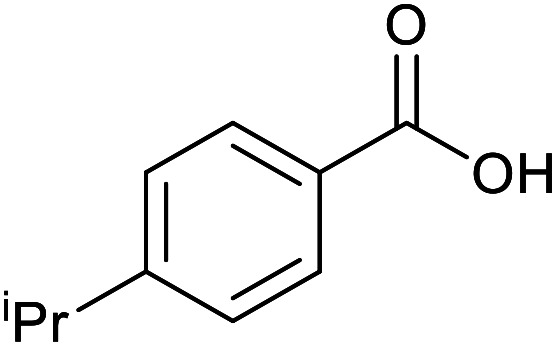	10	92	920
4	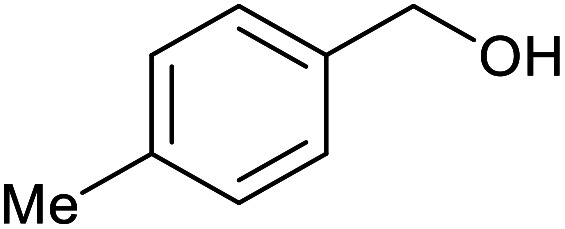	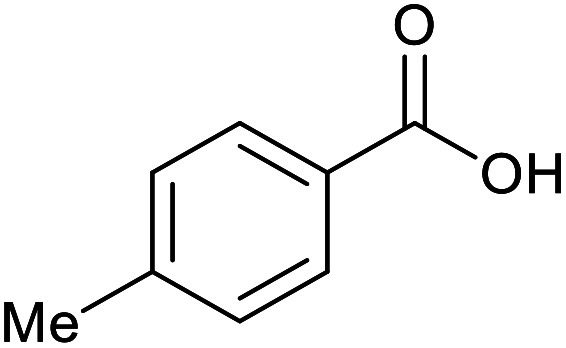	8	97	970
5	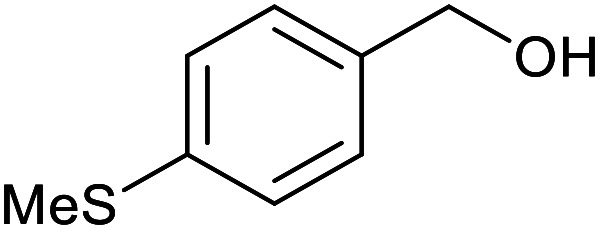	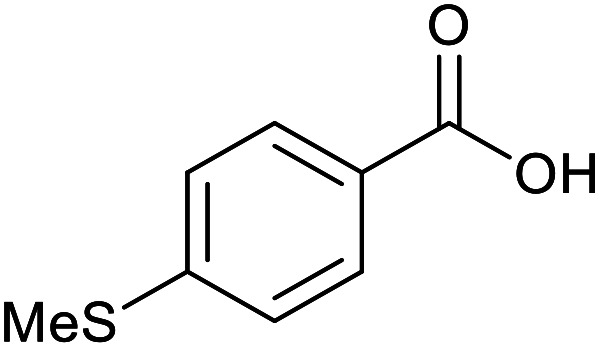	14	83	830
6	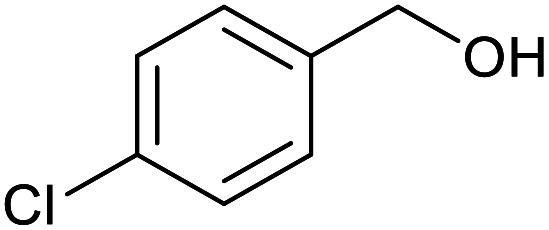	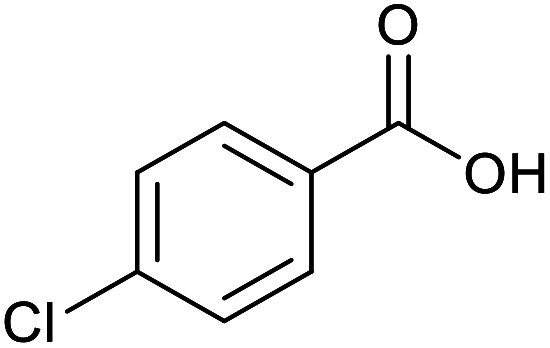	12	94	940
7	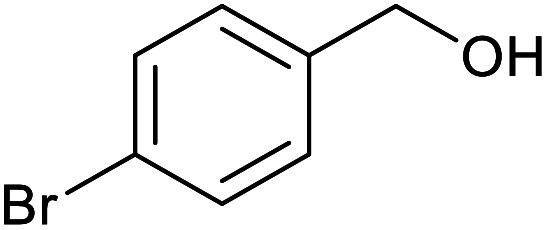	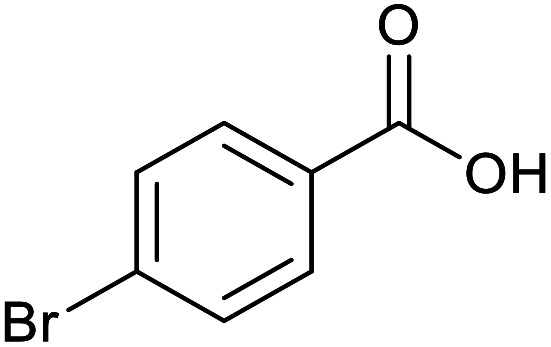	13	95	950
8^a^	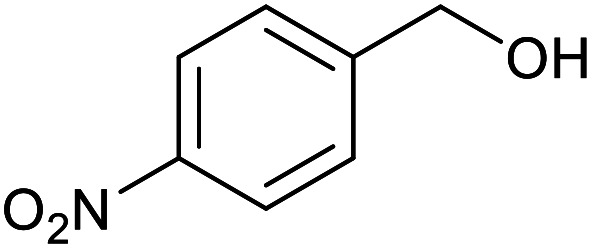	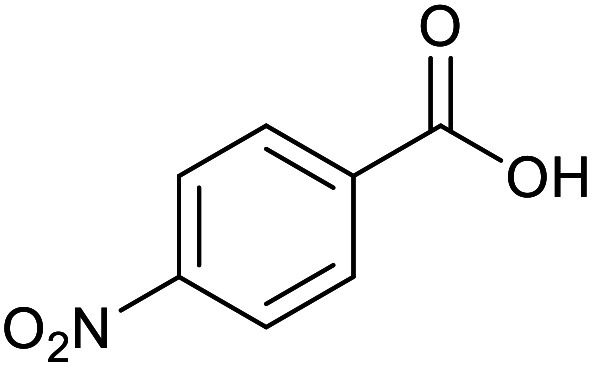	7	98	980
9	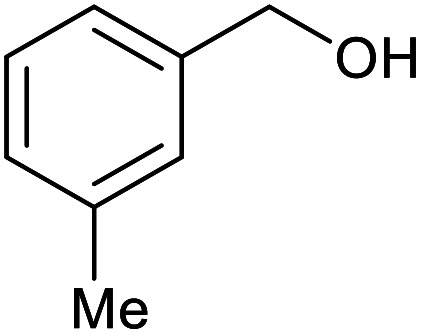	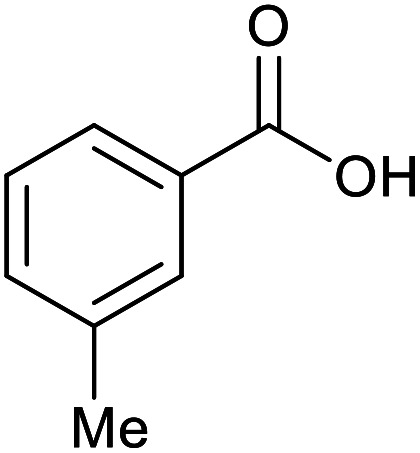	10	97	970
10	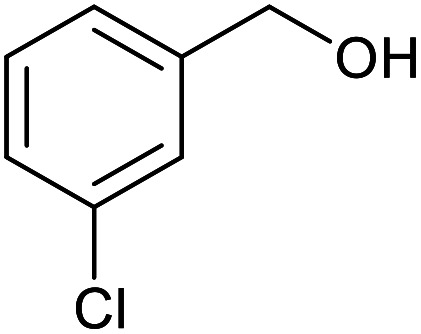	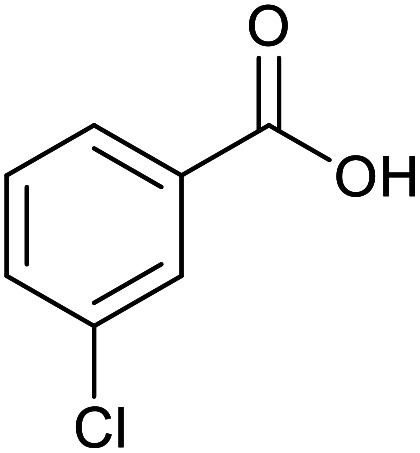	15	90	900
11	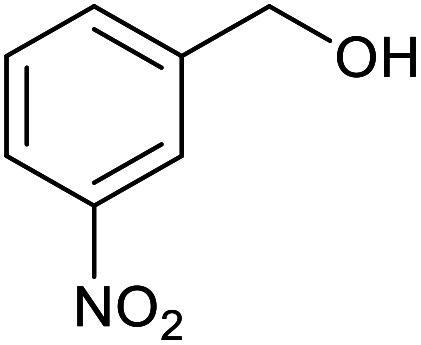	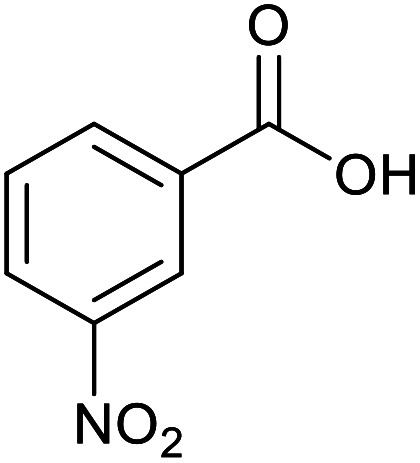	15	83	830
12	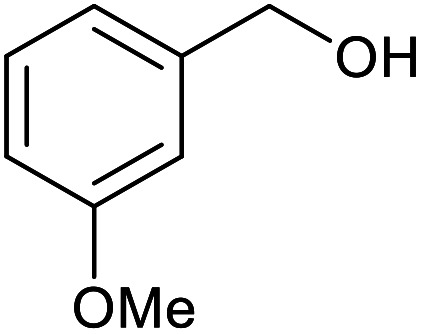	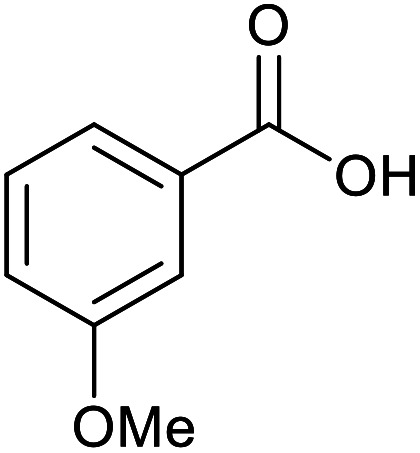	8	96	960
13^b^	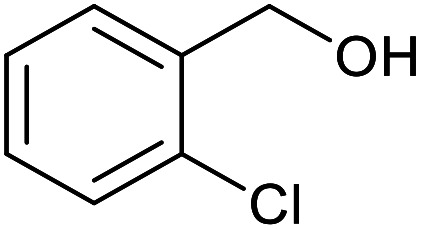	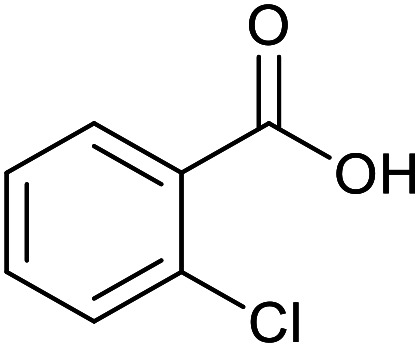	15	95	475
14^b^	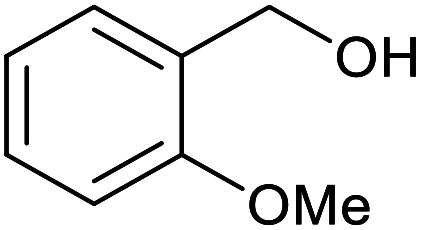	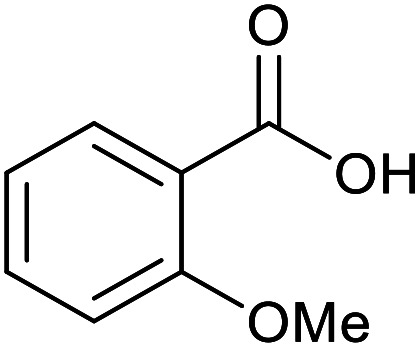	14	96	480
15^b^	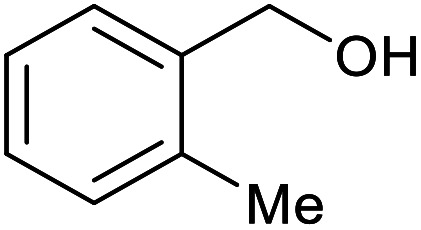	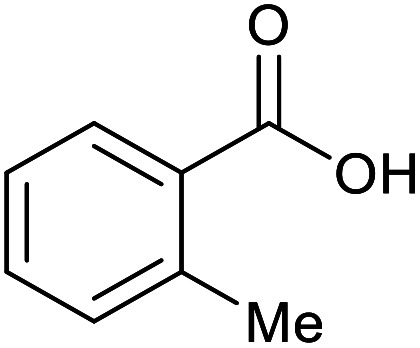	18	93	465
16^b^	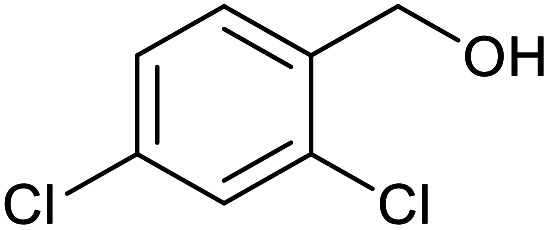	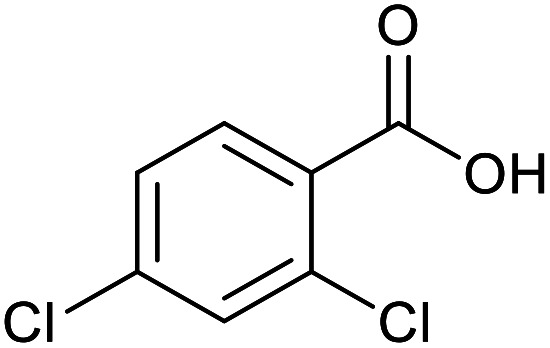	16	87	435
17	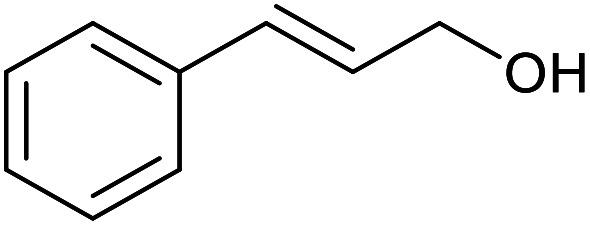	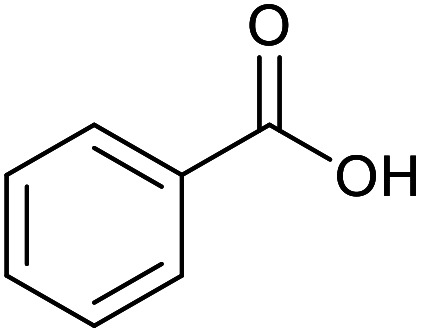	24	86	860
18^c^	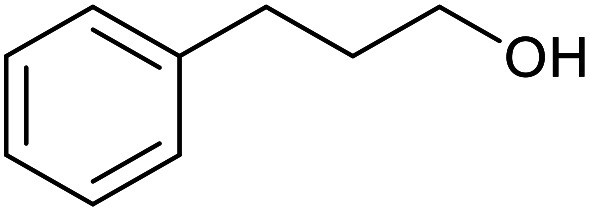	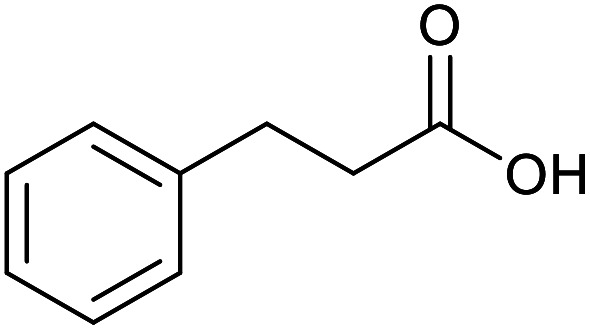	13	94	188
19^c^	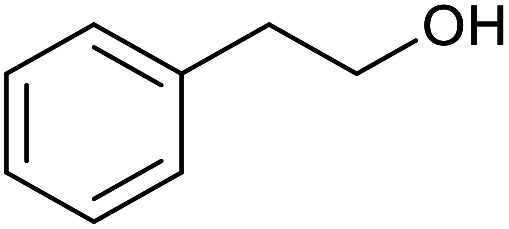	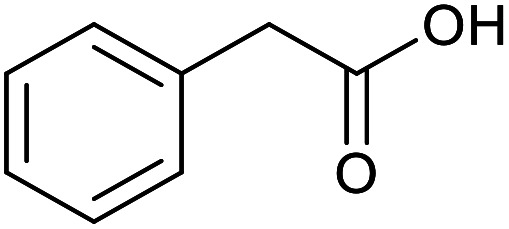	18	100	200
20^c^	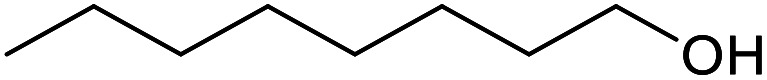	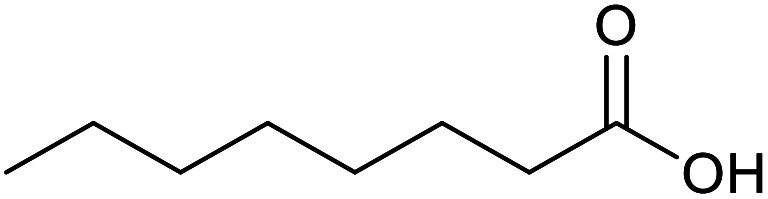	30	100	200
21^c^	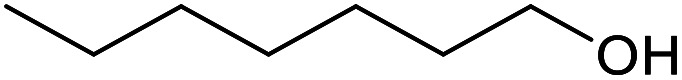	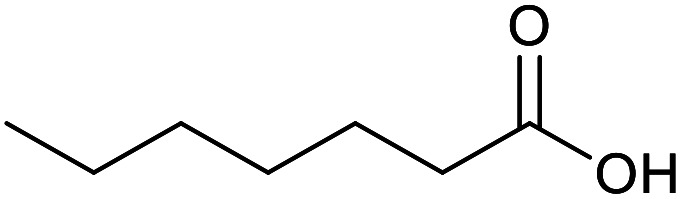	21	65	130
22^d^	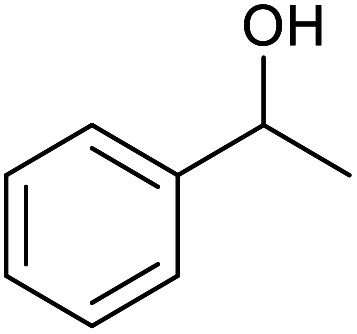	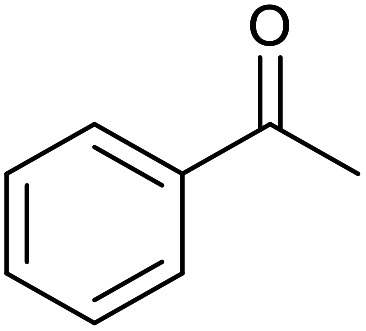	20	99	99
23^d^	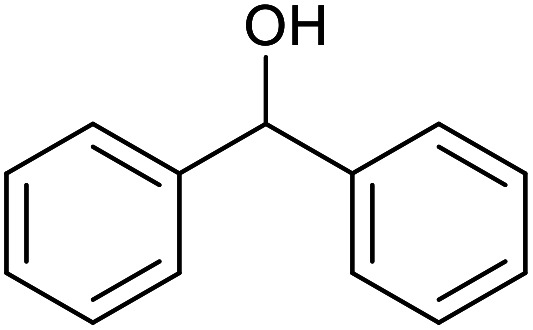	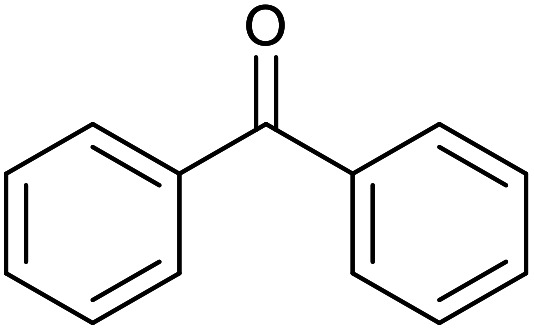	18	94	94
24^d^	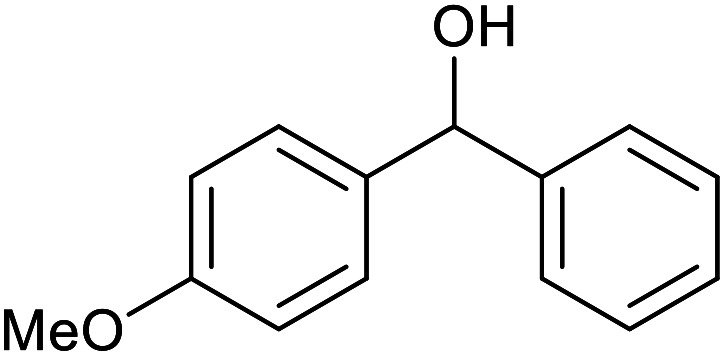	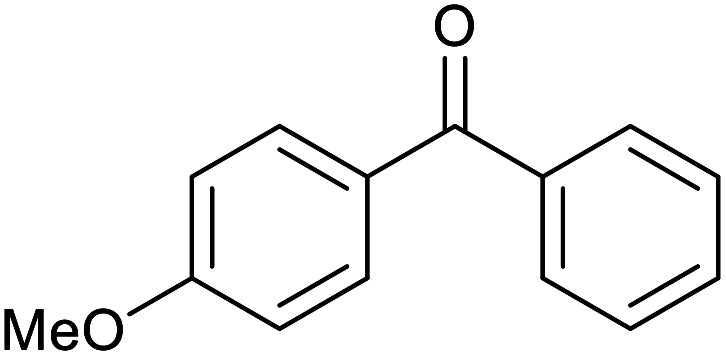	15	100	100
25^d^	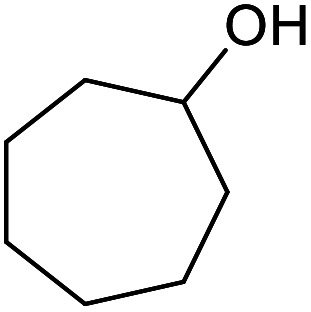	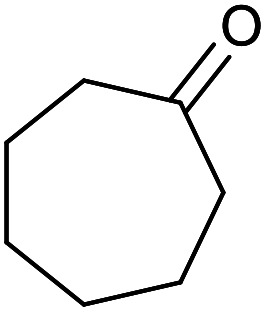	12	90	90
26^d^	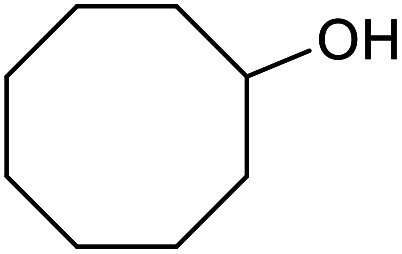	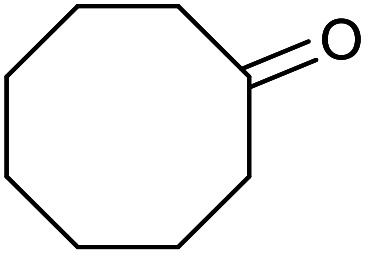	12	100	100
27^d^	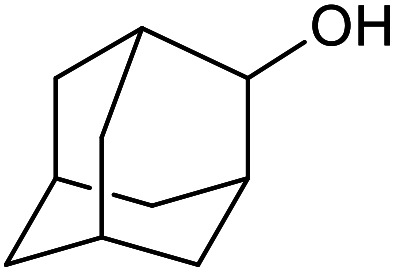	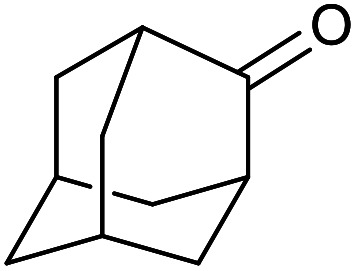	24	10	—
28^d^	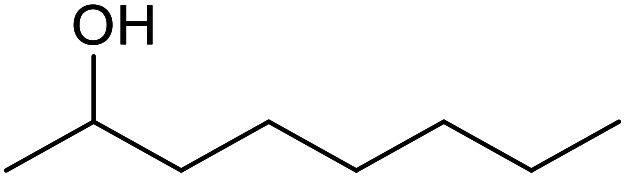	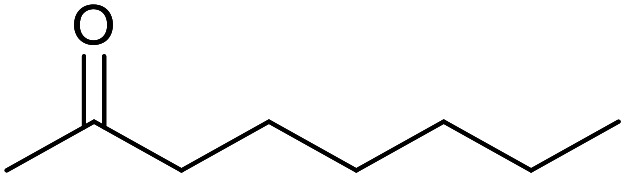	24	32	—
29^d^	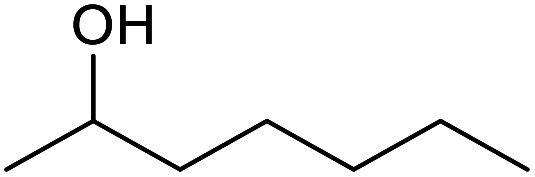	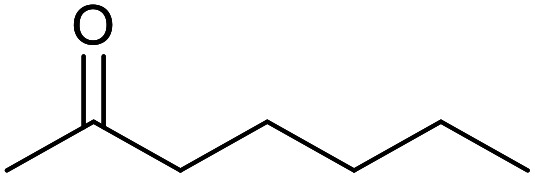	24	25	—

a 0.5 mL isopropanol was added.

b Catalyst (0.4 mol% [Pd]) and 90 °C.

c Catalyst (1 mol% [Pd]) and 90 °C.

d Catalyst (1 mol% [Pd]), 2.25 equiv. NaOH and 90 °C. 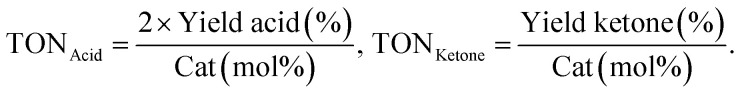

**Fig. 10:**
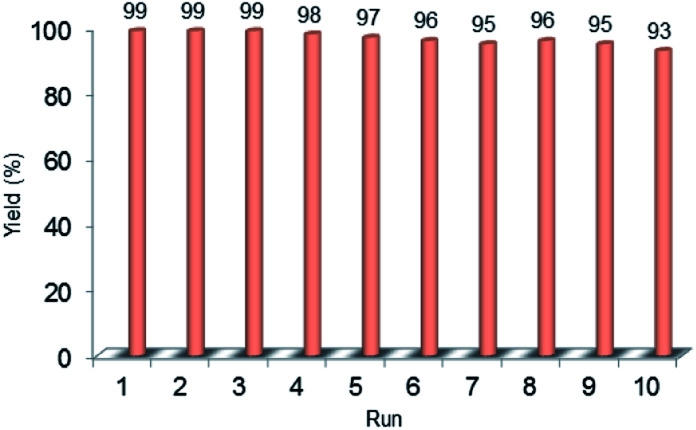
Recyclability chart of the aerobic oxidation of benzyl alcohol using the Pd@PPy/OMC catalyst.

Recyclability is considered as a crucial factor in developing novel heterogeneous catalysts from the viewpoint of reducing the cost of chemical processes and minimizing environmental destructive effects. To investigate the recyclability of our catalyst, several runs were performed using benzyl alcohol under the optimized reaction conditions. In each run of the reaction, after 2 hours, the Pd@PPy/OMC catalyst was simply recovered by filtration and then was washed with plenty of ethyl acetate and water to take away any remaining NaOH and organic contents. The recovered catalyst after drying was used for the next run of the oxidation reaction. As shown in Fig. 10, the catalyst was successfully utilized in ten reaction cycles and the yields of all runs were almost the same. These series of experiments confirmed that the catalyst could be reused without loss of its activity and selectivity. To check the possibility of the leaching of Pd particles into the solution, a hot filtration test was performed. In this way, the Pd@PPy/OMC catalyst was separated from the reaction vessel, after proceeding the oxidation reaction for 30 min (conversion 47%). Solution of the reaction was then transferred to another flask and allowed to stir under the optimum conditions for 12 h (oxygen atmosphere and 80 °C). Analysis of this catalyst-free reaction demonstrated <4% progress in the conversion of the reaction. Moreover, the Pd content of the recovered catalyst determined by AAS was almost the same as that of the fresh catalyst, confirming the strong interaction between the support and Pd species. N_2_ sorption analysis of the recovered catalyst showed an isotherm similar to that of the fresh catalyst, with a negligible change in the BET surface area and total pore volume (613 m^2^ g^−1^ and 1.17 cm^3^ g^−1^ for the *S*_BET_ and *V*_t_, respectively) (Fig. 7). This observation along with the TEM results strongly confirmed that the catalyst has mostly preserved its initial porosity and mesostructure during the catalysis and recycling processes. TEM analysis of the recovered catalyst also revealed that the palladium particles are almost uniformly distributed on the surface of the catalyst, within the range of 20–25 nm (Fig. 8c and d). However, the TGA analysis of the Re–Pd@PPy/OMC sample demonstrated a decomposition temperature lower than that of the Pd@PPy/OMC sample under the O_2_ atmosphere. Considering this claim that there is a correlation between the size of metal nanoparticles and their catalytic effect on the thermal decomposition of materials,^85^ we think that a slight change might have occurred on the size of the Pd nanoparticles during the oxidation reaction (Fig. 5).

To highlight the crucial role of the new PPy/OMC support in obtaining the observed excellent reactivity, the performance of the Pd@PPy/OMC catalyst was compared with a number of Pd-supported catalysts such as Pd@PPy, Pd@C and Pd@OMC. These comparative reactions were performed in the presence of 4-bromobenzyl alcohol as a test substrate and the Pd catalysts with the amount of 0.2 mol% Pd. For more consideration, the amount of Pd leached into the solutions during the oxidation reactions was also studied by calculating the difference between the loading of Pd on the catalysts and the loading of Pd on the recycled catalysts, shown in Table 4. As can be seen in Table 4, the Pd@PPy/OMC showed the highest activity and more importantly the best selectivity in comparison with others. What is appealing in this study is that in spite of using water as solvent of the reaction, the amount of 4-bromobenzoic acid remained below 50% by using of Pd@OMC as the catalyst, even after running the reaction for 24 h at 80 °C and under pure O_2_ atmosphere (Table 4, entry 2). The Pd@C and Pd@PPy catalysts showed even inferior activity under the identical conditions. These catalysts also demonstrated higher amount of the Pd leaching during the reaction (Table 4, entries 3 and 4). Higher yields of the products in the case of the Pd@OMC rather than the Pd@PPy and Pd@C catalysts obviously confirmed the beneficial effect of ordered mesochannels on the progress of the described oxidation reaction. Therefore, the superior performance and stability of the Pd@PPy/OMC catalyst may not only be attributed to the ordered mesoporous structure of its support, but it can imply to a great extent on the possible synergistic effects between its components and also its electronic properties. These extraordinary features could be led to the uniform and stable distribution of extremely surface active palladium particles, and the strong support/Pd interaction.

**Table 4:** Comparing the activity of the Pd@PPy/OMC catalyst with other Pd-supported catalysts in the oxidation of 4-bromobenzyl alcohol

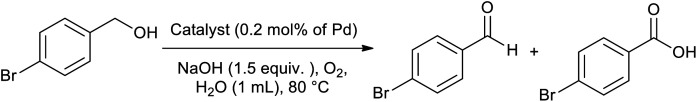						
Entry	Catalyst	Time (h)	Aldehyde (%)	Acid (%)	TON	Pd leached (%)
1	Pd@PPy/OMC	13	4	95	950	<1
2	Pd@OMC	24	18	47	470	5
3	Pd@C	24	21	2	20	11
4	Pd@PPy	24	7	0	0	14

**Scheme 2:**
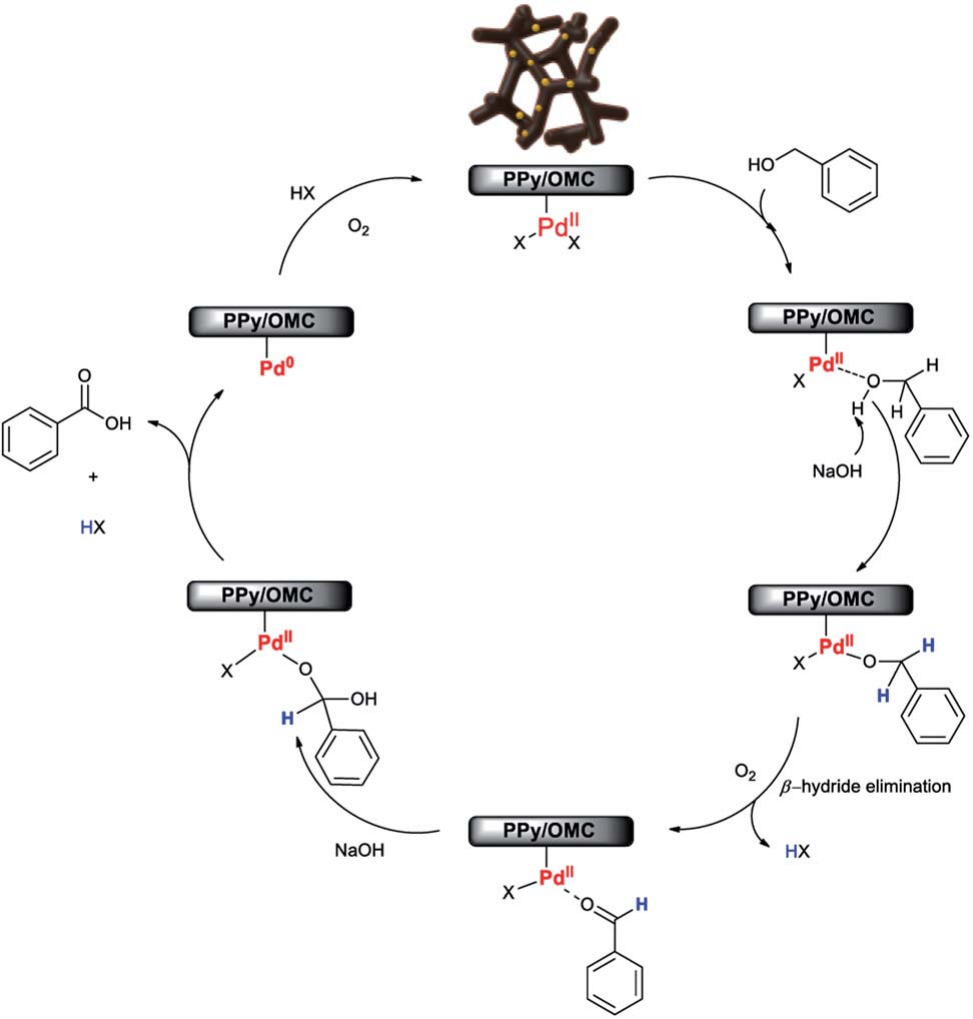
A plausible mechanism for the aerobic oxidation reaction of alcohols in the presence of the Pd@PPy/OMC catalyst.

Although, the precise mechanism for this reaction is not clear at this stage, according to the results obtained in this work and also study of some mechanisms reported by other research groups about the aerobic oxidation of alcohols by Pd-based catalysts,^28,37,86^ we here proposed a plausible mechanism for the oxidation reaction of alcohols to carboxylic acids using the Pd@PPy/OMC catalyst (Scheme 2). The results showed that the presence of the Pd catalyst and also NaOH is essential for the progress of the reaction (Table 2). In addition, XPS spectrum of our catalyst system clearly shows that both Pd(ii) and Pd(0) are simultaneously existed in the supported Pd nanoclusters (Fig. 9b).

Therefore, it seems reasonable to speculate that the catalytic cycle initiates with binding of alcohol substrate to the Pd(ii) species in the nanoclusters, forming Pd-bound alcohol. Deprotonation of the Pd-bound alcohol by NaOH, followed by β-hydride elimination results in the formation of aldehyde. In the next step, a geminal diol is generated by the nucleophilic addition of hydroxide anion to aldehyde in the presence of the hydroxide base in aqueous solution. Eventually, dehydrogenation of geminal diol on the Pd catalyst gives rise to the formation of carboxylic acid. The Pd(ii) catalyst is re-formed by the oxygenation of Pd(0) species, generated *via* the reductive elimination of palladium hydride species, in the presence of molecular oxygen and subsequent hydrolysis.

## Conclusions

With the aim of combining the unique properties of mesoporous carbons and polypyrrole, and also taking advantages of their synergistic effects, in this work we have focused on the preparation of a novel ordered mesoporous PPy/OMC composite through a two-step nanaocasting process. This PPy/OMC composite can be used as an efficient support for the immobilization of Pd species because of its amazing features including high surface area, open and ordered pore structure, and possessing nitrogen atoms in the structure. The resulting catalyst (Pd@PPy/OMC) was found to be an active catalyst in the oxidation of various primary and secondary alcohols in the presence of O_2_ oxidant in aqueous media. It is noteworthy that carboxylic acid compounds were prepared in high yields and selectivities. Moreover, the catalyst could be easily recovered without loss of its activity or selectivity, even after 10 recycling runs. Structure characterization of the PPy/OMC, Pd@PPy/OMC and recovered Pd@PPy/OMC materials by TEM, SEM, XRD, XPS FTIR, TGA, and N_2_ sorption analyses confirmed the ordered mesoporous structure and stability of the materials. We believe that this composite with its extraordinary properties possesses significant capabilities for application in other organic transformations.

## Conflicts of interest

There are no conflicts to declare.

## Supplementary Material

RA-010-C9RA10941B-s001

## References

[cit1] Sheldon R. A. , Arends I. W. C. E. and Hanefeld U., Green Chemistry and Catalysis, Wiley-VCH, Weinheim, 2007

[cit2] Enanche D. I., Edwards J. K., Landon P., Espria B. S., Carley A. F., Herzing A. A., Watanabe M., Kiely C., Knight J. D. W., Hutching G. J. (2006). Science.

[cit3] Kaizuka K., Miyamura H., Kobayashi S. (2010). J. Am. Chem. Soc..

[cit4] Gunanathan C., Ben-David Y., Milstein D. (2007). Science.

[cit5] Gunanathan C., Shimon L. J. W., Milstein D. (2009). J. Am. Chem. Soc..

[cit6] Hollingworth G. J. , Comprehensive Organic Functional Group Transformations, ed. A. R. Katritzky, O. Meth-Cohn, C. W. Rees and G. Pattenden, Elsevier Science, Oxford, 1995, pp. 81–109

[cit7] Patai S. , The Chemistry of Functional Groups, The Chemistry of Carboxylic Acids and Esters, Wiley, New York, 1969

[cit8] Benzoic Acid Market Size, Industry Analysis Report, Regional Outlook, Application Development, Price Trend, Competitive Market Share & Forecast, 2019-2025, https://www.gminsights.com/industryanalysis/benzoicacid-market

[cit9] Mahmood A., Robinson G. E., Powell L. (1999). Org. Process Res. Dev..

[cit10] Thottathil J. K., Moniot J. L., Mueller R. H., Wong M. K. Y., Kissick T. P. (1986). J. Org. Chem..

[cit11] Caron S., Dugger R. W., Ruggeri S. G., Ragan J. A., Ripin D. H. B. (2006). Chem. Rev..

[cit12] Tojo G. and Fernandez M., Oxidation of Primary Alcohols to Carboxylic Acids, Springer, New York, 2010

[cit13] Bilgrien C., Davis S., Drago R. S. (1987). J. Am. Chem. Soc..

[cit14] Kaneda K., Fujii M., Morioka K. (1996). J. Org. Chem..

[cit15] Marko I. E., Giles P. R., Tsukazaki M., Brown S. M., Urch C. J. (1996). Science.

[cit16] Stahl S. S., Thorman J. L., Nelson R. C., Kozee M. A. (2001). J. Am. Chem. Soc..

[cit17] Gamez P., Arends I. W., Reedijk J., Sheldon R. A. (2003). Chem. Commun..

[cit18] Backvall J.-E. , Modern Oxidation Methods, Wiley-VCH, 2004

[cit19] Mallat T., Baiker A. (2004). Chem. Rev..

[cit20] Zope B. N., Hibbitts D. D., Neurock M., Davis R. J. (2010). Science.

[cit21] Yu H., Ru S., Dai G., Zhai Y., Lin H., Han S., Wei Y. (2017). Angew. Chem., Int. Ed..

[cit22] Mallat T., Baiker A. (1994). Catal. Today.

[cit23] Mori K., Yamaguchi K., Hara T., Mizugaki T., Ebitani K., Kaneda K. (2002). J. Am. Chem. Soc..

[cit24] Stahl S. S. (2004). Angew. Chem., Int. Ed..

[cit25] Ng Y. H., Ikeda S., Harada T., Morita Y., Matsumura M. (2008). Chem. Commun..

[cit26] Davis S. E., Ide M. S., Davis R. J. (2013). Green Chem..

[cit27] Sheldon R. A., Arends I. W. C. E., Ten Brink G.-J., Dijksman A. (2002). Acc. Chem. Res..

[cit28] Ten Brink G.-J., Arends I. W. C. E., Sheldon R. A. (2000). Science.

[cit29] Kundu A., Buffin B. P. (2001). Organometallics.

[cit30] Ten Brink G.-J., Arends I. W. C. E., Sheldon R. A. (2002). Adv. Synth. Catal..

[cit31] Steinhoff B. A., Fix S. R., Stahl S. S. (2002). J. Am. Chem. Soc..

[cit32] Ebitani K., Fujie Y., Kaneda K. (1999). Langmuir.

[cit33] Kakiuchi N., Maeda Y., Nishimura T., Uemura S. (2001). J. Org. Chem..

[cit34] Uozumi Y., Nakao R. (2003). Angew. Chem., Int. Ed..

[cit35] Karimi B., Abedi S., Clark J. H., Budarin V. (2006). Angew. Chem., Int. Ed..

[cit36] Karimi B., Behzadnia H., Bostina M., Vali H. (2012). Chem.–Eur. J..

[cit37] An G., Ahn H., De Castro K. A., Rhee H. (2010). Synthesis.

[cit38] Karimi B., Khorasani M., Vali H., Vargas C., Luque R. (2015). ACS Catal..

[cit39] Ahmed M. S., Mannel D. S., Root T. W., Stahl S. S. (2017). Org. Process Res. Dev..

[cit40] Stahl S. S. , Powell A. B., Root T. W., Mannel D. S. and Ahmed M. S., *US Pat.*, 9593064 B2, Mar 14, 2017

[cit41] Stahl S. S. , Powell A. B., Root T. W., Mannel D. S. and Ahmed M. S., *US Pat.*, 20170137362 A1, May 18, 2017

[cit42] Tang L., Guo X., Li Y., Zhang S., Zha Z., Wang Z. (2013). Chem. Commun..

[cit43] Sapurina I., Stejskal J., Sedenkova I., Trchova M., Kovarova J., Hromadkova J., Kopecka J., Cieslar M., El-Nasr A. A., Ayad M. M. (2016). Synth. Met..

[cit44] Corma A. (1997). Chem. Rev..

[cit45] Fan Q., Li Y., Chan A. S. C. (2002). Chem. Rev..

[cit46] McKeown N. B., Budd P. M. (2006). Chem. Soc. Rev..

[cit47] Li J.-R., Kuppler Ryan J., Zhou H.-C. (2009). Chem. Soc. Rev..

[cit48] Zhang Y., Riduan S. N. (2012). Chem. Soc. Rev..

[cit49] Taguchi A., Schuth F. (2005). Microporous Mesoporous Mater..

[cit50] Perego C., Millini R. (2013). Chem. Soc. Rev..

[cit51] Fujita S., Inagaki S. (2008). Chem. Mater..

[cit52] Parlett C. M., Wilson K., Lee A. F. (2013). Chem. Soc. Rev..

[cit53] Ryoo R., Joo S. H., Kruk M., Jaroniec M. (2001). Adv. Mater..

[cit54] Kyotani T. (2000). Carbon.

[cit55] Schuth F. (2003). Angew. Chem., Int. Ed..

[cit56] Lee J., Kim J., Hyeon T. (2006). Adv. Mater..

[cit57] White R. J., Luque R., Budarin V. L., Clark J. H., Macquarrie D. J. (2009). Chem. Soc. Rev..

[cit58] Tasis D., Tagmatarchis N., Bianco A., Prato M. (2006). Chem. Rev..

[cit59] Choi M., Ryoo R. (2003). Nat. Mater..

[cit60] Pacheco-Catalan D. E., Smit M. A., Morales E. (2011). Int. J. Electrochem. Sci..

[cit61] Arshak K., Velusamy V., Korostynska O., Oliwa-Stasiak K., Adley C. (2009). IEEE Sens. J..

[cit62] Zhou C. F., Kumar S., Doyle C. D., Tour J. M. (2005). Chem. Mater..

[cit63] Kim C. H., Kim S.-S., Guo F., Hogan T. P., Pinnavaia T. J. (2004). Adv. Mater..

[cit64] Choi Y. S., Joo S. H., Lee S. A., You D. J., Kim H., Pak C., Chang H., Seung D. (2006). Macromolecules.

[cit65] Choi M., Lim B., Jang J. (2008). Macromol. Res..

[cit66] Kim T. W., Kleitz F., Paul B., Ryoo R. (2005). J. Am. Chem. Soc..

[cit67] Kleitz F., Choi S. H., Ryoo R. (2003). Chem. Commun..

[cit68] Zhang Z., Wang G., Li Y., Zhang X., Qiao N., Wang J., Zhou J., Liu Z., Hao Z. A. (2014). J. Mater. Chem. A.

[cit69] Kleitz F., Berube F., Guillet-Nicolas R., Yang C.-M., Thommes M. (2010). J. Phys. Chem. C.

[cit70] Dai W., Zheng M., Zhao Y., Liao S., Ji G., Cao J. (2010). Nanoscale Res. Lett..

[cit71] Liu Q., Wang A., Xu J., Zhang Y., Wang X., Zhang T. (2008). Microporous Mesoporous Mater..

[cit72] Luo J. Y., Wang Y. G., Xiong H. M., Xia Y. Y. (2007). Chem. Mater..

[cit73] Zhang A. Q., Xiao Y. H., Lu L. Z., Wang L. Z., Li F. (2013). J. Appl. Polym. Sci..

[cit74] Oliveira H. P., Sydlik S. A., Swager T. M. (2013). J. Phys. Chem. C.

[cit75] Okan B. S., Zanjani J. S. M., Letofsky-Papst I., Cebeci F. C., Menceloglu Y. Z. (2015). Mater. Chem. Phys..

[cit76] Holade Y., MacVittie K., Conlon T., Guz N., Servat K., Napporn T. W., Kokoh K. B., Katz E. (2014). Electroanalysis.

[cit77] Sellin R., Clacens J. M., Coutanceau C. (2010). Carbon.

[cit78] Ma Z. C., Yang H. Q., Qin Y., Hao Y. J., Li G. (2010). J. Mol. Catal. A: Chem..

[cit79] Lu X., Bian X., Nie G., Zhang C., Wang C., Wei Y. (2012). J. Mater. Chem..

[cit80] Macfie G., Cooper A., Cardosi M. F. (2011). Electrochim. Acta.

[cit81] Estrade-Szwarckopf H. (2004). Carbon.

[cit82] Buitrago-Sierra R., Jesus Garcia-Fernandez M., Mercedes Pastor-Blas M., Sepulveda-Escribano A. (2013). Green Chem..

[cit83] Qiu L., Liu F., Zhao L., Yang W., Yao J. (2006). Langmuir.

[cit84] Dijksman A., Marino-González A., Payeras A. M., Arends I. W. C. E., Sheldon R. A. (2001). J. Am. Chem. Soc..

[cit85] Ghimbeu C. M., Sopronyi M., Sima F., Delmotte L., Vaulot C., Zlotea C., Paul-Boncour V., Le Meins J. M. (2015). Nanoscale.

[cit86] Mueller J. A., Goller C. P., Sigman M. S. (2004). J. Am. Chem. Soc..

