# Supplementary material for: Palladium supported on a novel ordered mesoporous polypyrrole/carbon nanocomposite as a powerful heterogeneous catalyst for the aerobic oxidation of alcohols to carboxylic acids and ketones on water
Source: RSC Adv. 2020 Apr 3;10(23):13616–31. doi: 10.1039/c9ra10941b (PMC9051566; doi:10.1039/c9ra10941b)
Supplement: RA-010-C9RA10941B-s001 [file RA-010-C9RA10941B-s001.pdf]

Electronic Supplementary Material (ESI) for RSC Advances.
This journal is © The Royal Society of Chemistry 2020

Supporting Information for

Palladium Supported on a Novel Ordered Mesoporous Polypyrrole/Carbon Nanocomposite as a Powerful Heterogeneous Catalyst for the Aerobic Oxidation of Alcohols to Carboxylic Acids and Ketones on Water

Nasim Ganji,[a] Babak Karimi,*[a, b] Sepideh Najafvand-Derikvandi[a] and Hojatollah Vali[c]

[a.] *Department of Chemistry, Institute for Advanced Studies in Basic Sciences (IASBS), PO-Box 45195-1159, Gava-zang, Zanjan 45137-6731, Iran.*

[b.] *Research Center for Basic Sciences & Modern Technologies (RBST), Institute for Advanced Studies in Basic Sciences (IASBS), Zanjan 45137-66731, Iran.*

[c.] *Department of Anatomy and Cell Biology and Facility for Electron Microscopy Research McGill University, Montreal, Quebec, H3A 2A7, Canada.*

Contents

1. Materials 4

2. Experimental Procedures 4

2.1. Preparation of the KIT-6 silica template 4

2.2. Preparation of the OMC/KIT-6 composite 4

2.3. Preparation of the PPy/OMC composite 4

2.4. Preparation of the Pd@PPy/OMC catalyst 4

2.5. General procedure for the oxidation of primary alcohols 4

2.6. General procedure for the oxidation of secondary alcohols 4

3. Characterization 5

4. Figures 10

Figure S1. Nitrogen adsorption-desorption isotherms of KIT-6, OMC/KIT-6 and PPy/OMC/KIT-6 materials 6

Figure S2. BJH-Plots of the KIT-6, OMC/KIT-6 and PPy/OMC/KIT-6 materials 6

Figure S3. Nitrogen adsorption-desorption isotherms of the OMC and PPy/OMC materials 7

Figure S4. BJH-Plots of the OMC and PPy/OMC materials 7

Figure S5. Nitrogen adsorption-desorption isotherms of the PPy/OMC, Pd@PPy/OMC and Re-Pd@PPy/OMC materials 8

Figure S6. BJH-Plots of the PPy/OMC, Pd@PPy/OMC and Re-Pd@PPy/OMC materials 8

Figure S7. Low-angle X-ray diffraction patterns of the KIT-6, PPy/OMC and Pd@PPy/OMC materials 9

Figure S8. HRTEM image of KIT-6 10

Figure S9. HRTEM image of the OMC material 11

Figure S10. HRTEM image of the PPy/OMC composite 12

Figure S11. HRTEM image of the Pd@PPy/OMC catalyst (0.5 µm) 13

Figure S12. HRTEM image of the Pd@PPy/OMC catalyst (100 nm) 14

Figure S13. HRTEM image of the recovered Pd@PPy/OMC catalyst (200 nm) 15

Figure S14. HRTEM image of the recovered Pd@PPy/OMC catalyst (200 nm) 16

Figure S15. Typical FESEM image of the pristine OMC (200 nm) 17

Figure S16. Typical FESEM image of the pristine OMC (1 µm) 18

Figure S17. Typical FESEM image of the PPy/OMC (200 nm) 19

Figure S18. Typical FESEM image of the PPy/OMC (1 µm) 20

Figure S19. Thermogravimetric analysis curve of the OMC material under the nitrogen atmosphere 21

Figure S20. Thermogravimetric analysis curve of the PPy/OMC material under the nitrogen atmosphere 21

Figure S21. Thermogravimetric analysis curve of the Pd@PPy/OMC material under the nitrogen atmosphere 22

Figure S22. Thermogravimetric analysis curve of the recovered Pd@PPy/OMC material under the nitrogen atmosphere 22

Figure S23. Thermogravimetric analysis curve of PPy under the nitrogen atmosphere.. 23

Figure S24. Thermogravimetric analysis curve of the OMC material under the oxygen atmosphere 23

Figure S25. Thermogravimetric analysis curve of the PPy/OMC material under the oxygen atmosphere............................. 24

Figure S26. Thermogravimetric analysis curve of the Pd@PPy/OMC material under the oxygen atmosphere 24

Figure S27. Thermogravimetric analysis curve of the recovered Pd@PPy/OMC material under the oxygen atmosphere 25

Figure S28. Thermogravimetric analysis curve of the recovered Pd@PPy/OMC material under the oxygen atmosphere 25

Figure S29. Infrared spectra of PPy, OMC and PPy/OMC powders 26

Figure S30. XPS survey spectrum of the Pd@PPy/OMC 26

Figure S31. High-resolution XPS spectrum of Palladium 27

Figure S32. High-resolution XPS spectrum of nitrogen.. 28

Figure S33. High-resolution XPS spectrum of carbon 29

Figure S34. Gas chromatogram of benzyl alcohol (Table 3 Entry 1) 30

Figure S35. Gas chromatogram of 4-methoxybenzyl alcohol (Table 3 Entry 2) 30

Figure S36. Gas chromatogram of 4-isopropylbenzyl alcohol (Table 3 Entry 3) 31

Figure S37. Gas chromatogram of 4-methylbenzyl alcohol (Table 3 Entry 4) 31

Figure S38. Gas chromatogram of 4-(methylthio)benzyl alcohol (Table 3 Entry 5) 32

Figure S39. Gas chromatogram of 4-chlorobenzyl alcohol (Table 3 Entry 6) 32

Figure S40. Gas chromatogram of 4-bromobenzyl alcohol (Table 3 Entry 7) 33

Figure S41. Gas chromatogram of 4-nitrobenzyl alcohol (Table 3 Entry 8) 33

Figure S42. Gas chromatogram of 3-methylbenzyl alcohol (Table 3 Entry 9) 34

Figure S 43. Gas chromatogram of 3-chlorobenzyl alcohol (Table 3 Entry 10) 34

Figure S44. Gas chromatogram of 3-nitrobenzyl alcohol (Table 3 Entry 11) 35

Figure S45. Gas chromatogram of 3-methoxybenzyl alcohol (Table 3 Entry 12) 35

Figure S46. Gas chromatogram of 2-chlorobenzyl alcohol (Table 3 Entry 13) 36

Figure S47. Gas chromatogram of 2-methoxybenzyl alcohol (Table 3 Entry 14) 36

Figure S48. Gas chromatogram of 2-methylbenzyl alcohol (Table 3 Entry 15) 37

Figure S49. Gas chromatogram of 2,4-dichlorobenzyl alcohol (Table 3 Entry 16) 37

Figure S50. Gas chromatogram of cinnamyl alcohol (Table 3 Entry 17) 38

Figure S51. Gas chromatogram of 3-phenyl-1-propanol (Table 3 Entry 18) 38

Figure S52. Gas chromatogram of 2-phenyl-1-ethanol (Table 3 Entry 19) 39

Figure S53. Gas chromatogram of 1-octanol (Table 3 Entry 20) 39

Figure S54. Gas chromatogram of 1-heptanol (Table 3 Entry 21) 40

Figure S55. Gas chromatogram of 1-phenyl-1-ethanol (Table 3 Entry 22) 40

Figure S56. Gas chromatogram of benzhydrol (Table 3 Entry 23) 41

Figure S57. Gas chromatogram of 4-methoxybenzhydrol (Table 3 Entry 24) 41

Figure S58. Gas chromatogram of cycloheptanol (Table 3 Entry 25) 42

Figure S59. Gas chromatogram of cycloheptanol (Table 3 Entry 26) 42

1. Materials

Pluronic P123 ($EO_{20}PO_{70}EO_{20}$, EO = ethylene oxide, PO = propylene oxide) was obtained from Aldrich. $Pd(OAc)_2$ was purchased from Acros Organics. Tetraethyl orthosilicate (TEOS), hydrochloric acid (37%), sulfuric acid (98%) and also solvents were obtained from Merck Company and were utilized without further purification. Pyrrole was purchased from Aldrich and distilled under vacuum for more purification.

2. Experimental Procedures

2.1. Preparation of an ordered mesoporous silica template (KIT-6): KIT-6, an ordered mesoporous silica template, was prepared according to the procedures reported in literature.[1,2] In this connection, 5.5 g of P123 and 11.0 g of hydrochloric acid (37 wt%) were added to 201 g of deionized water and the resulting mixture was stirred for 6 h at 35 ˚C to obtain a homogeneous solution. Then, 5.5 g of n-butanol was poured into the solution and after 1 h, 11.44 g of TEOS was also added to it. Stir of the mixture was continued at 35 ˚C for 24 h. Next, the mixture was transferred into a teflon-lined autoclave and placed under static condition at 130 ˚C for 36 h. Content of the autoclave was filtered and washed with plenty of deionized water and ethanol. Finally, KIT-6 was obtained as a white solid by removing the P123 template through the calcination at 550 ˚C for 5 h under the air atmosphere.

2.2. Synthesis of the OMC/KIT-6 composite: Inspired by the Hao's work[3], an OMC/KIT-6 composite was obtained as follows. The synthesized KIT-6 template was dried at 100 ˚C for 12 h and was also degassed under vacuum for 1h to remove moisture. 1.25 g sucrose and 0.14 g H_2SO_4 (98 wt%) were added to 5 mL deionized water and the resulting solution was poured into a flask containing 1 gr degassed KIT-6 and the mixture was then sonicated for 45 min. The mixture was placed in an oven at 100 ˚C, and after 6 h, temperature of the oven increased to 160 ˚C and the material was heated in this temperature for 6 h. The brown solid was then carbonized at 800 ˚C for 1 h under the argon atmosphere to obtain the OMC/KIT-6 composite.

2.3. Preparation of the PPy/OMC composite: Channels of the OMC/KIT-6 composite generated during the carbonization process could be used for the deposition of PPy chains through the primary impregnation of Py monomers into the channels and then their *in-situ* polymerization. For this purpose, the OMC/KIT-6 composite was degassed under vacuum for 30 min. The composite was then impregnated with an H_2O:EtOH (1:1) solution containing an appropriate amount of Py monomers, calculated according to the pore volume of the OMC/KIT-6 composite. Flask of the reaction was placed into an ultrasonic bath for 45 min. The mixture was subsequently stirred for 24 h, with the aim of further penetration of Py monomers into the pores of the OMC/KIT-6 material. Extra solvents of the mixture were then removed under vacuum at 45 ˚C. Resulting solid was cooled in an ice bath and then, 200 mL HCl solution (1 mol.L^{-1}) containing ammonium persulfate (APS) as an oxidizing agent (molar ratio APS:Py 1.2:1) was added to it. The mixture was stirred at 0-5 ˚C for 24 h to complete the polymerization process. Finally, the black product was filtered and washed with plenty of deionized water and ethanol. Silica template of the as-synthesized PPy/OMC/KIT-6 composite was removed using a 10 wt% HF aqueous solution. Finally, the PPy/OMC composite was washed with deionized water and ethanol, and dried at 80 ˚C for 12 h.
In addition, an OMC material was obtained by removing the KIT-6 template from the initial OMC/KIT-6 composite to compare with the PPy/OMC composite.

2.4. Preparation of the Pd@PPy/OMC catalyst: 100 mg of the PPy/OMC composite was dispersed in 7 mL THF and sonicated for 45 min. Subsequently, 4.5 mg $Pd(OAc)_2$ (0.02 mmol) dissolved in 3 mL THF was dropwisely added to the suspension and the mixture was stirred at room temperature for 12 h. Finally, the obtained Pd@PPy/OMC catalyst was filtered and washed with THF several times and was dried at 80 ˚C for 12 h.

2.5. Oxidation of primary alcohols using the Pd@PPy/OMC catalyst: Primary alcohol (0.5 mmol), Pd@PPy/OMC (0.2 mol %), NaOH (30 mg, 0.75 equiv.) and H_2O (0.5 mL) were added into a two-necked flask. A condenser containing a balloon filled with pure oxygen was placed on the reaction flask. After stirring at 80 ˚C for an appropriate time, the mixture was

quenched with some drop of HCl and then filtered. The solution was extracted with H_2O and ethyl acetate and its organic layer was dried using anhydrous Na_2SO_4. Yield of the reaction was directly obtained by GC, without further purification of the product.

2.6. Oxidation of secondary alcohols using the Pd@PPy/OMC catalyst: Secondary alcohol (0.5 mmol), Pd@PPy/OMC (1 mol%), NaOH (90 mg, 2.25 equiv.) and H_2O (0.5 mL) were mixed in a two-necked flask. A condenser containing a balloon filled with pure oxygen was placed on the reaction flask. After stirring at 90 ˚C for an appropriate time, the reaction mixture was filtered and the solution was extracted with ethyl acetate and H_2O. Finally, the organic layer containing the product was dried over anhydrous Na_2SO_4. Yield of the reaction was directly obtained by GC, without further purification of the product.

3. Characterization: N_2 sorption analysis was performed by a Belsorp analyzer (BELMAX, Japan) at 77 K. Samples were degassed at 373 K for 10 h before the measurements. Specific surface area of the materials was obtained from the relative pressure range 0.05-0.15 and their pore size distribution (PSD) was determined by the use of the Barrett-Joyner-Halenda (BJH) method from the adsorption branch. Additionally, total pore volumes were assigned using the adsorbed volume at $P/P_0 \approx 0.995$. Pore structure of the samples was studied by Philips CM-200 and Titan Krios TEM instruments. Surface morphology of the materials was investigated using high resolution scanning electron microscopy (HR-SEM) images taken by a TeScan-Mira III ultrahigh resolution cold field emission scanning electron microscope. X-ray powder diffraction (XRD) analysis was performed using a XPERT-PRO MPD diffractometer ($Cu_{K\alpha}$ radiation) in the range of 0.8 to 10 degree. XPS spectra of the materials were recorded on a Kratos Analytical X-ray photoelectron spectrometer. Thermogravimetric (TGA) analysis was obtained using a NETZSCH STA 409 PC/PG instrument (Germany) at scan rates of 10 $K.min^{-1}$, with typically 5 mg sample from 25 to 800 ˚C under both of the N_2 and O_2 atmosphere. Nitrogen content of the materials was determined by elemental analysis (C, H, N) using the vario-EL CHNS instrument. Fourier transform infrared (FTIR) spectra of the materials were attained using a Bruker vector 22 instrument in the range of 400 and 4000 cm^{-1}. Yield of oxidation reactions was determined using a Varian CP-3800 gas chromatograph instrument (GC) equipped with a capillary column and a flame-ionization detector (FID).

1. T. W. Kim, F. Kleitz, B. Paul and R. Ryoo, *J. Am. Chem. Soc*., 2005, **127**, 7601-7610.
2. F. Kleitz, S. H. Choi and R. Ryoo, *Chem. Commun*., 2003, 2136-2137.
3. Z. Zhang, G. Wang, Y. Li, X. Zhang, N. Qiao, J. Wang, J. Zhou, Z. Liu and Z. A. Hao, *J. Mater. Chem. A*, 2014, **2**, 16715-16722.

Figures

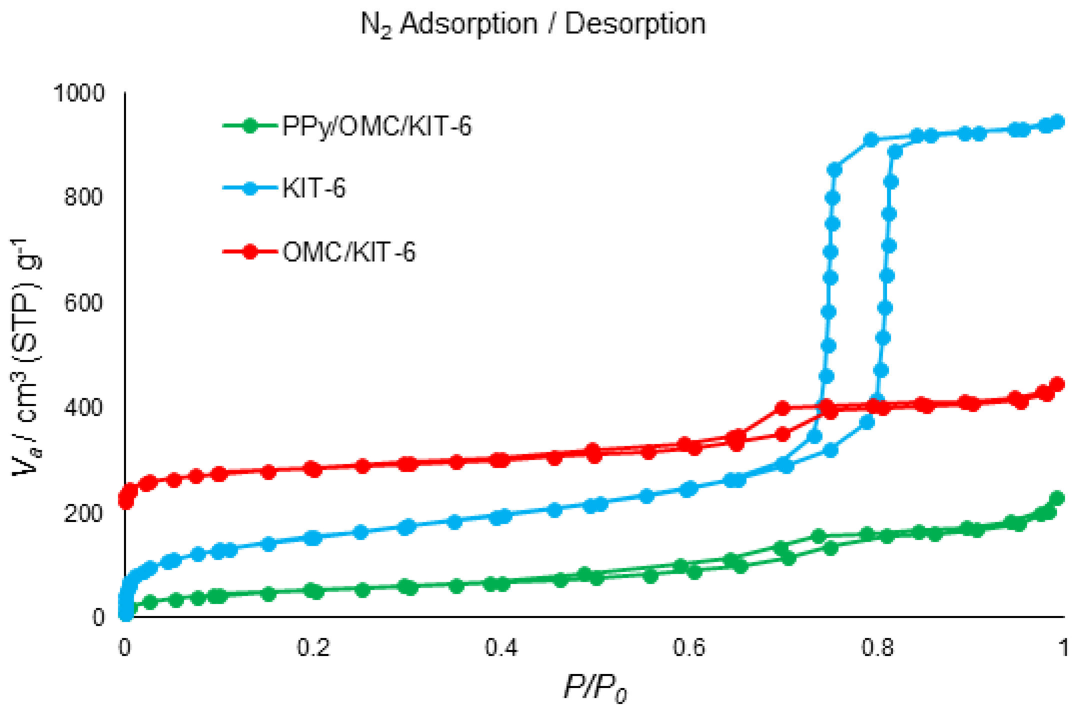

Figure S1. Nitrogen adsorption-desorption isotherms of the KIT-6, OMC/KIT-6 and PPy/OMC/KIT-6 materials

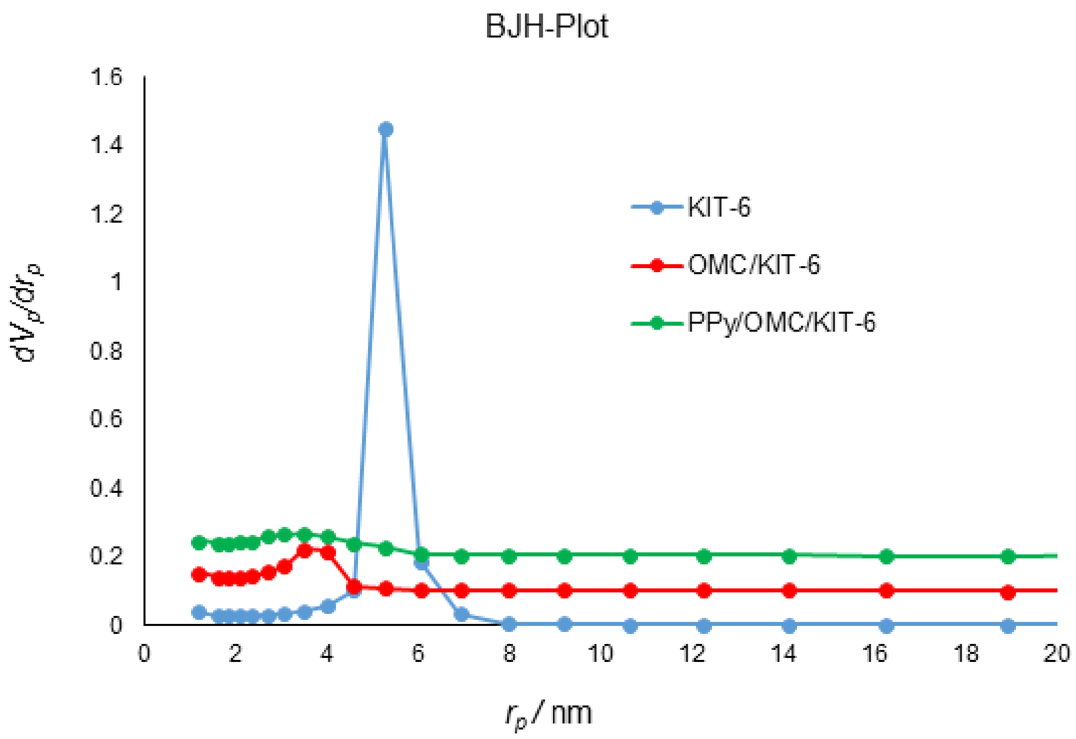

Figure S2. BJH-Plots of the KIT-6, OMC/KIT-6 and PPy/OMC/KIT-6 materials

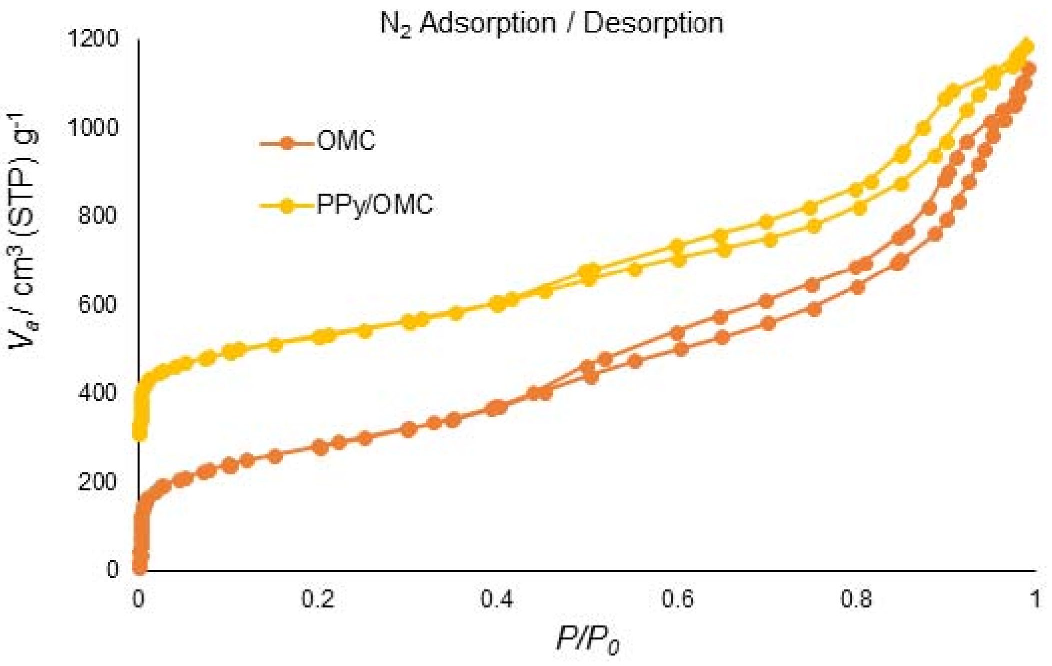

Figure S3. Nitrogen adsorption-desorption isotherms of the OMC and PPy/OMC materials

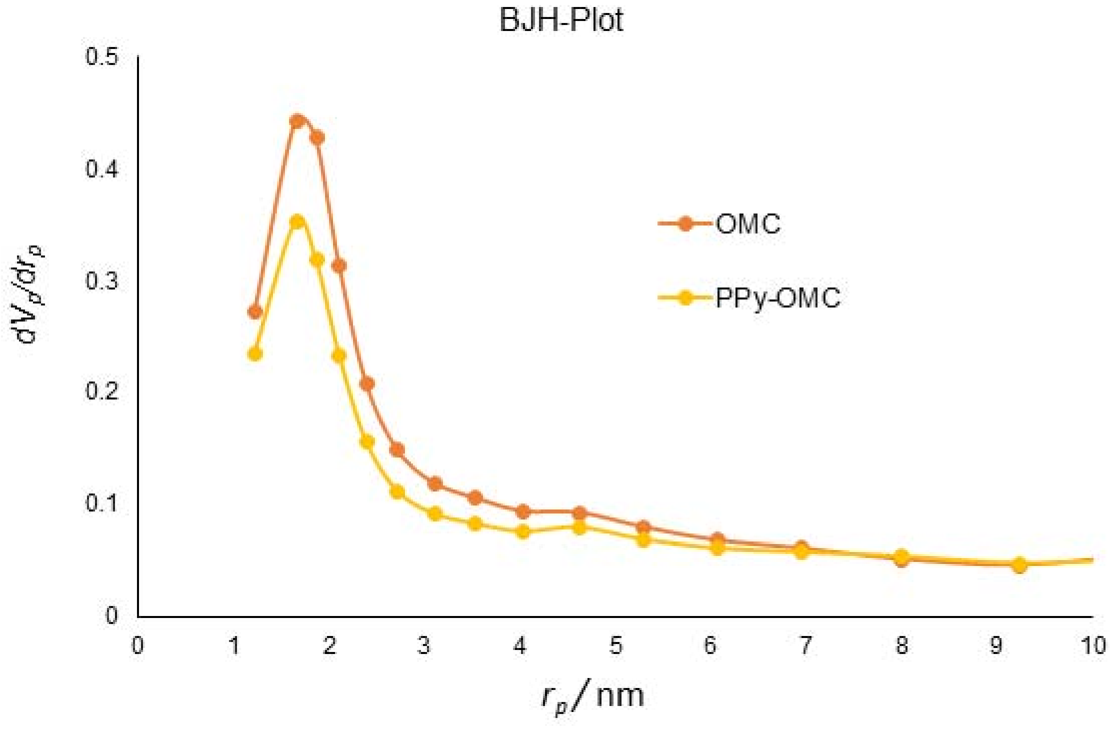

Figure S4. BJH-Plots of the OMC and PPy/OMC materials

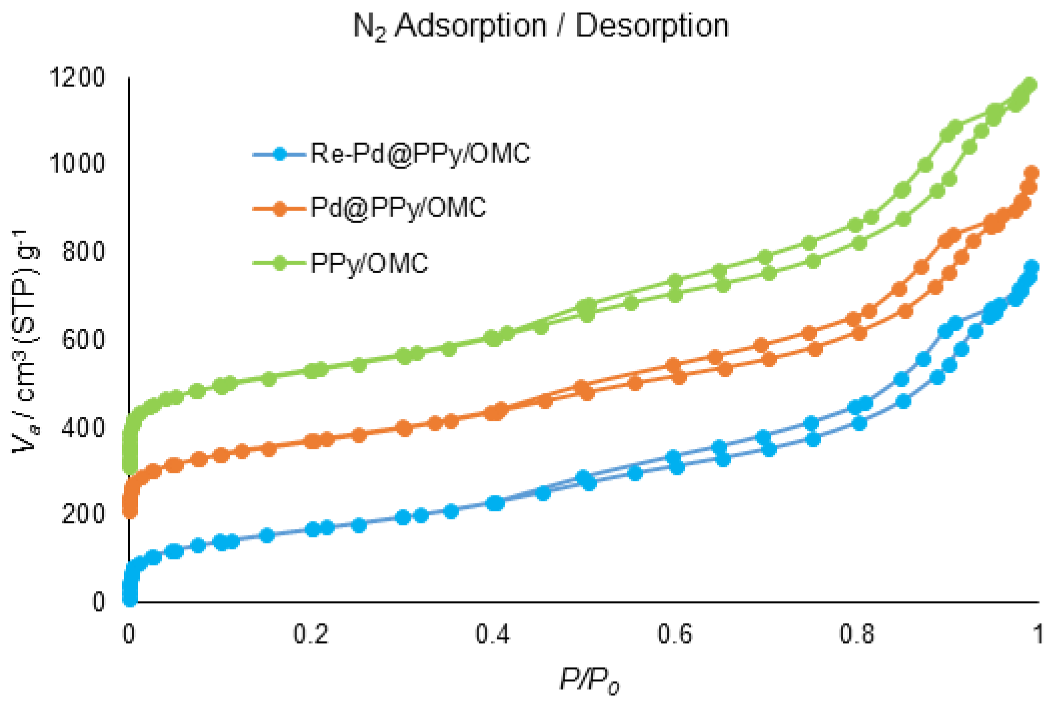

Figure S5. Nitrogen adsorption-desorption isotherms of the PPy/OMC, Pd@PPy/OMC and Re-Pd@PPy/OMC materials

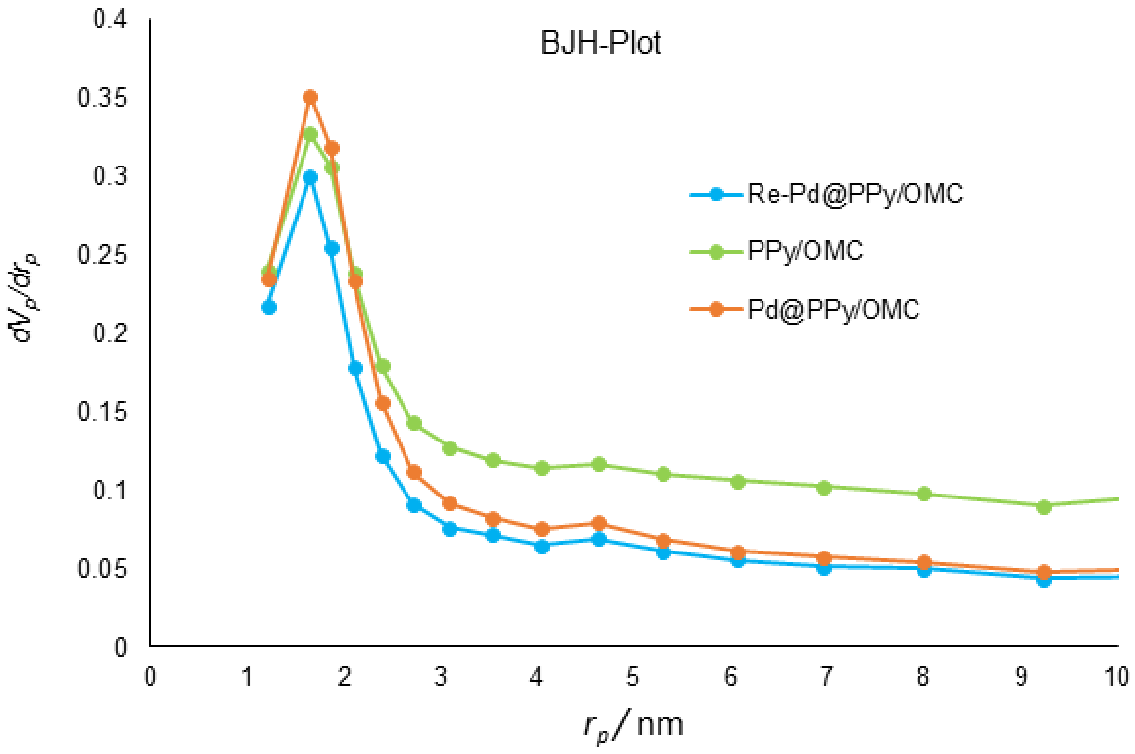

Figure S6. BJH-Plots of the PPy/OMC, Pd@PPy/OMC and Re-Pd@PPy/OMC materials

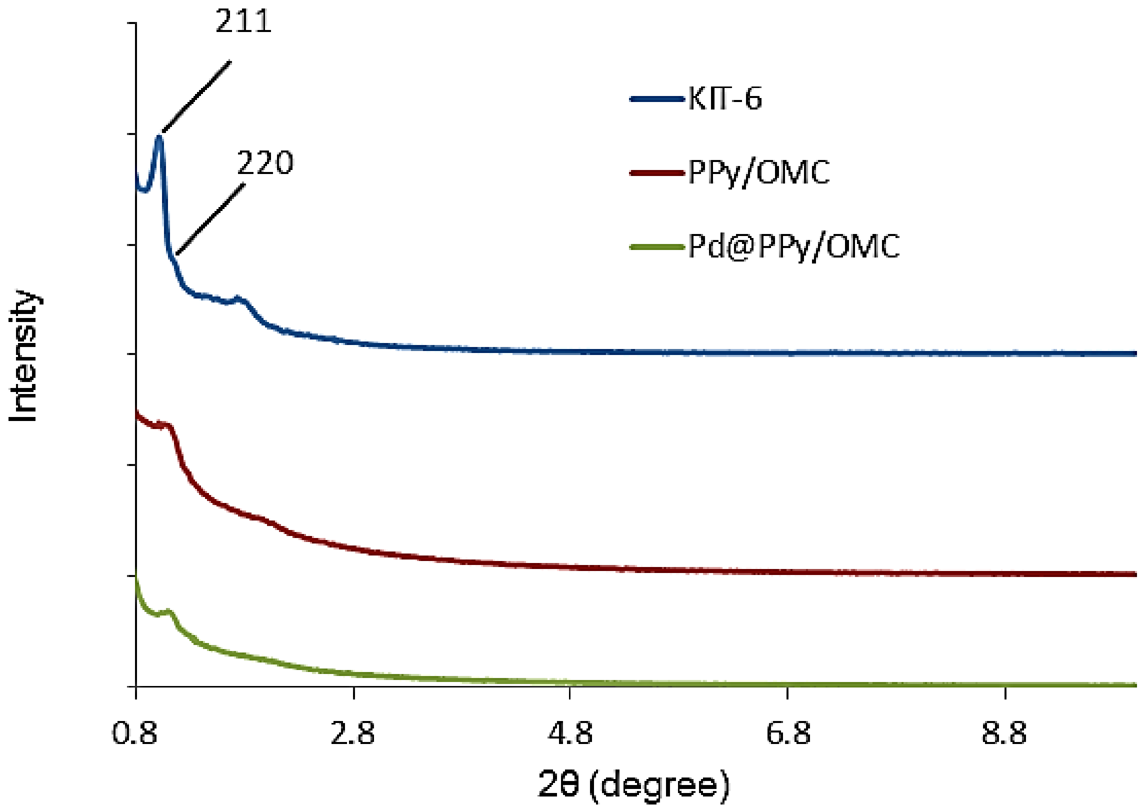

Figure S7. Low-angle X-ray diffraction patterns of the KIT-6, PPy/OMC and Pd@PPy/OMC materials

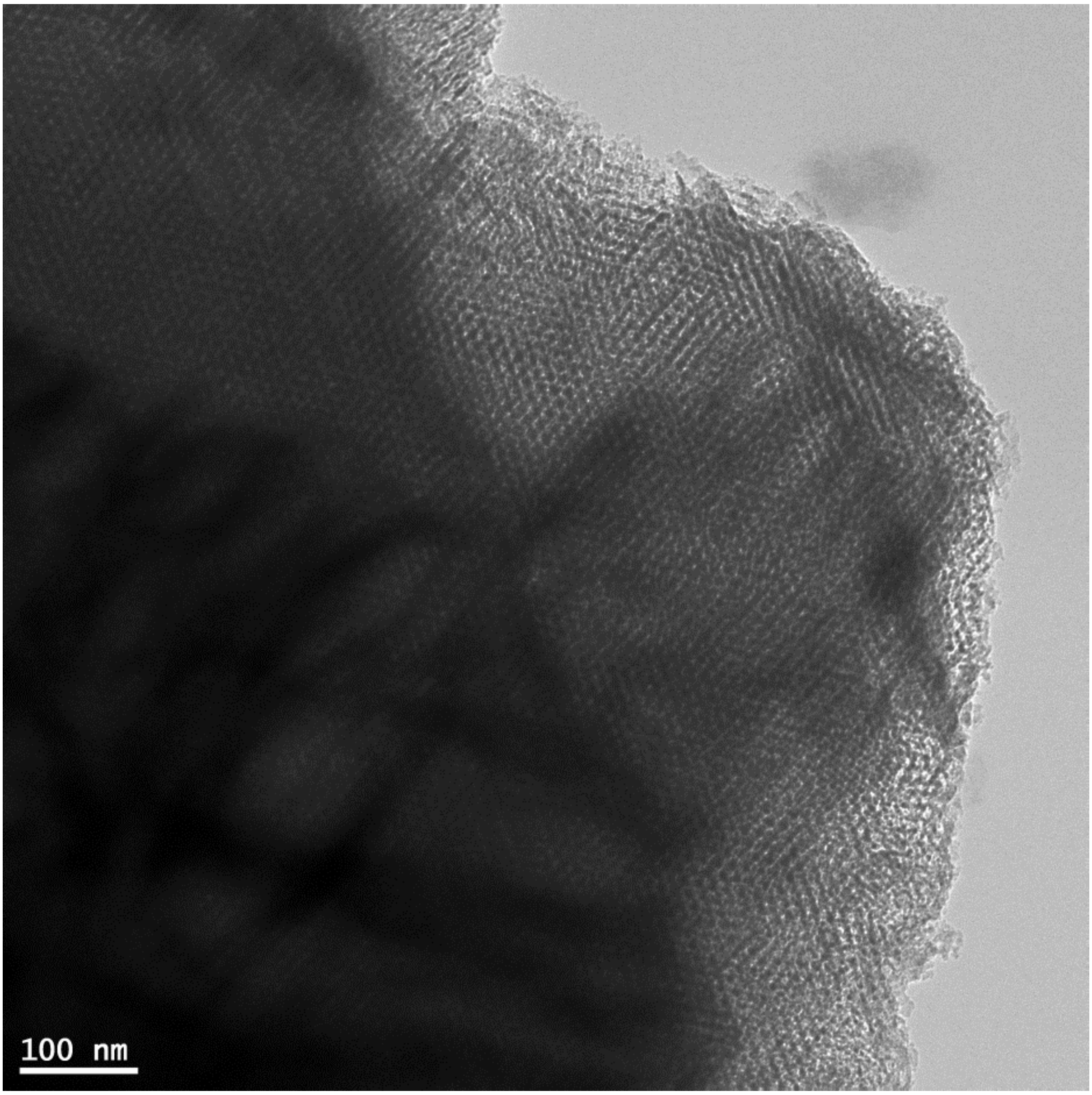

Figure S8. TEM image of KIT-6

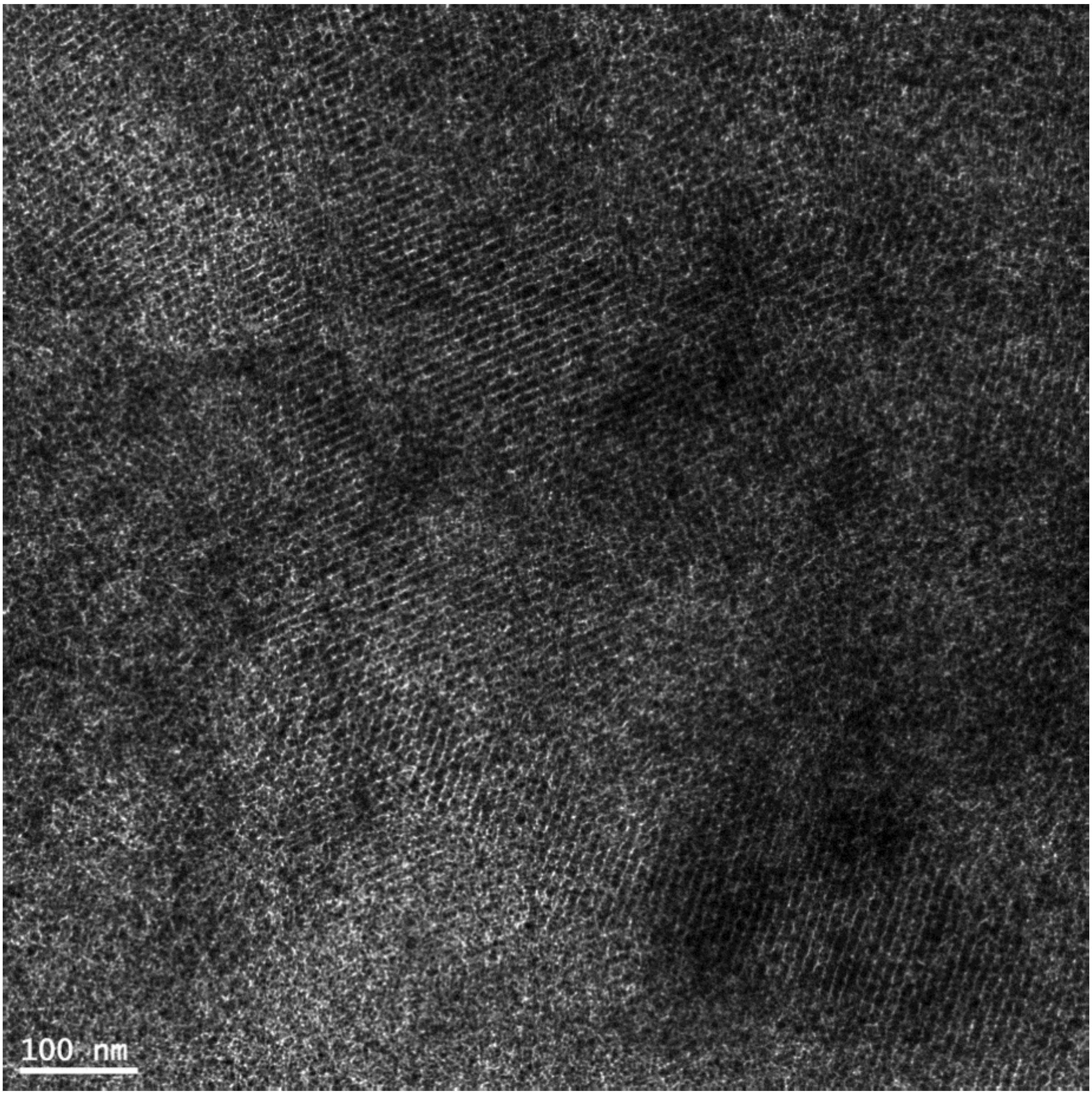

Figure S9. TEM image of the OMC material

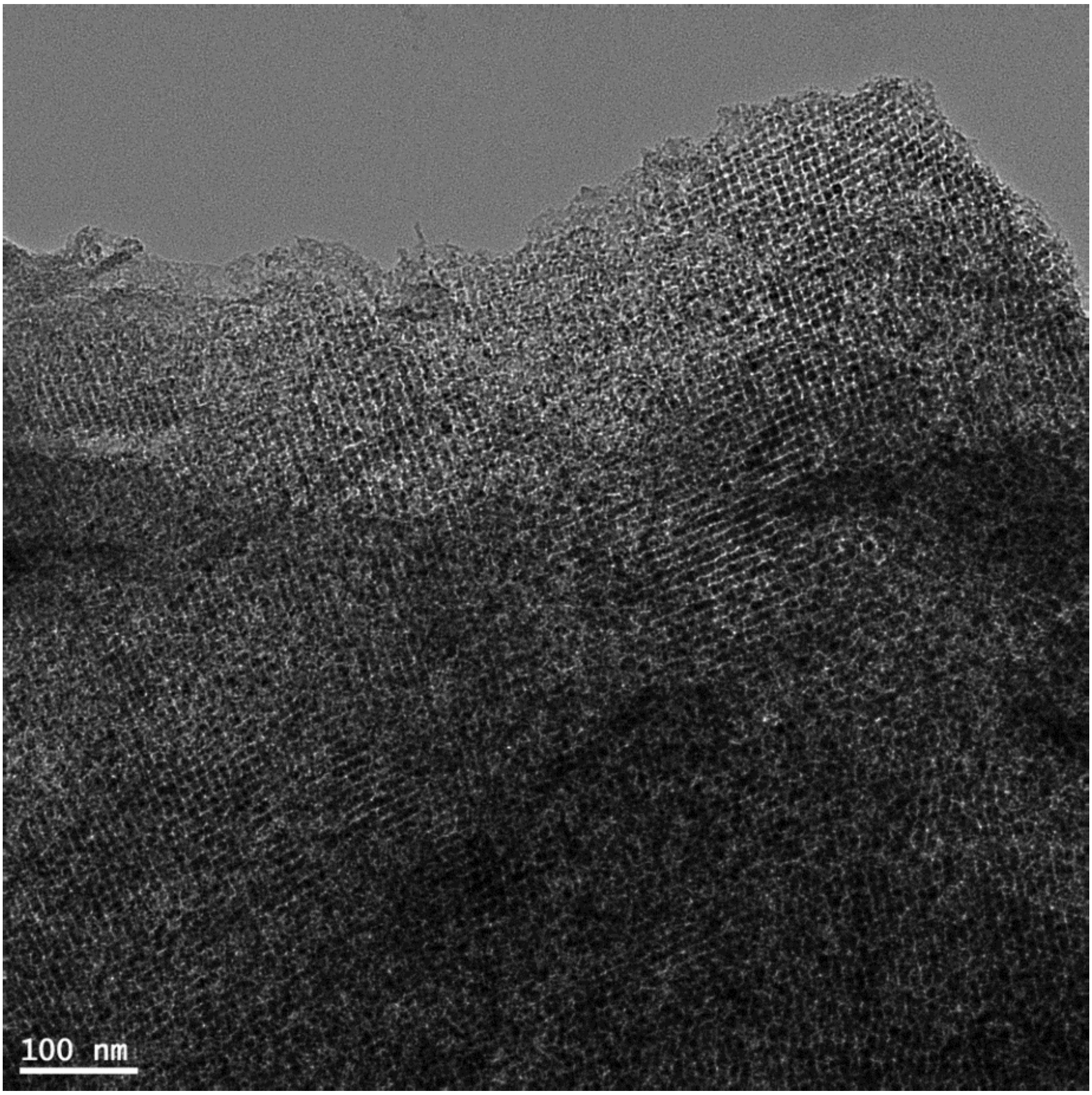

Figure S10. TEM image of the PPy/OMC composite

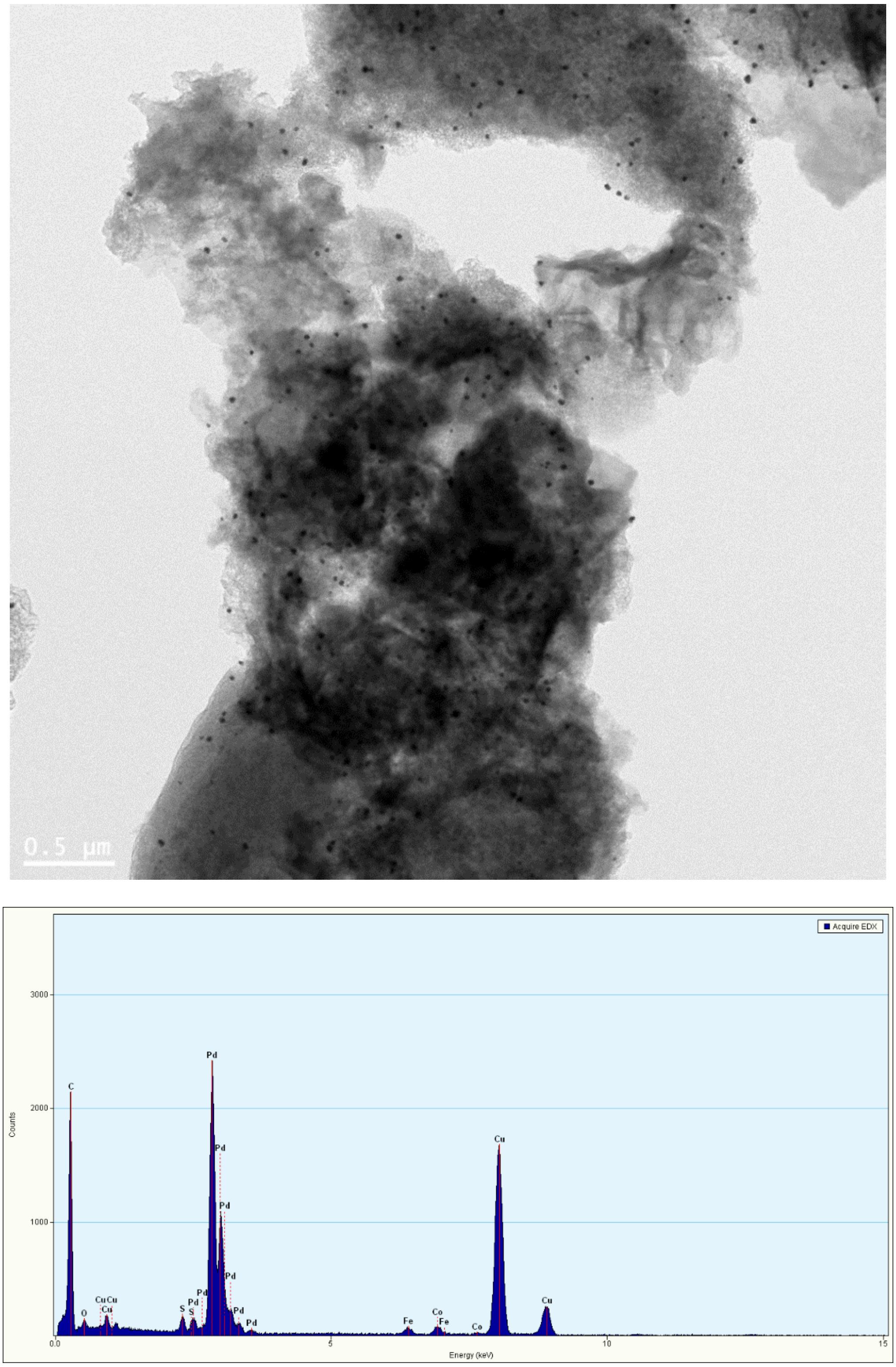

Figure S11. TEM image and EDX analysis of of the Pd@PPy/OMC catalyst (0.5 μm)

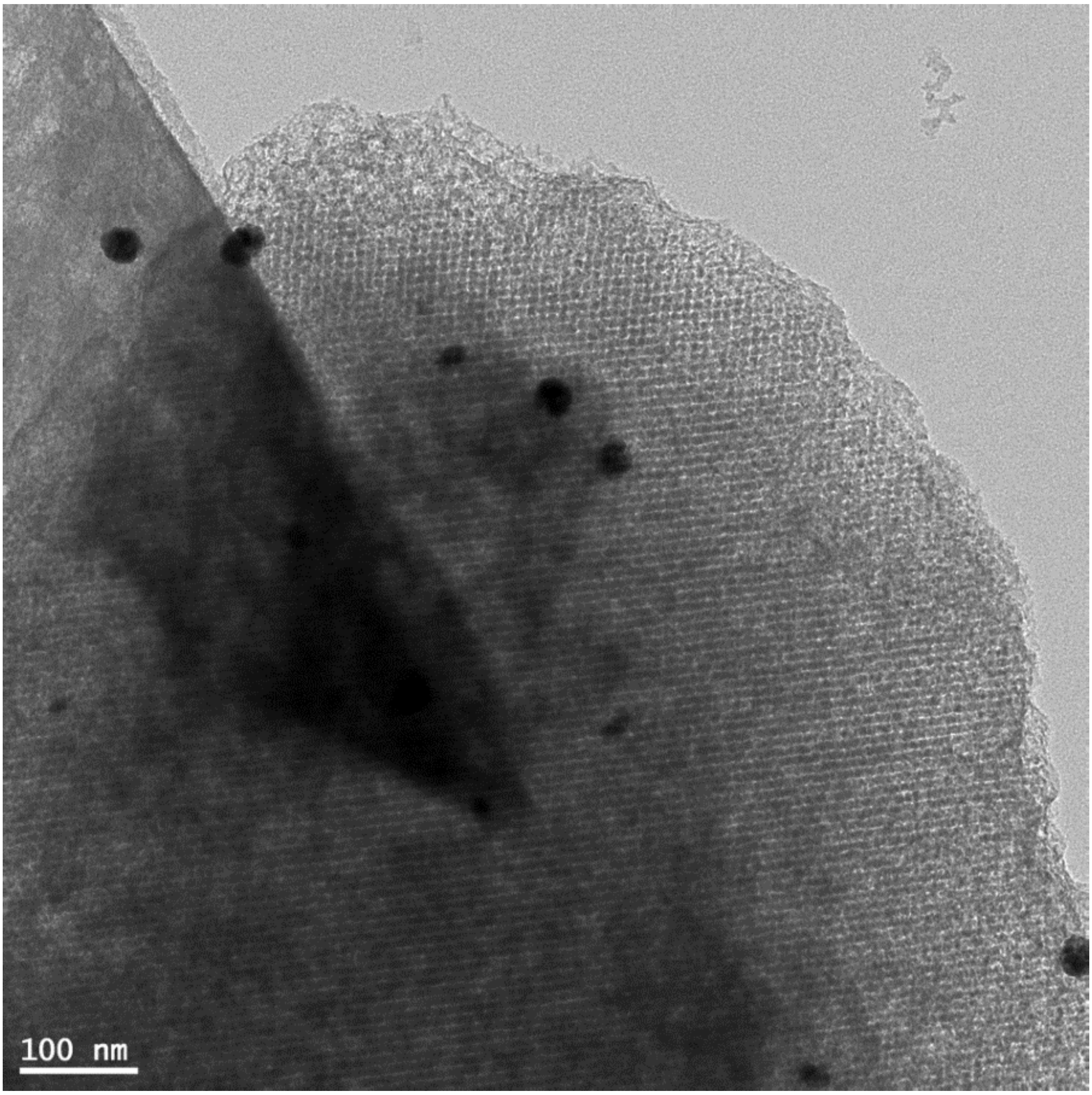

Figure S12. TEM image of the Pd@PPy/OMC catalyst (100 nm)

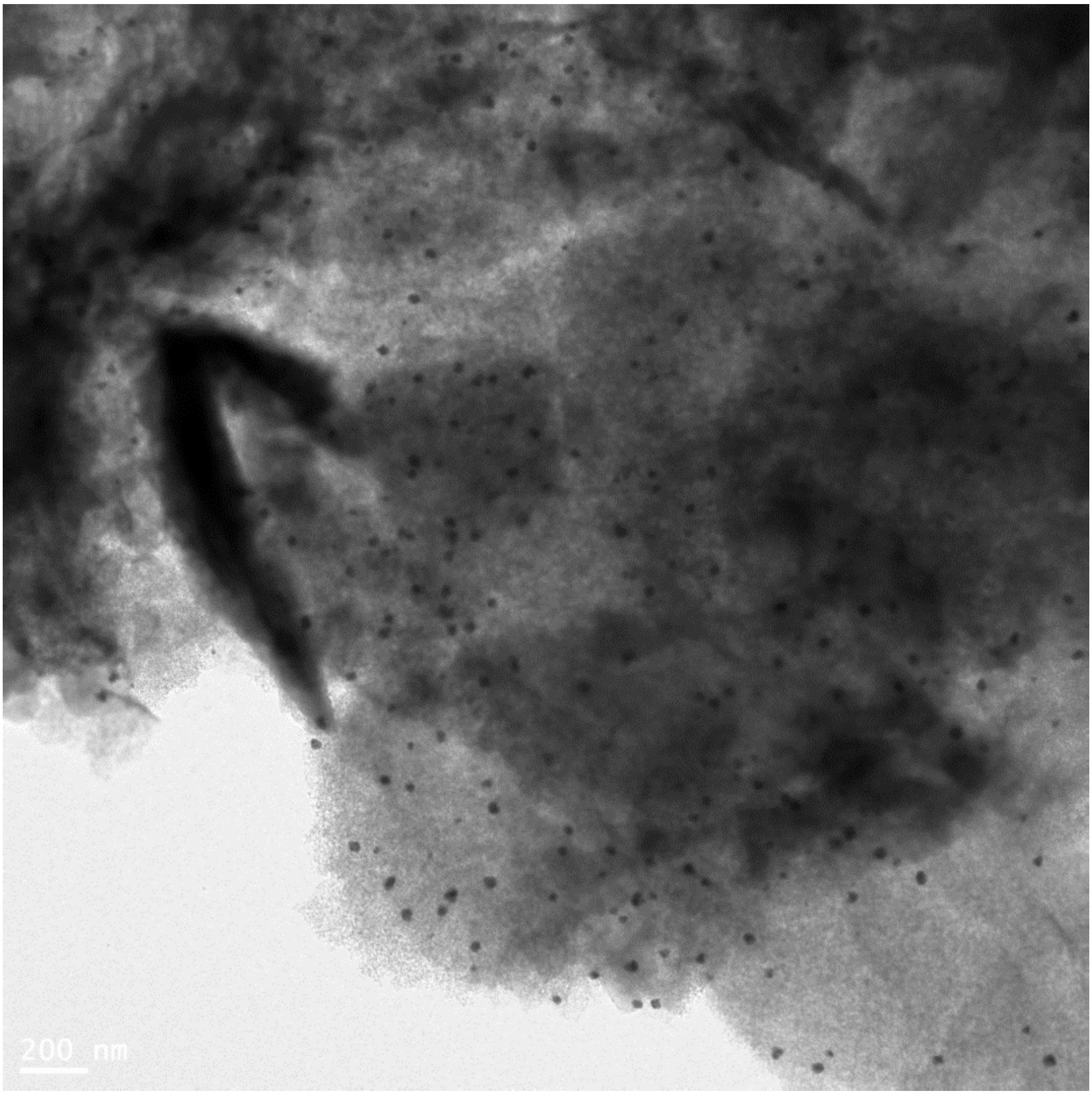

Figure S13. TEM image of the recovered Pd@PPy/OMC catalyst (200 nm)

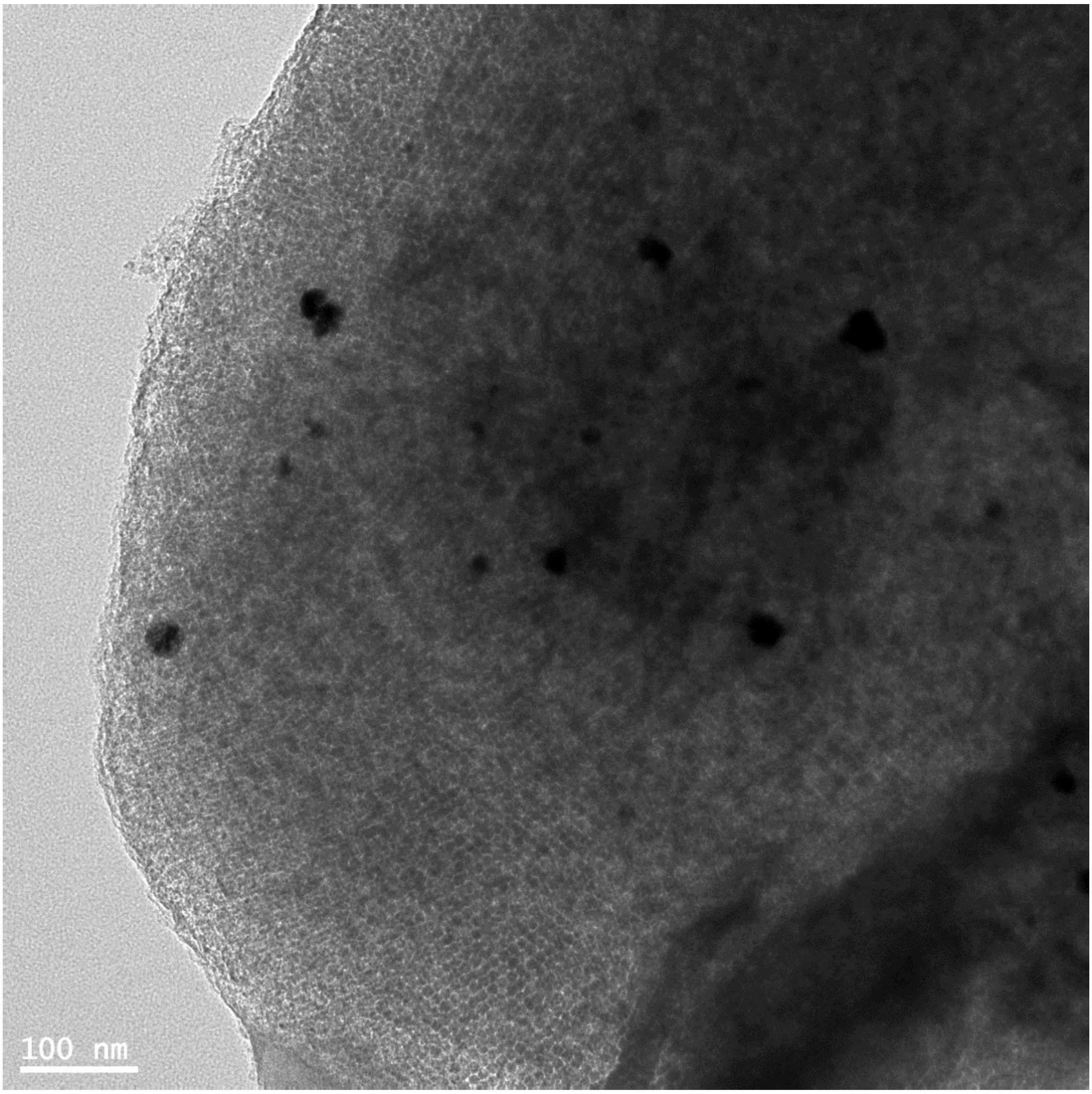

Figure S14. TEM image of the recovered Pd@PPy/OMC catalyst (100 nm)

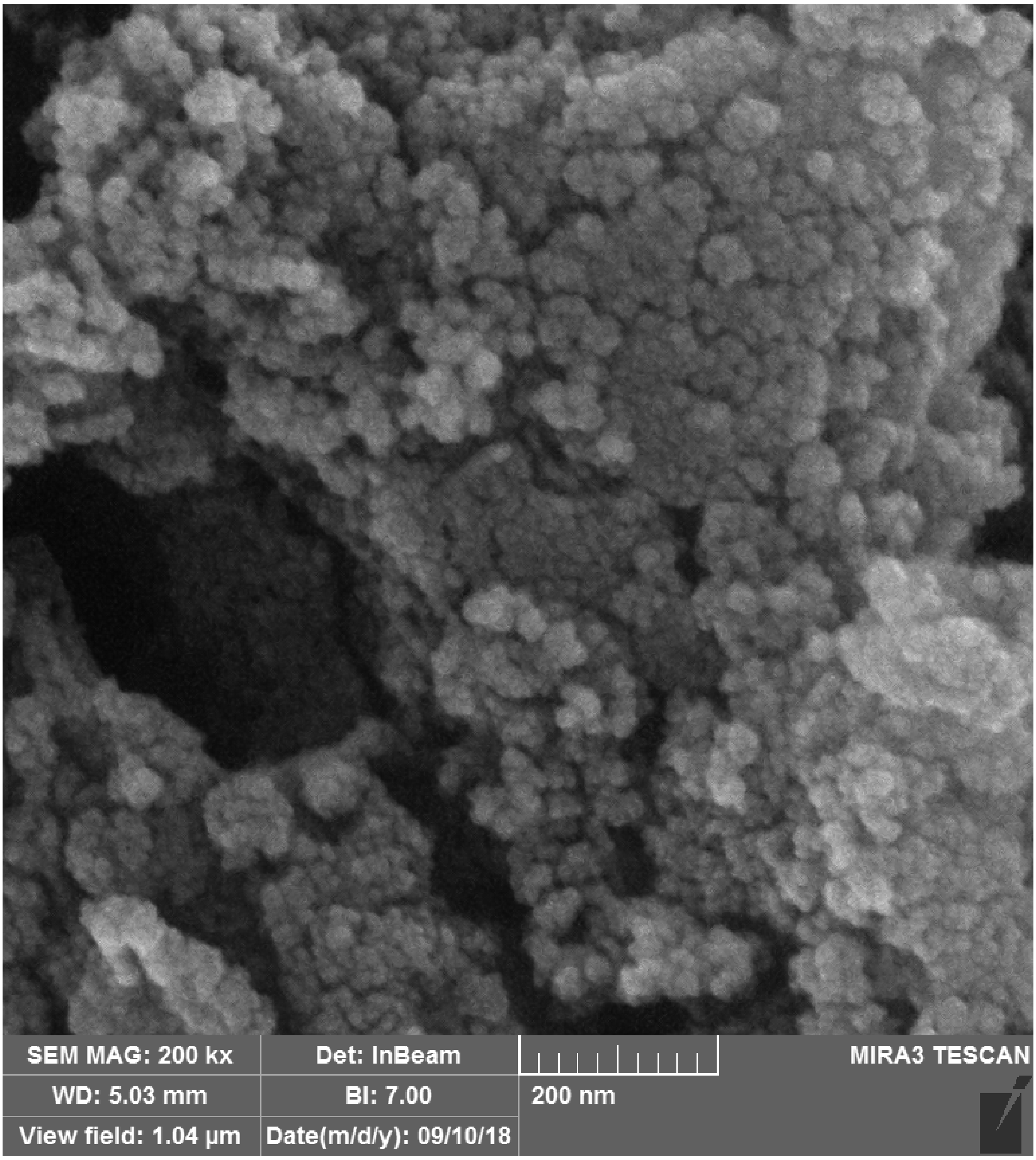

Figure S15. Typical FESEM image of the pristine OMC (200 nm)

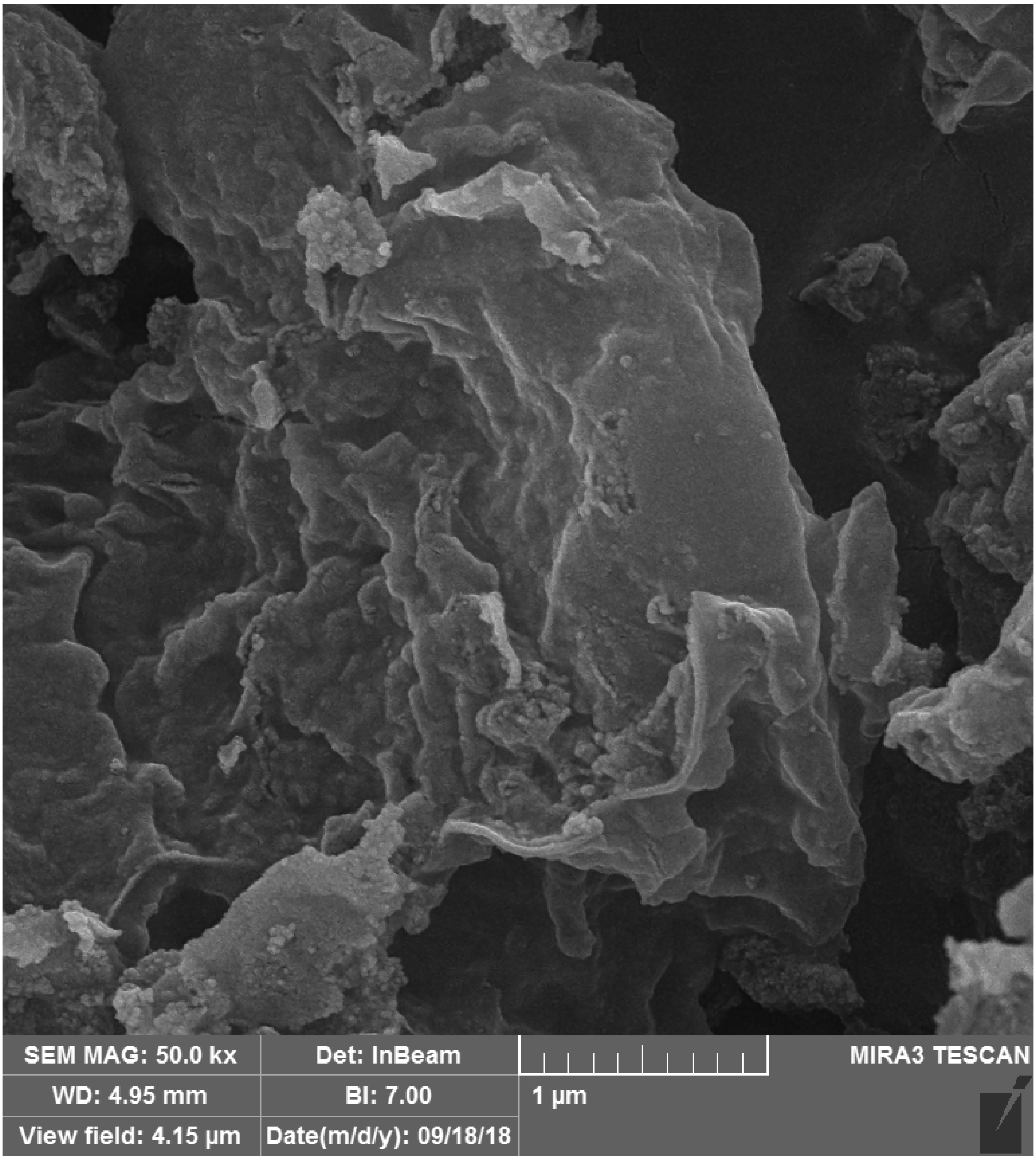

Figure S16. Typical FESEM image of the pristine OMC (1 μm)

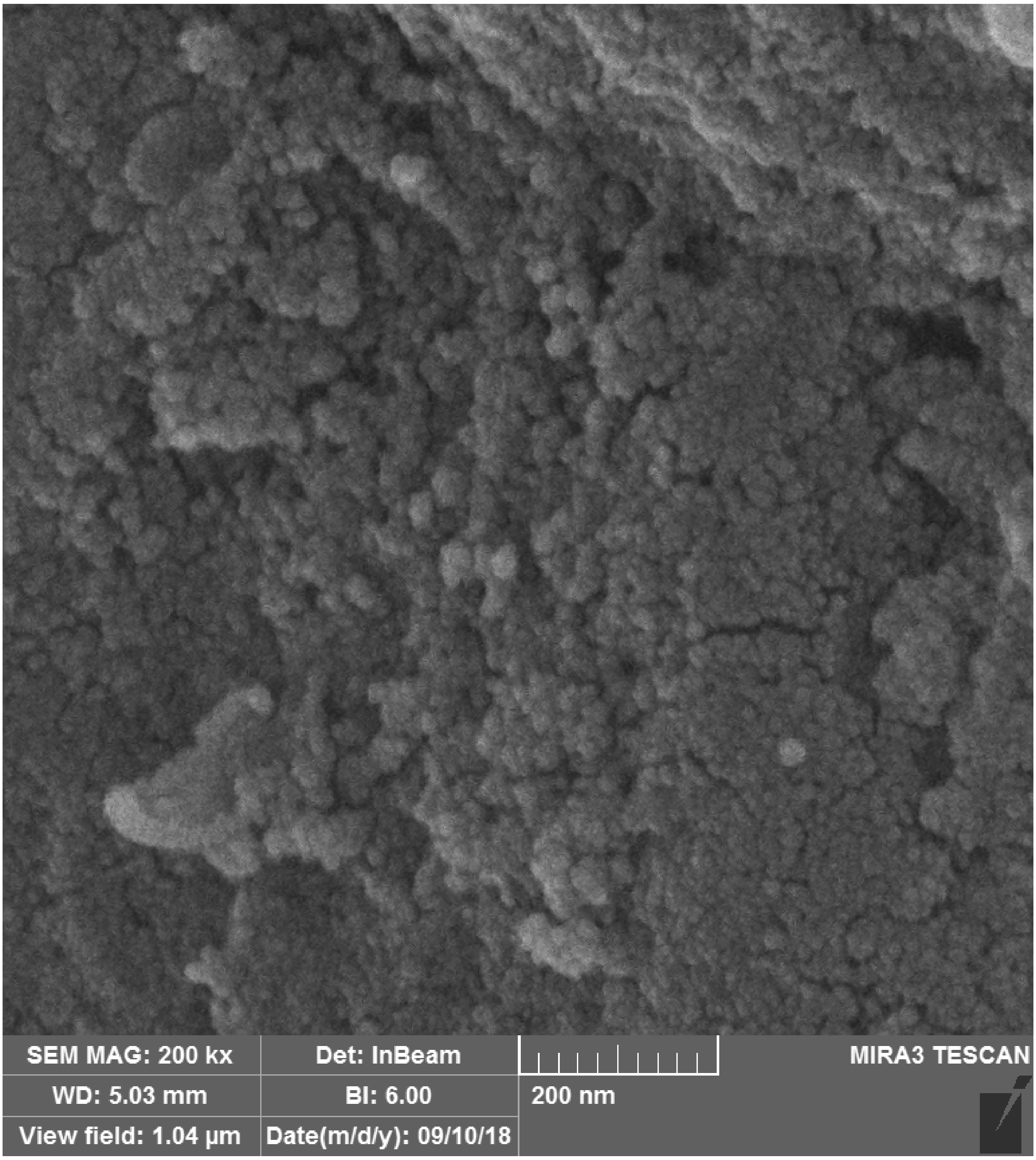

Figure S17. Typical FESEM image of the PPy/OMC (200 nm)

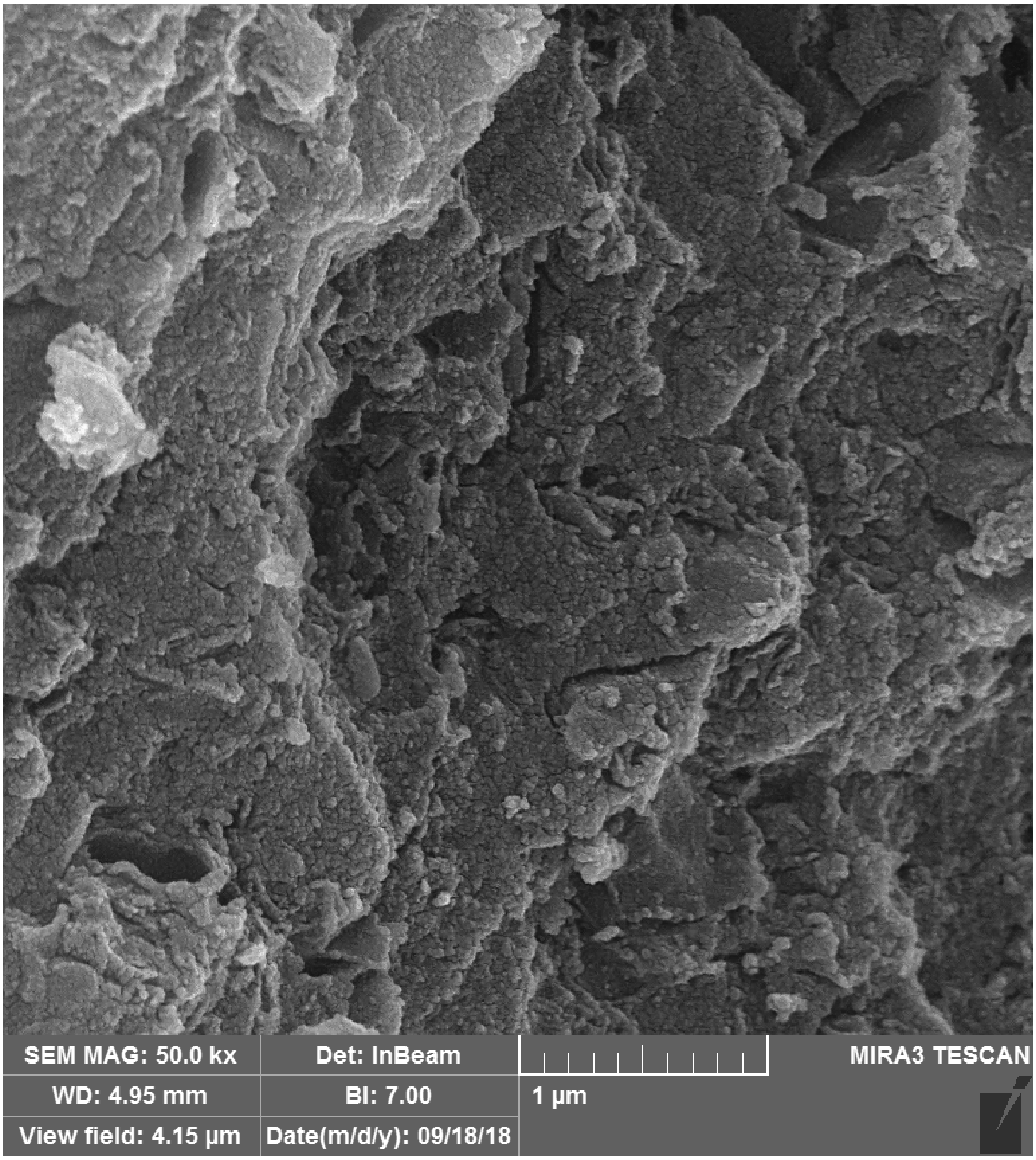

Figure S18. Typical FESEM image of the PPy/OMC (1 μm)

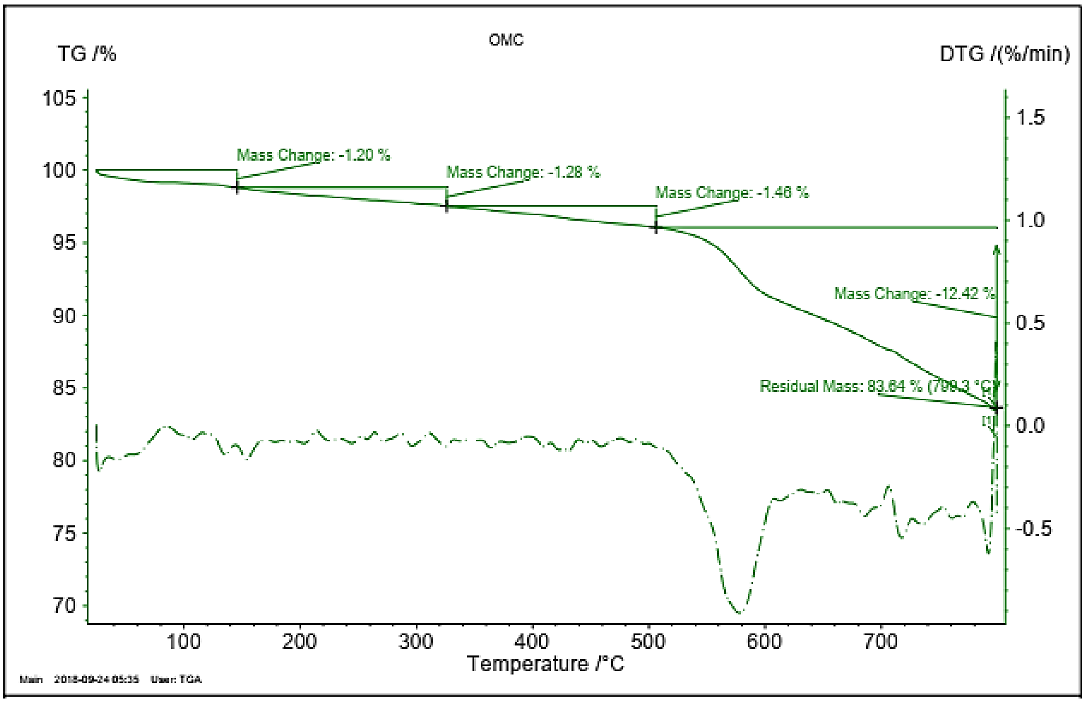

Figure S19. Thermogravimetric analysis curve of the OMC material under the nitrogen atmosphere

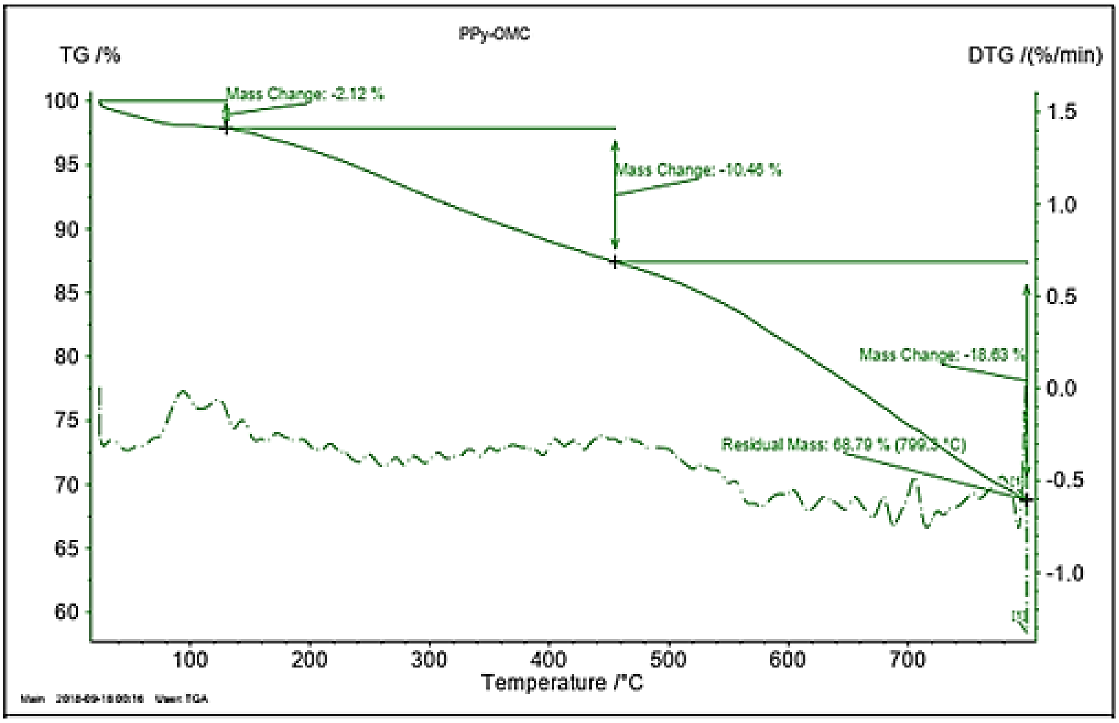

Figure S20. Thermogravimetric analysis curve of the PPy/OMC material under the nitrogen atmosphere

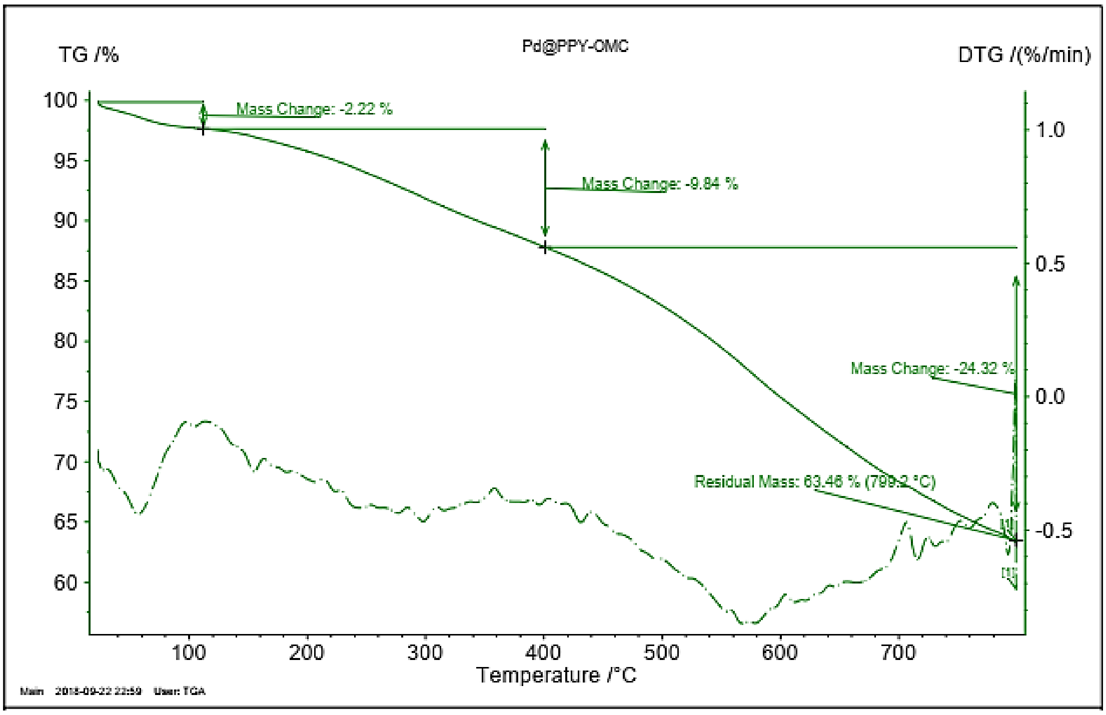

Figure S21. Thermogravimetric analysis curve of the Pd@PPy/OMC material under the nitrogen atmosphere

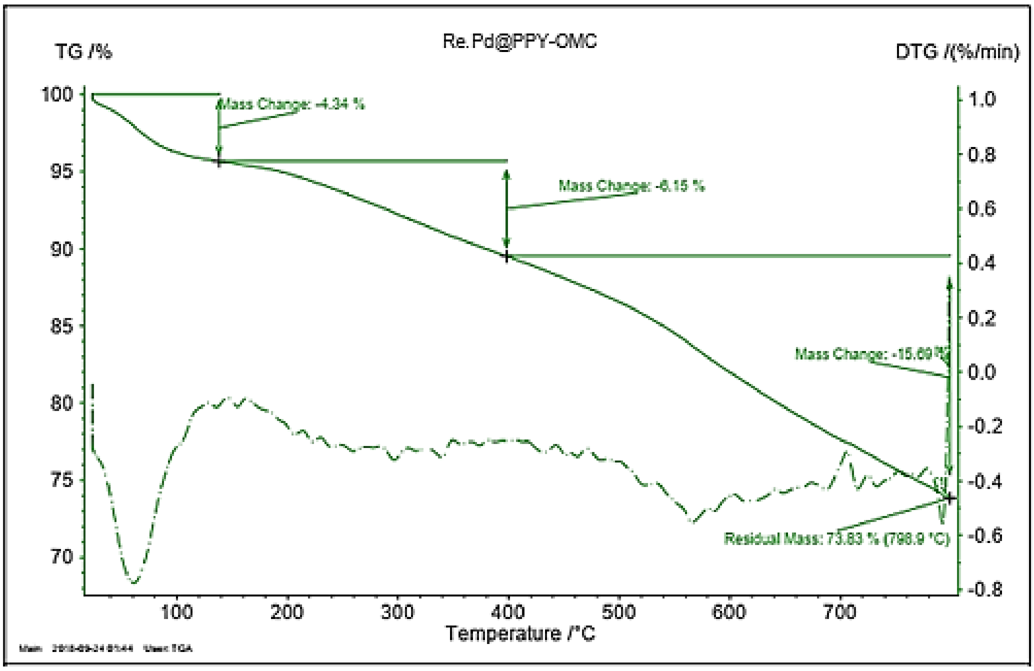

Figure S22. Thermogravimetric analysis curve of the recovered Pd@PPy/OMC material under the nitrogen atmosphere

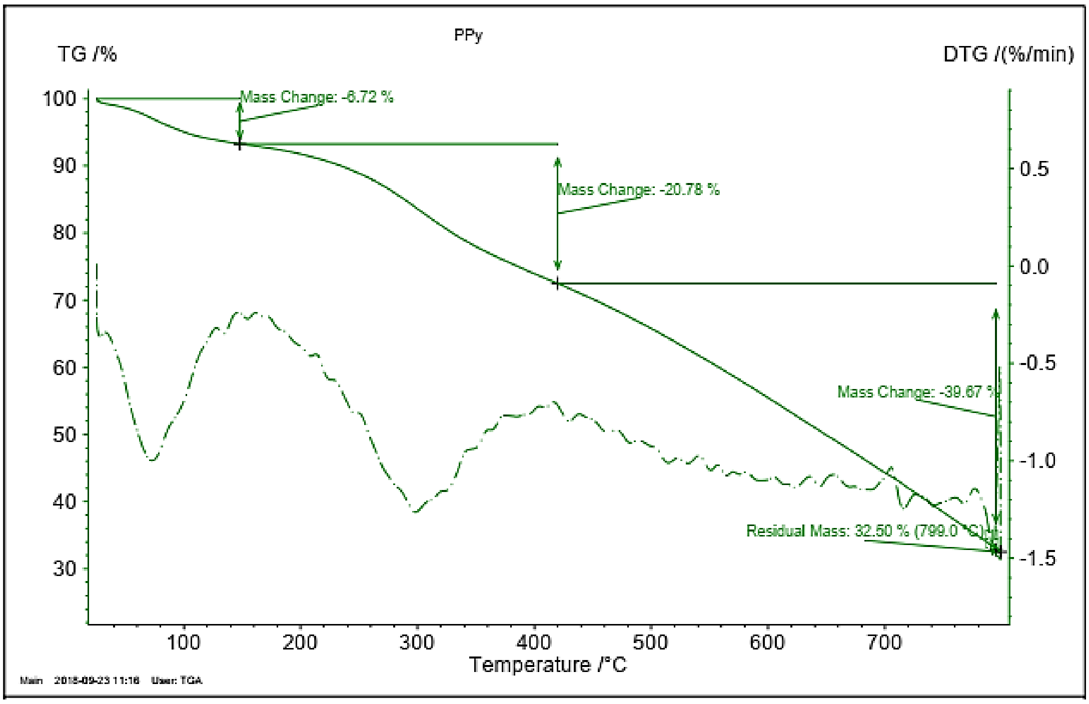

Figure S23. Thermogravimetric analysis curve of PPy under the nitrogen atmosphere

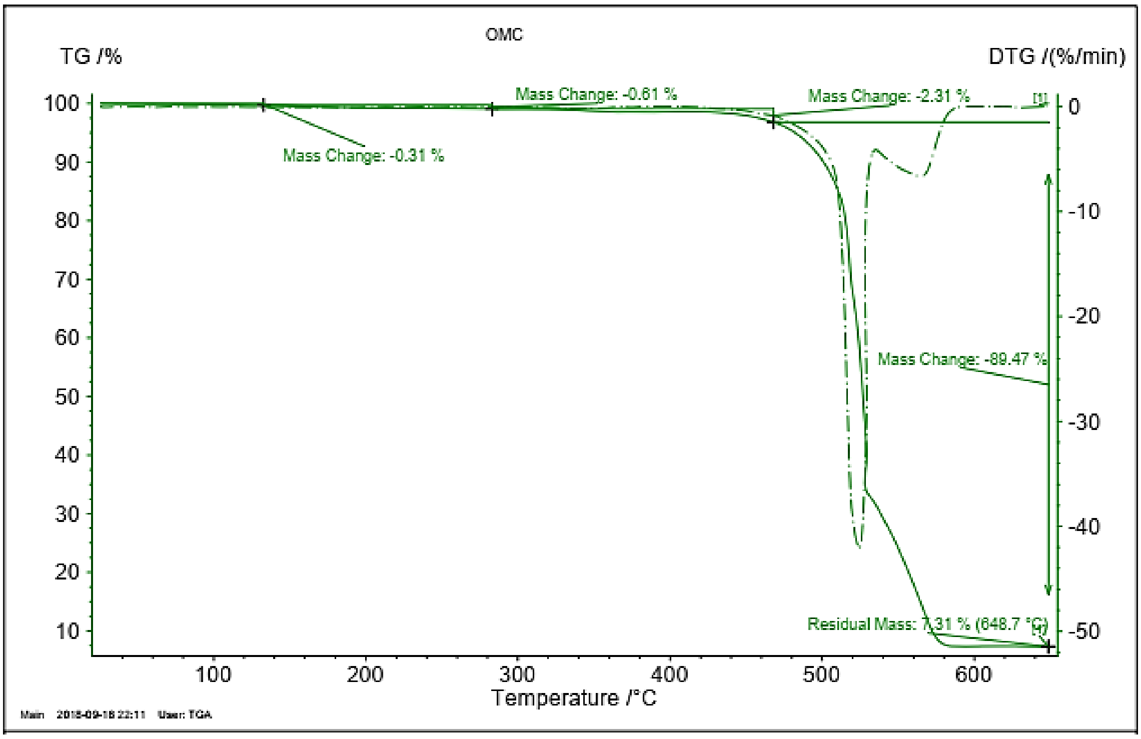

Figure S24. Thermogravimetric analysis curve of the OMC material under the oxygen atmosphere

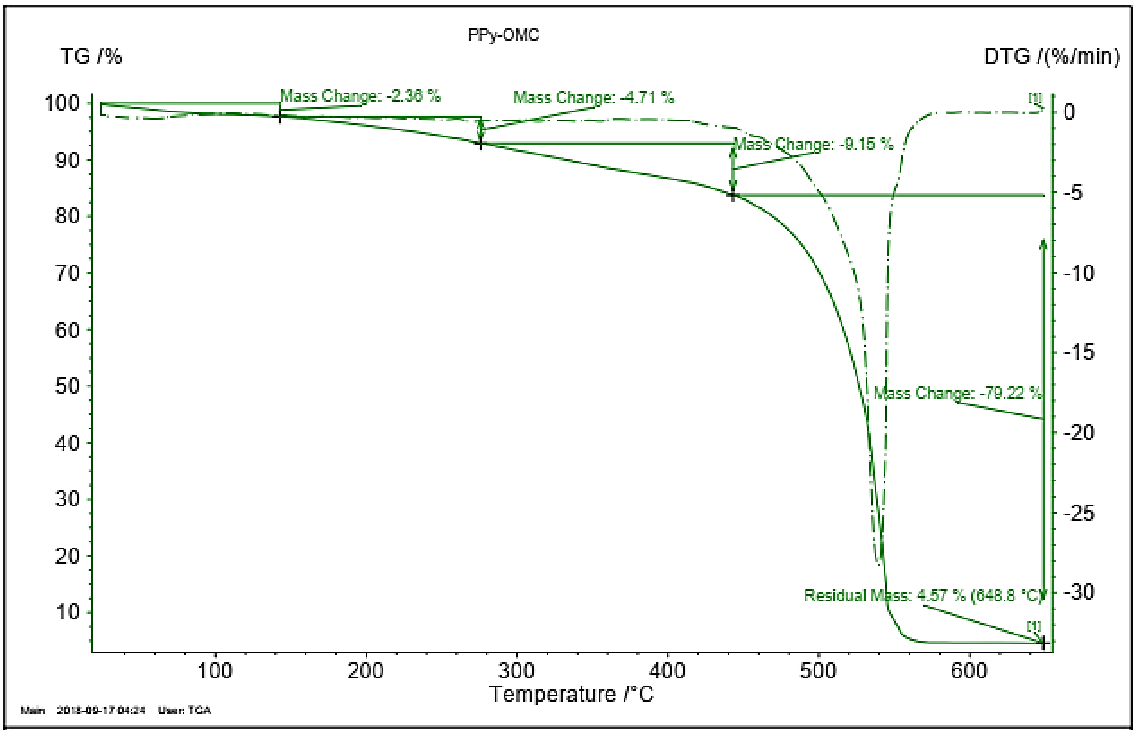

Figure S25. Thermogravimetric analysis curve of the PPy/OMC material under the oxygen atmosphere

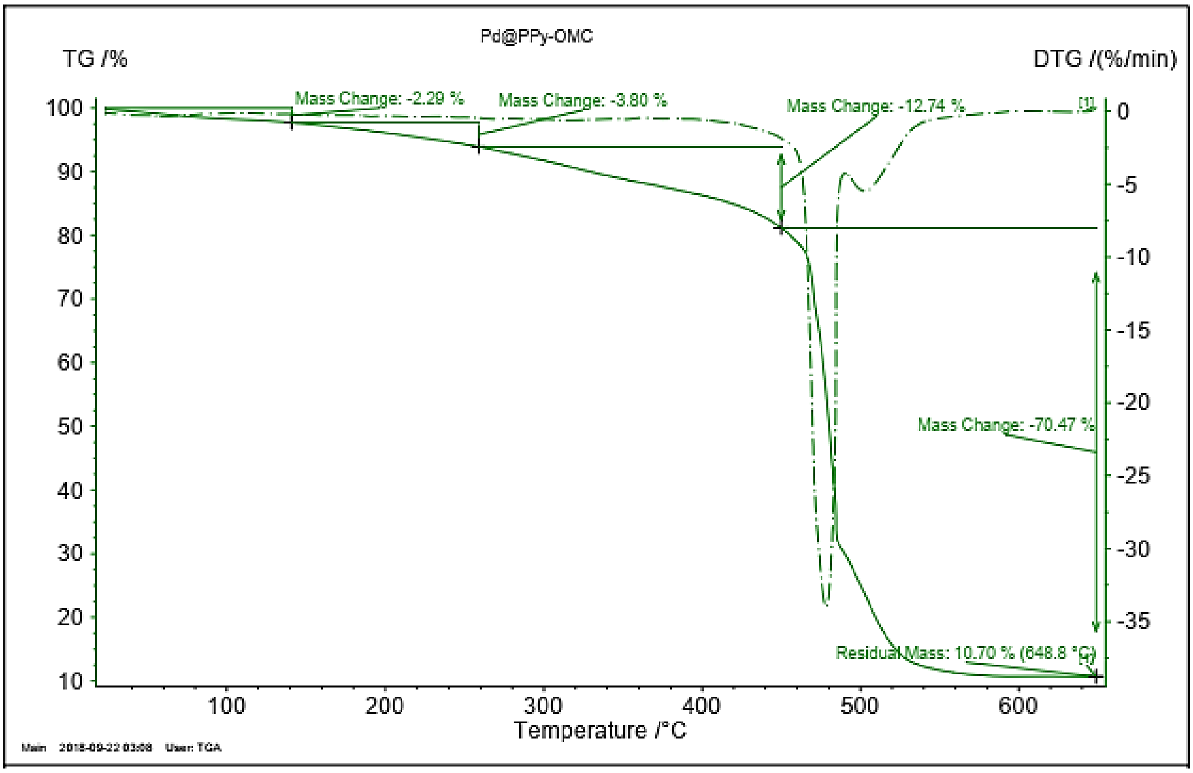

Figure S26. Thermogravimetric analysis curve of the Pd@PPy/OMC material under the oxygen atmosphere

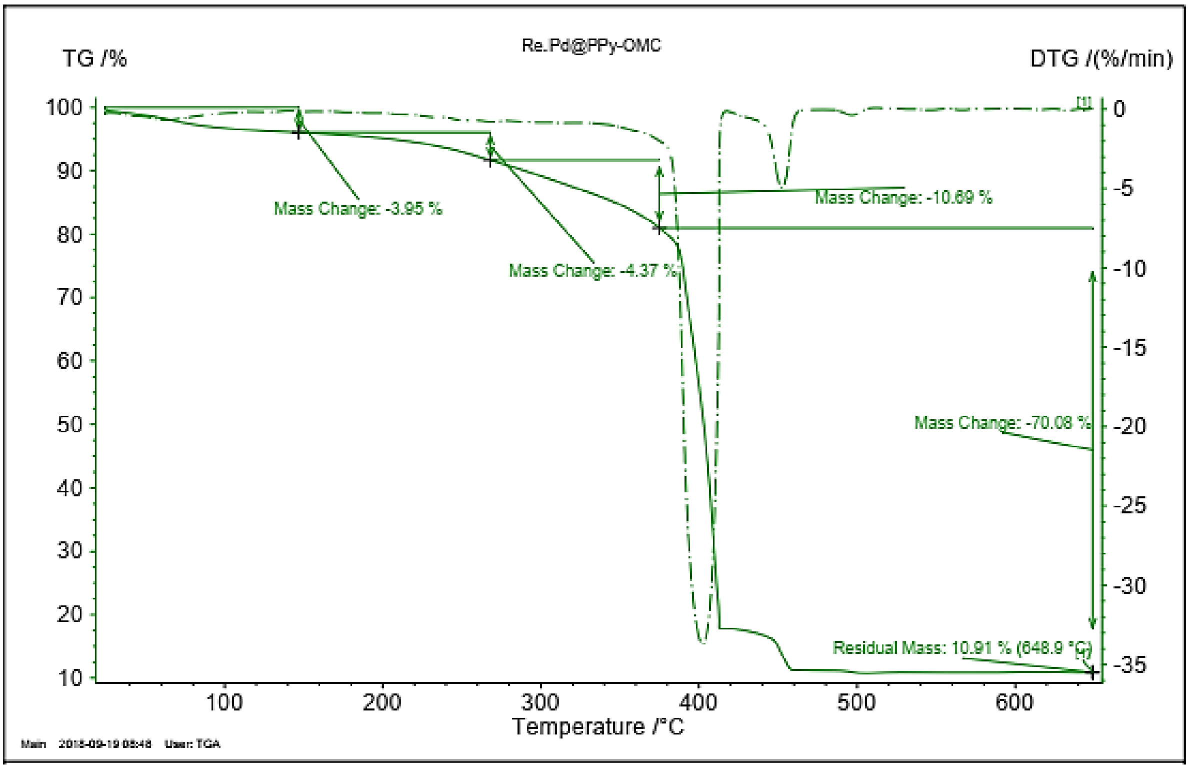

Figure S27. Thermogravimetric analysis curve of the recovered Pd@PPy/OMC material under the oxygen atmosphere

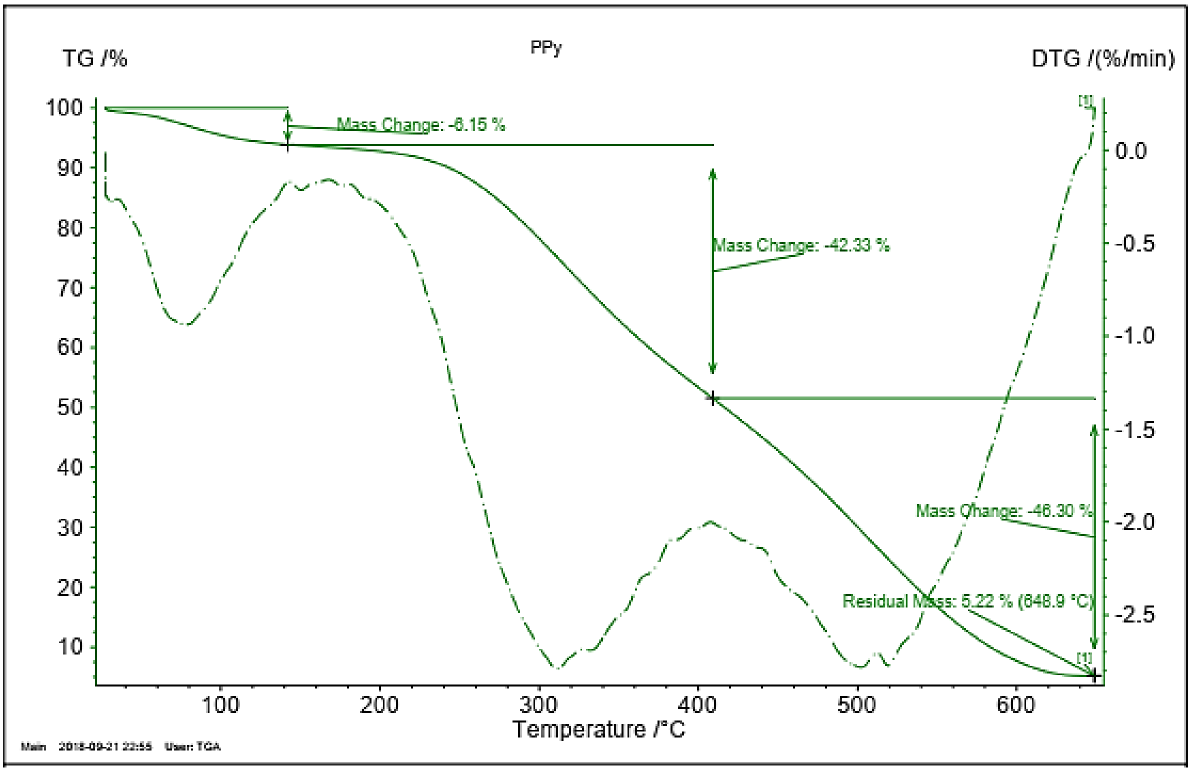

Figure S28. Thermogravimetric analysis curve of the recovered Pd@PPy/OMC material under the oxygen atmosphere

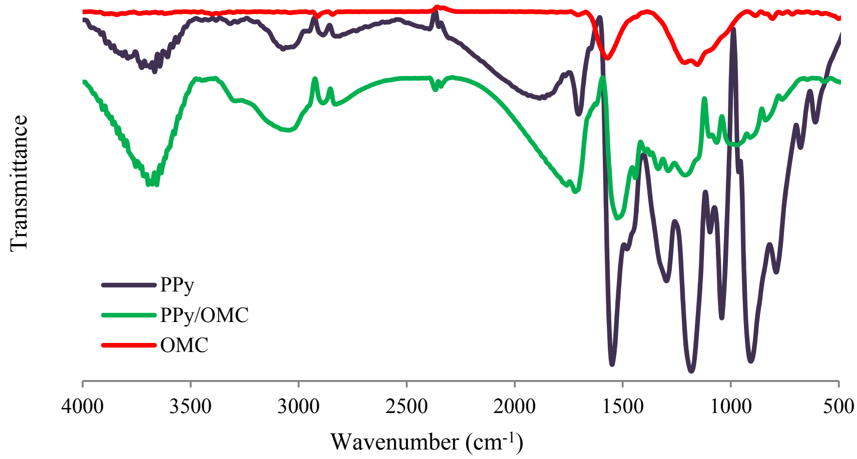

Figure S29. Infrared spectra of PPy, OMC and PPy/OMC powders

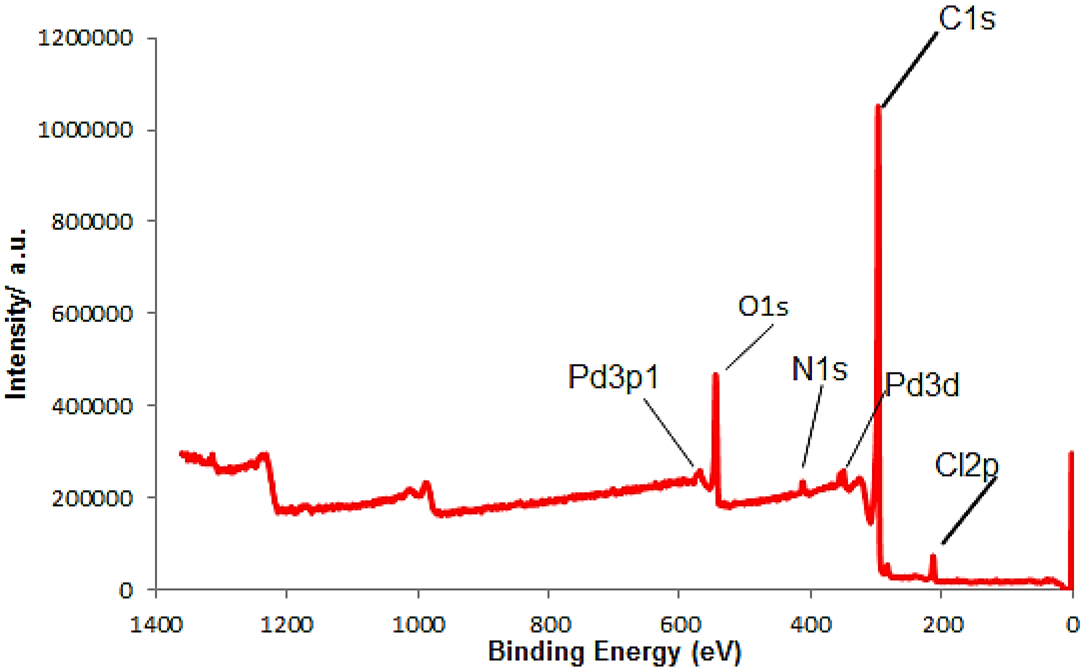

Figure S30. XPS survey spectrum of the Pd@PPy/OMC

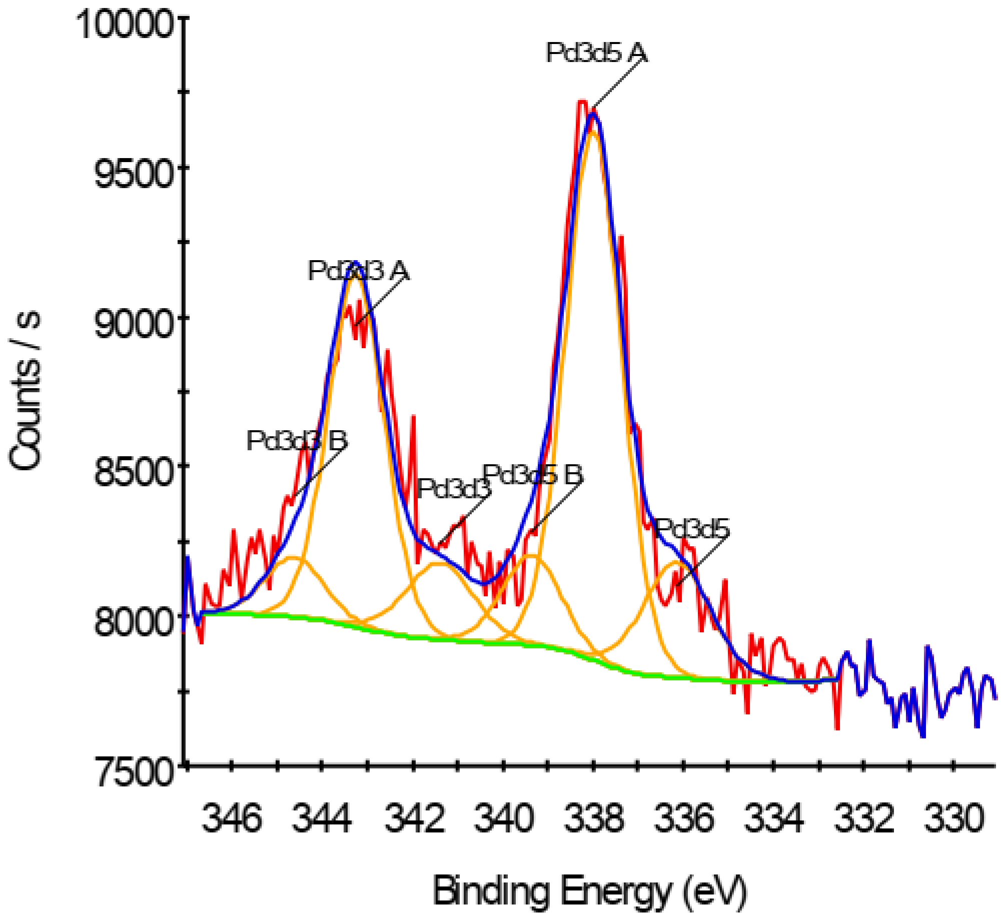

Figure S31. High-resolution XPS spectrum of Palladium

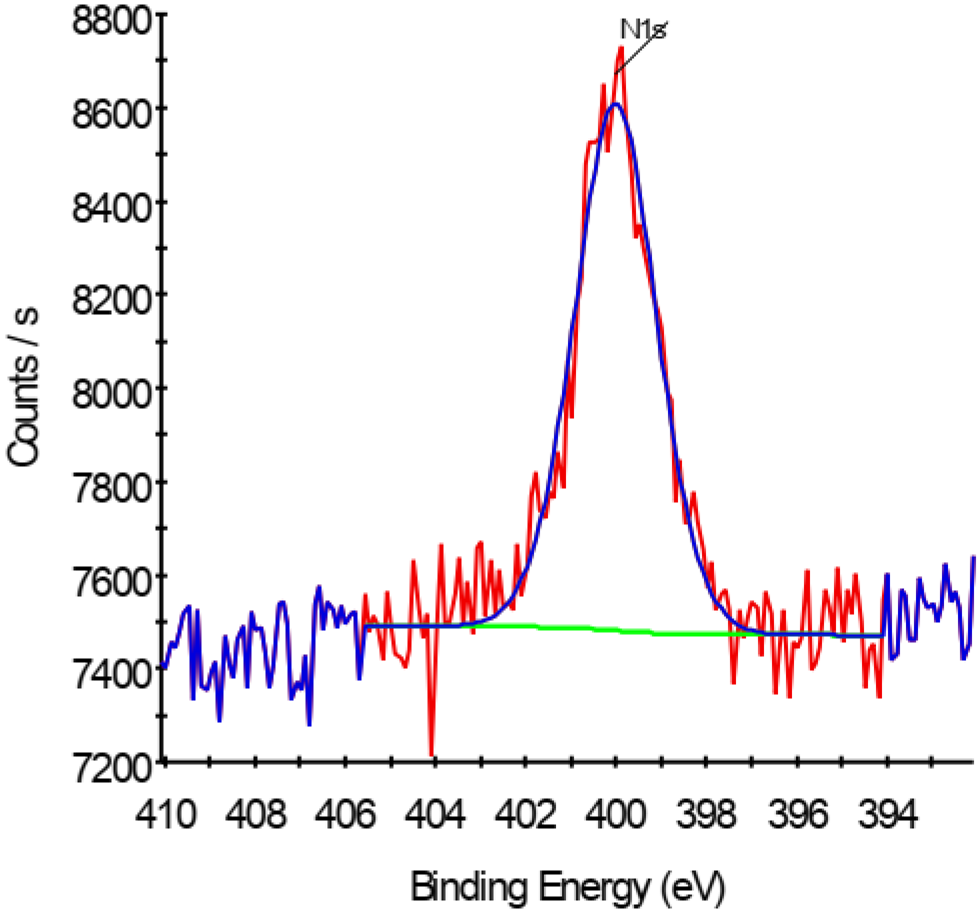

Figure S32. High-resolution XPS spectrum of nitrogen

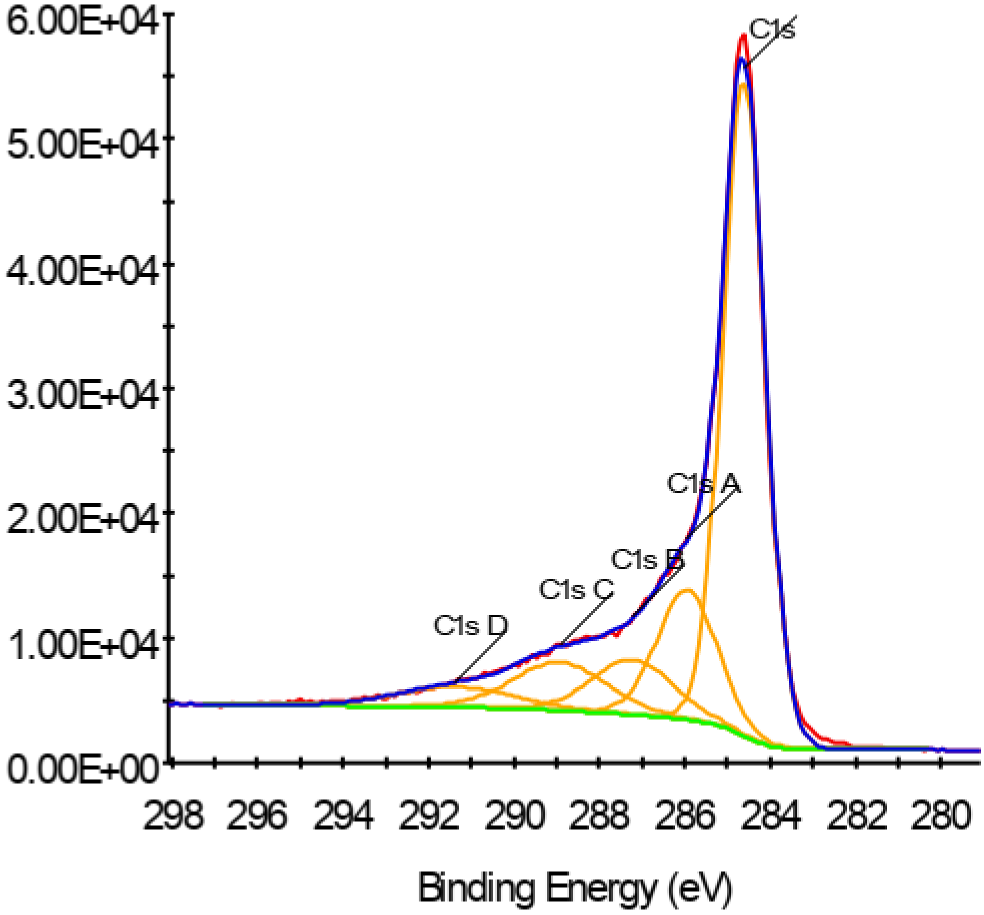

Figure S33. High-resolution XPS spectrum of carbon

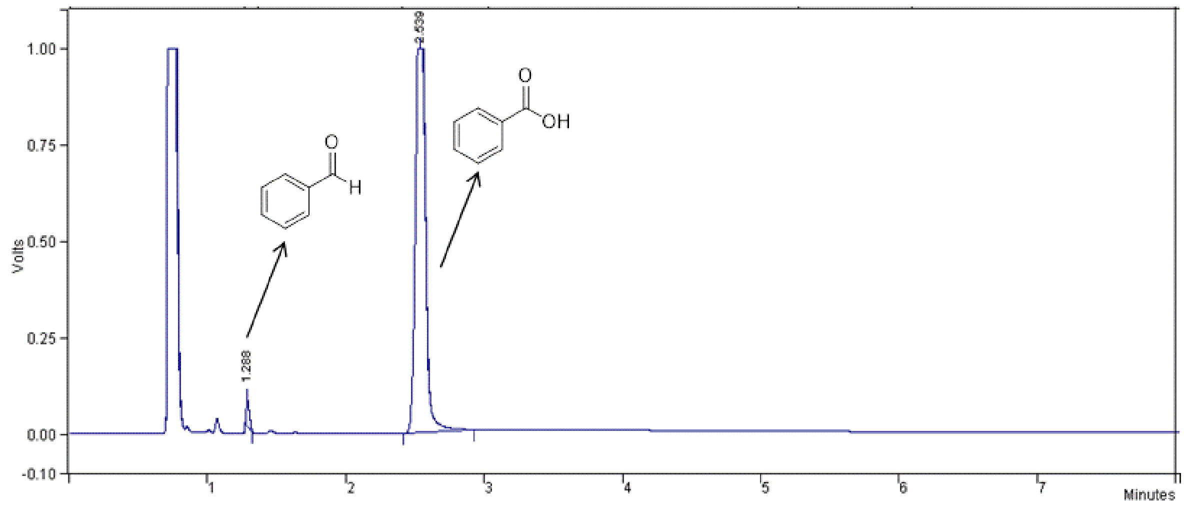

Peak No.	Peak Name	Result ()	Ret. Time (min)	Time Offset (min)	Area (counts)	Sep. Code	Width 1/2 (sec)	Status Codes
1		1.749	1.288	0.000	94168	BB	1.3	
2		98.251	2.539	0.000	5290184	BB	4.7	
	Totals:	100.000		0.000	5384352			

Figure S34. Gas chromatogram of benzyl alcohol (Table 3 Entry 1)

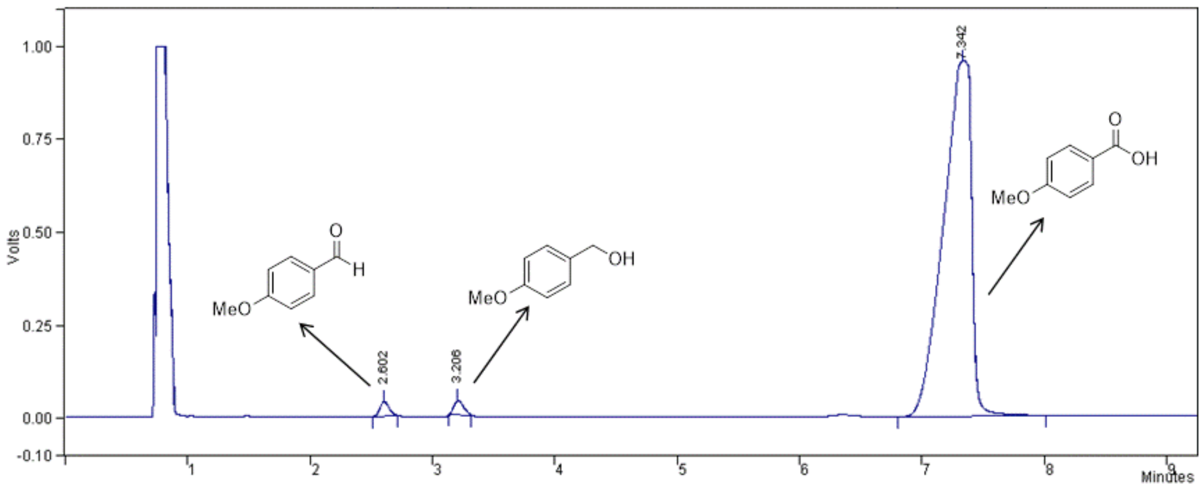

Peak No.	Peak Name	Result ()	Ret. Time (min)	Time Offset (min)	Area (counts)	Sep. Code	Width 1/2 (sec)	Status Codes
1		1.268	2.602	0.000	200597	BB	4.9	
2		1.362	3.206	0.000	215423	BB	5.3	
3		97.370	7.342	0.000	15402374	BB	14.4	
	Totals:	100.000		0.000	15818394			

Figure S35. Gas chromatogram of 4-methoxybenzyl alcohol (Table 3 Entry 2)

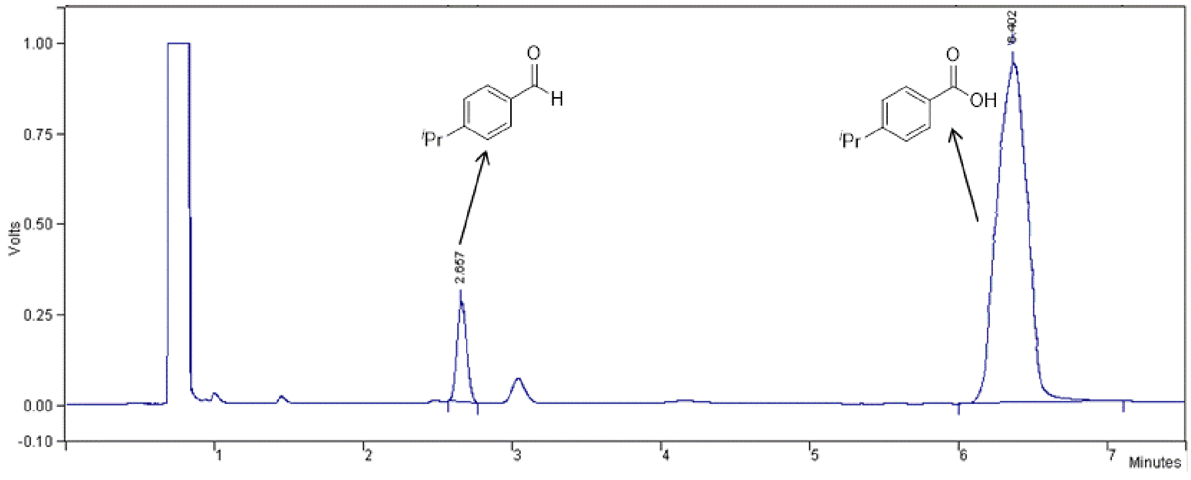

Peak No.	Peak Name	Result ()	Ret. Time (min)	Time Offset (min)	Area (counts)	Sep. Code	Width 1/2 (sec)	Status Codes
1		8.077	2.657	0.000	1258899	BB	4.3	
2		91.923	6.402	0.000	14327840	BB	13.2	
	Totals:	100.000		0.000	15586739			

Figure S36. Gas chromatogram of 4-isopropylbenzyl alcohol (Table 3 Entry 3)

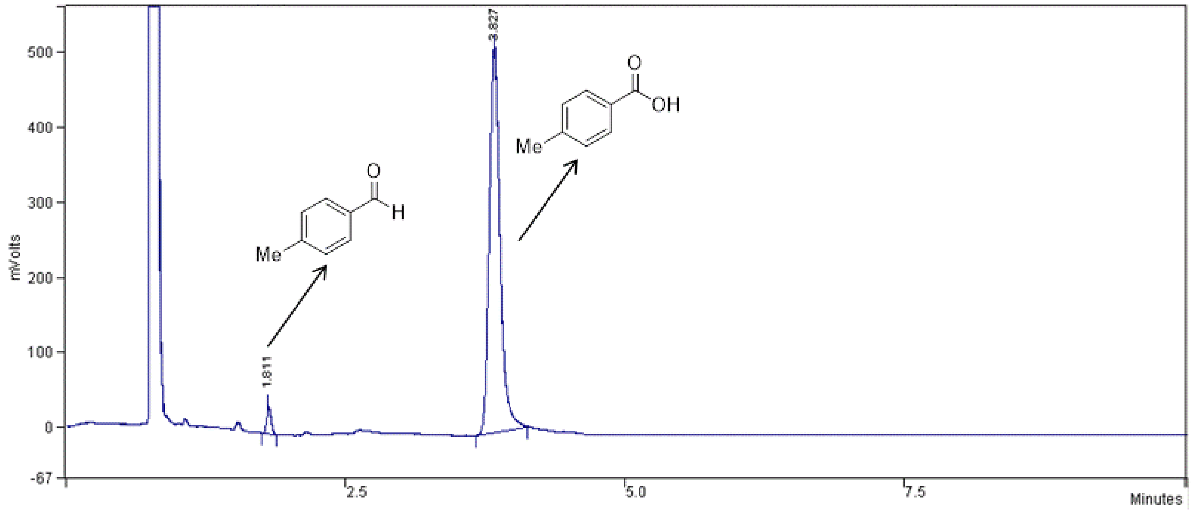

Peak No.	Peak Name	Result ()	Ret. Time (min)	Time Offset (min)	Area (counts)	Sep. Code	Width 1/2 (sec)	Status Codes
1		2.815	1.811	0.000	98647	BB	2.5	
2		97.185	3.827	0.000	3405828	BB	5.9	
	Totals:	100.000		0.000	3504475			

Figure S37. Gas chromatogram of 4-methylbenzyl alcohol (Table 3 Entry 4)

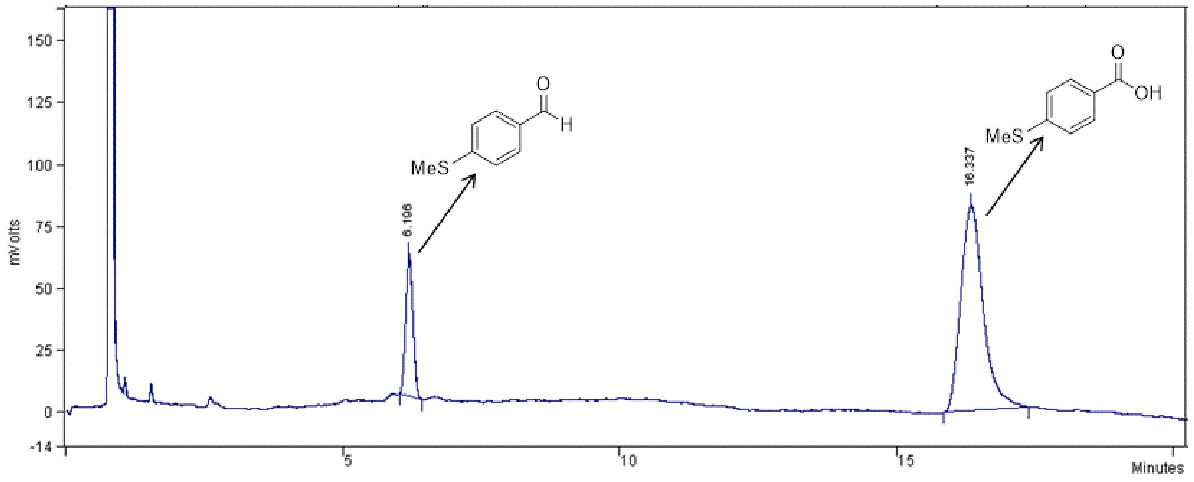

Peak No.	Peak Name	Result ()	Ret. Time (min)	Time Offset (min)	Area (counts)	Sep. Code	Width 1/2 (sec)	Status Codes
1		18.315	6.196	0.000	528175	BB	8.6	
2		81.685	16.337	0.000	2355737	BB	25.5	
	Totals:	100.000		0.000	2883912			

Figure S38. Gas chromatogram of 4-(methylthio)benzyl alcohol (Table 3 Entry 5)

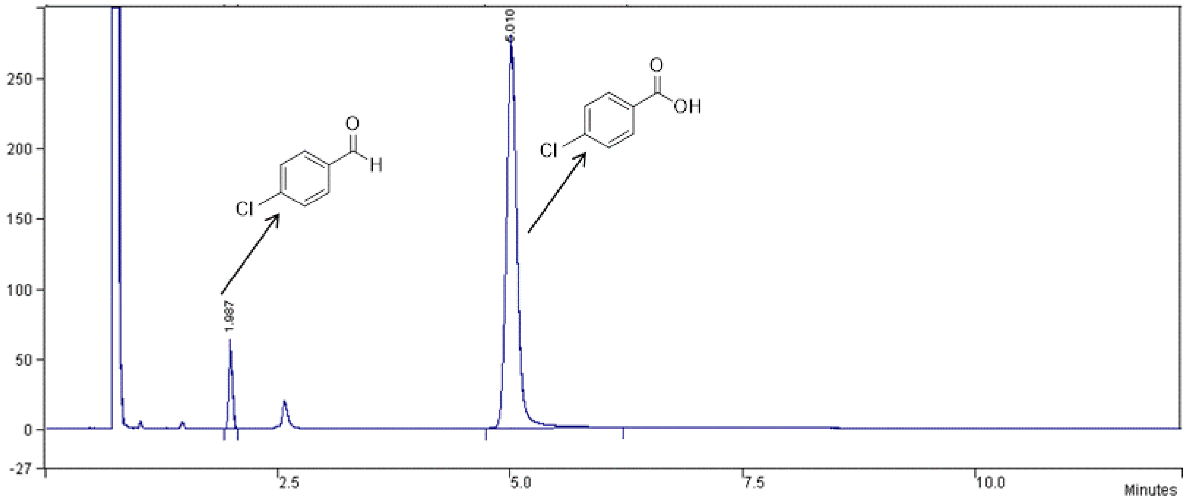

Peak No.	Peak Name	Result ()	Ret. Time (min)	Time Offset (min)	Area (counts)	Sep. Code	Width 1/2 (sec)	Status Codes
1		6.541	1.987	0.000	154122	BB	2.6	
2		93.459	5.010	0.000	2202204	BB	7.1	
	Totals:	100.000		0.000	2356326			

Figure S39. Gas chromatogram of 4-chlorobenzyl alcohol (Table 3 Entry 6)

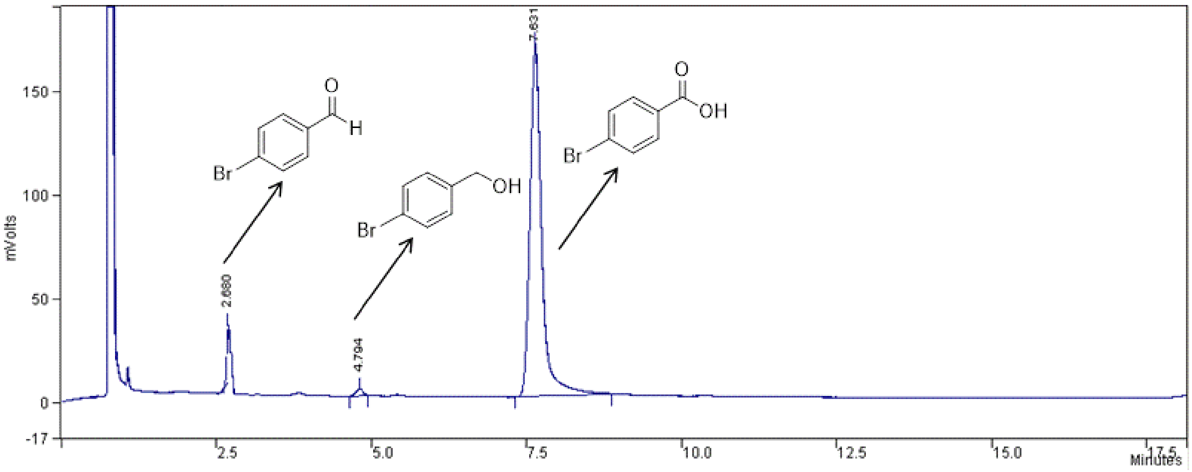

Peak No.	Peak Name	Result ()	Ret. Time (min)	Time Offset (min)	Area (counts)	Sep. Code	Width 1/2 (sec)	Status Codes
1		4.063	2.680	0.000	98395	BB	3.8	
2		1.040	4.794	0.000	25182	BB	7.4	
3		94.897	7.631	0.000	2298270	BB	11.4	
	Totals:	100.000		0.000	2421847			

Figure S40. Gas chromatogram of 4-bromobenzyl alcohol (Table 3 Entry 7)

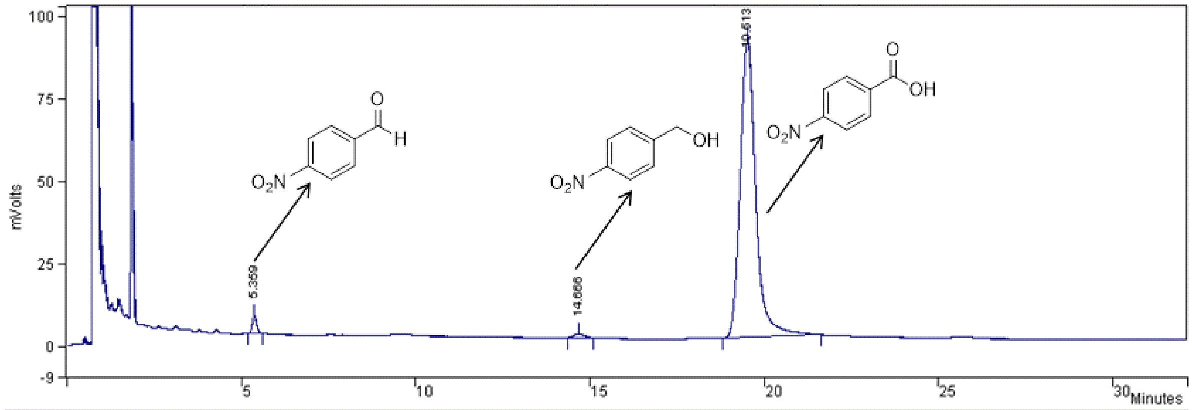

Peak No.	Peak Name	Result ()	Ret. Time (min)	Time Offset (min)	Area (counts)	Sep. Code	Width 1/2 (sec)	Status Codes
1		1.646	5.359	0.000	47755	BB	8.1	
2		0.855	14.666	0.000	24808	BB	21.1	
3		97.499	19.513	0.000	2829073	BB	26.9	
	Totals:	100.000		0.000	2901636			

Figure S41. Gas chromatogram of 4-nitrobenzyl alcohol (Table 3 Entry 8)

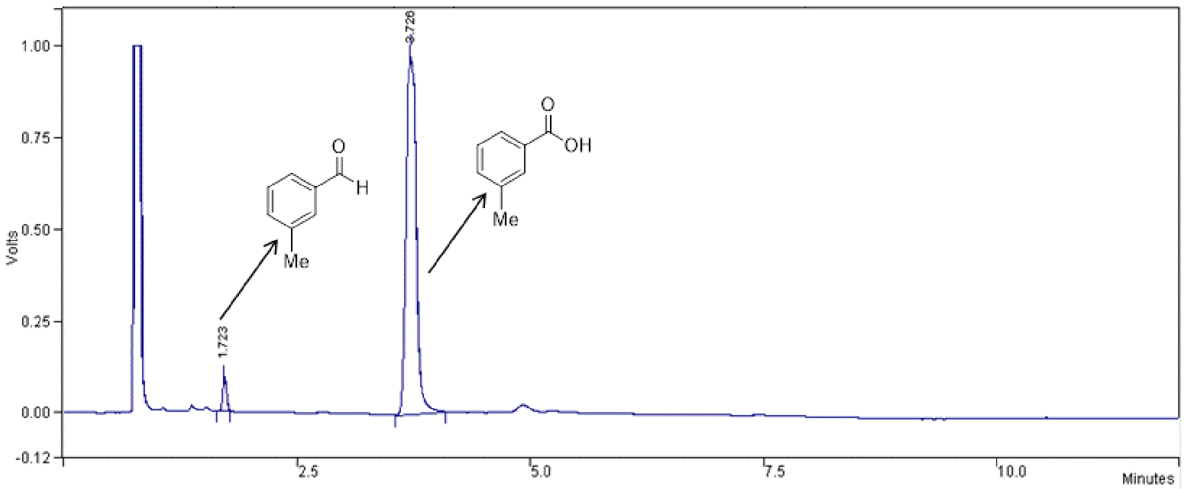

Peak No.	Peak Name	Result ()	Ret. Time (min)	Time Offset (min)	Area (counts)	Sep. Code	Width 1/2 (sec)	Status Codes
1		3.269	1.723	0.000	261389	BB	2.6	
2		96.731	3.726	0.000	7734212	BB	6.9	
	Totals:	100.000		0.000	7995601			

Figure S42. Gas chromatogram of 3-methylbenzyl alcohol (Table 3 Entry 9)

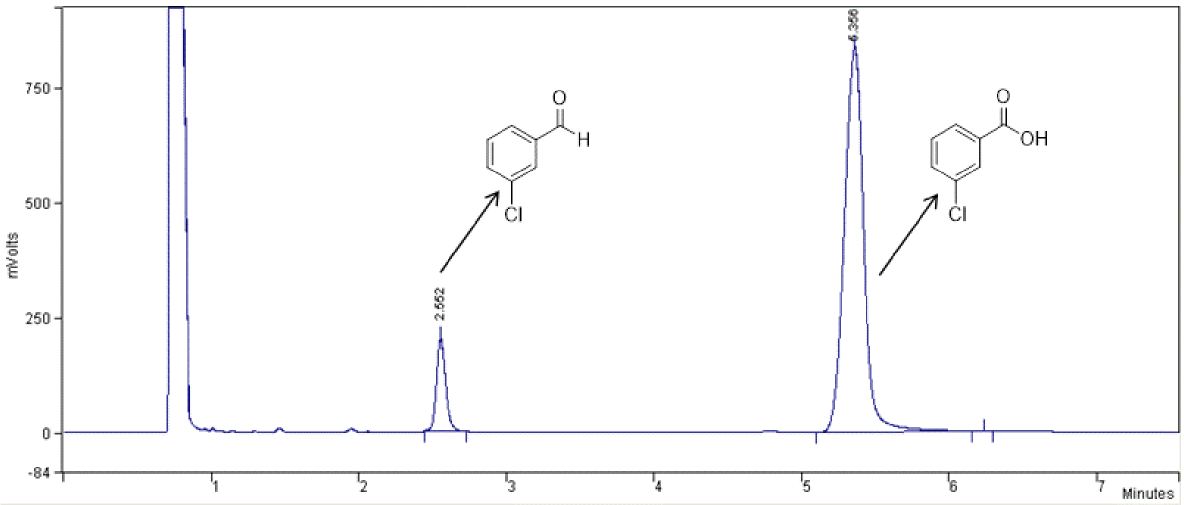

Peak No.	Peak Name	Result ()	Ret. Time (min)	Time Offset (min)	Area (counts)	Sep. Code	Width 1/2 (sec)	Status Codes
1		10.148	2.552	0.000	852884	BB	3.8	
2		89.852	5.356	0.000	7551902	BB	8.1	
	Totals:	100.000		0.000	8404786			

Figure S 43. Gas chromatogram of 3-chlorobenzyl alcohol (Table 3 Entry 10)

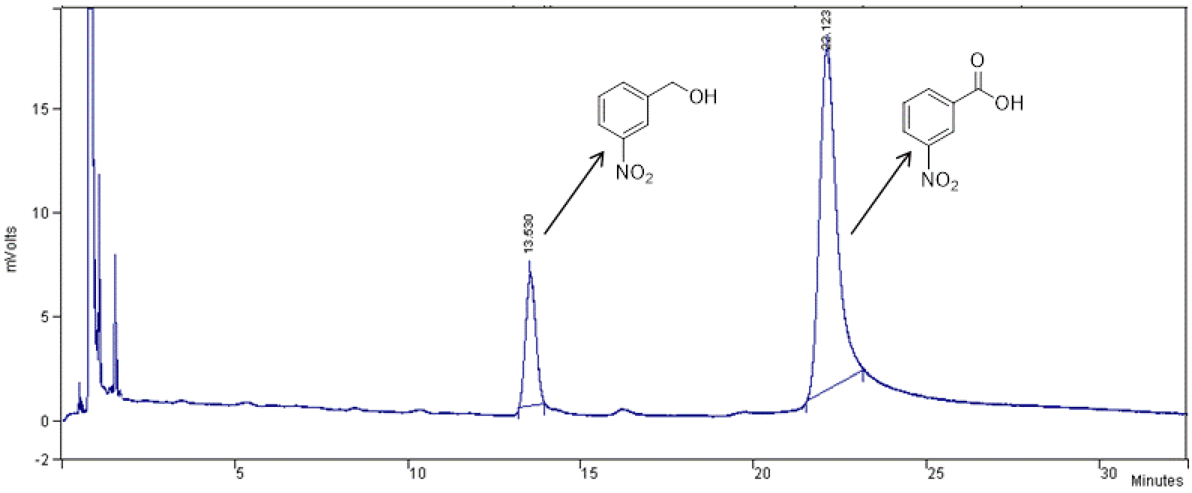

Peak No.	Peak Name	Result ()	Ret. Time (min)	Time Offset (min)	Area (counts)	Sep. Code	Width 1/2 (sec)	Status Codes
1		18.533	13.530	0.000	130068	BB	19.6	
2		81.467	22.123	0.000	571762	BB	32.2	
	Totals:	100.000		0.000	701830			

Figure S44. Gas chromatogram of 3-nitrobenzyl alcohol (Table 3 Entry 11)

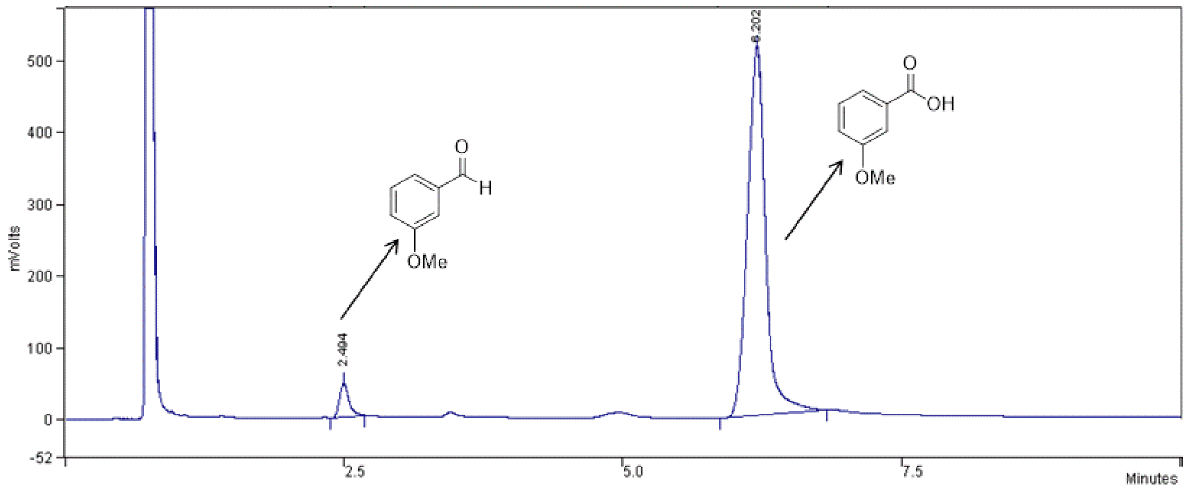

Peak No.	Peak Name	Result ()	Ret. Time (min)	Time Offset (min)	Area (counts)	Sep. Code	Width 1/2 (sec)	Status Codes
1		4.574	2.494	0.000	279308	BB	5.5	
2		95.426	6.202	0.000	5826816	BB	9.9	
	Totals:	100.000		0.000	6106124			

Figure S45. Gas chromatogram of 3-methoxybenzyl alcohol (Table 3 Entry 12)

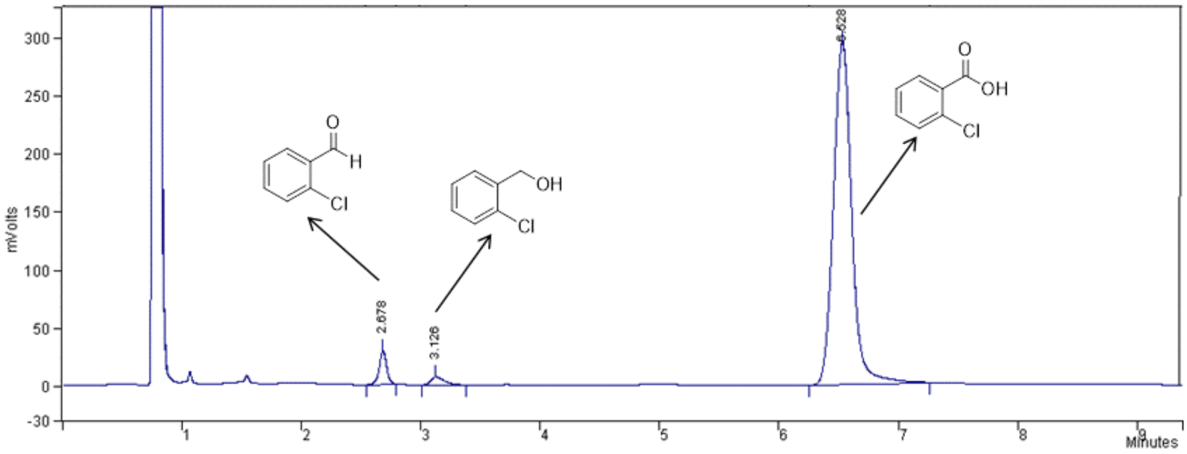

Peak No.	Peak Name	Result ()	Ret. Time (min)	Time Offset (min)	Area (counts)	Sep. Code	Width 1/2 (sec)	Status Codes
1		3.802	2.678	0.000	125818	BB	3.8	
2		1.596	3.126	0.000	52827	BB	6.9	
3		94.602	6.528	0.000	3130732	BB	9.4	
	Totals:	100.000		0.000	3309377			

Figure S46. Gas chromatogram of 2-chlorobenzyl alcohol (Table 3 Entry 13)

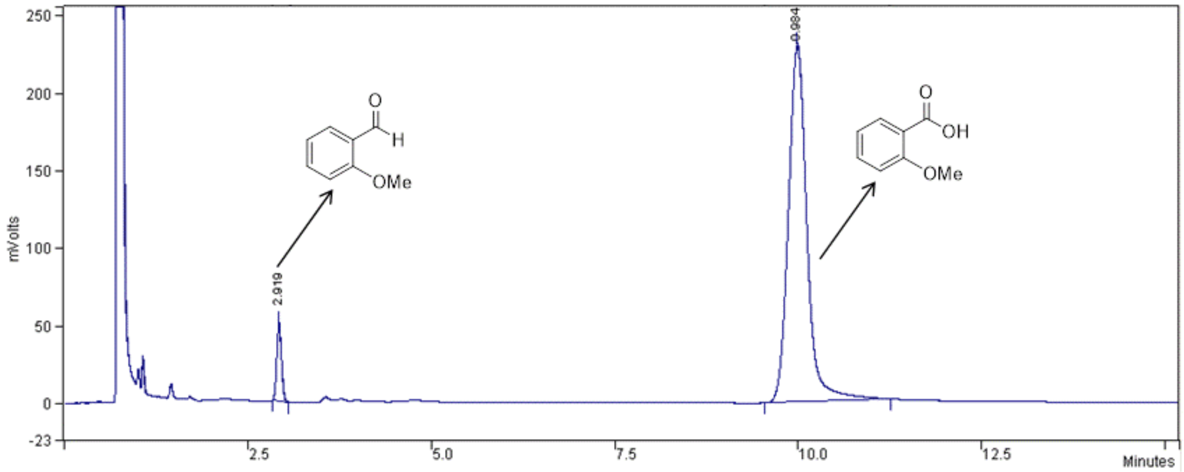

Peak No.	Peak Name	Result ()	Ret. Time (min)	Time Offset (min)	Area (counts)	Sep. Code	Width 1/2 (sec)	Status Codes
1		5.238	2.919	0.000	222934	BB	4.2	
2		94.762	9.984	0.000	4033510	BB	15.4	
	Totals:	100.000		0.000	4256444			

Figure S47. Gas chromatogram of 2-methoxybenzyl alcohol (Table 3 Entry 14)

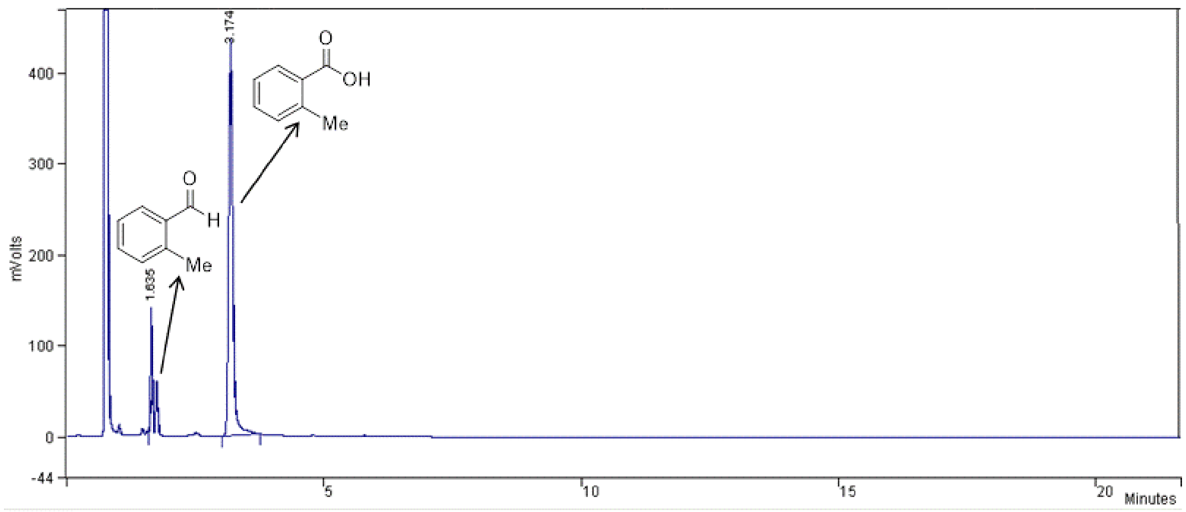

Peak No.	Peak Name	Result ()	Ret. Time (min)	Time Offset (min)	Area (counts)	Sep. Code	Width 1/2 (sec)	Status Codes
1		7.218	1.635	0.000	182927	BB	2.3	
2		92.782	3.174	0.000	2351355	BB	4.7	
	Totals:	100.000		0.000	2534282			

Figure S48. Gas chromatogram of 2-methylbenzyl alcohol (Table 3 Entry 15)

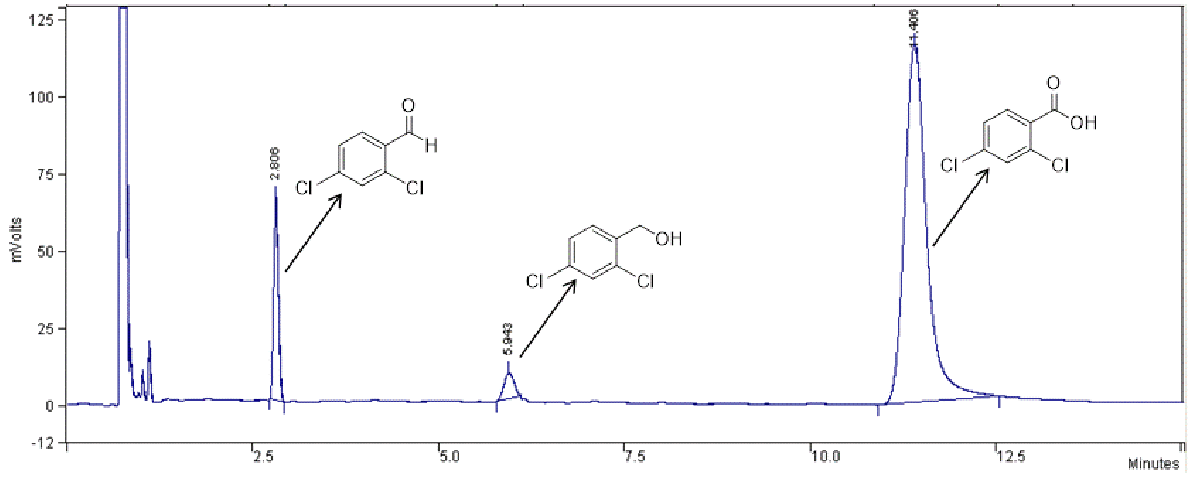

Peak No.	Peak Name	Result ()	Ret. Time (min)	Time Offset (min)	Area (counts)	Sep. Code	Width 1/2 (sec)	Status Codes
1		10.080	2.806	0.000	279596	BB	4.0	
2		2.830	5.943	0.000	78506	BB	9.8	
3		87.090	11.406	0.000	2415637	BB	18.1	
	Totals:	100.000		0.000	2773739			

Figure S49. Gas chromatogram of 2,4-dichlorobenzyl alcohol (Table 3 Entry 16)

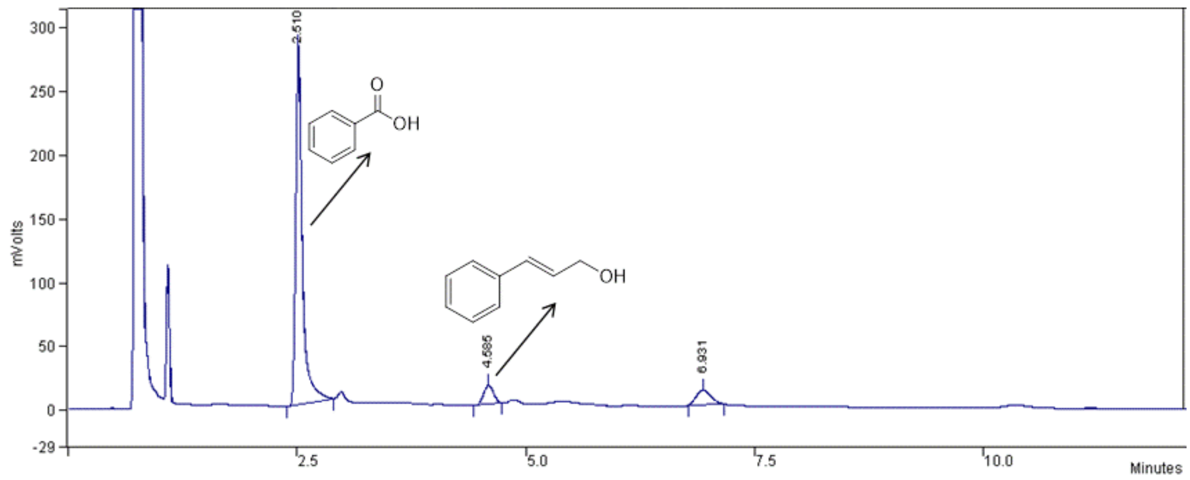

Peak No.	Peak Name	Result ()	Ret. Time (min)	Time Offset (min)	Area (counts)	Sep. Code	Width 1/2 (sec)	Status Codes
1		85.737	2.510	0.000	1440312	BB	4.1	
2		6.683	4.585	0.000	112263	BB	7.7	
3		7.580	6.931	0.000	127346	BB	10.9	
	Totals:	100.000		0.000	1679921			

Figure S50. Gas chromatogram of cinnamyl alcohol (Table 3 Entry 17)

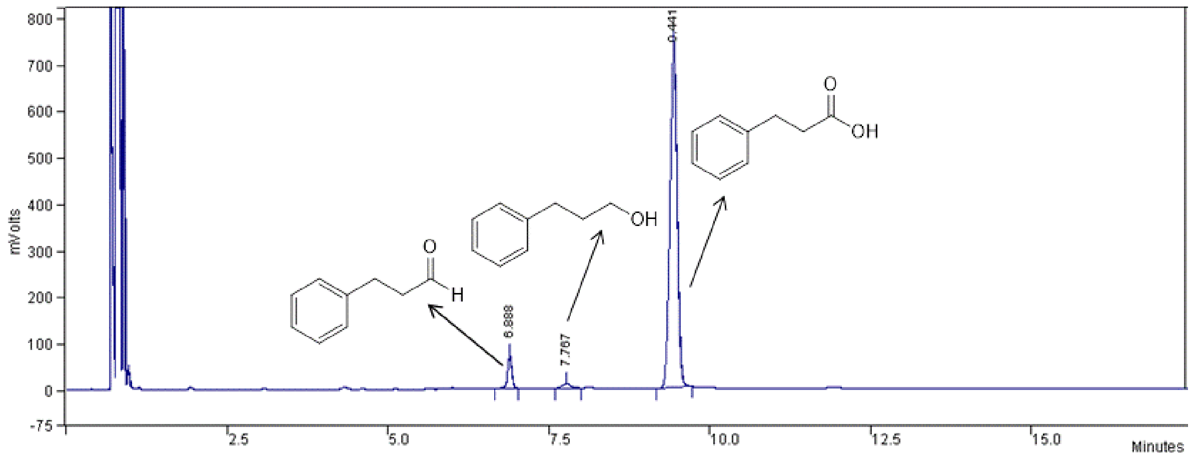

Peak No.	Peak Name	Result ()	Ret. Time (min)	Time Offset (min)	Area (counts)	Sep. Code	Width 1/2 (sec)	Status Codes
1		5.152	6.888	0.000	319906	BB	4.0	
2		1.479	7.767	0.000	91822	BB	8.5	
3		93.369	9.441	0.000	5797621	BB	7.3	
	Totals:	100.000		0.000	6209349			

Figure S51. Gas chromatogram of 3-phenyl-1-propanol (Table 3 Entry 18)

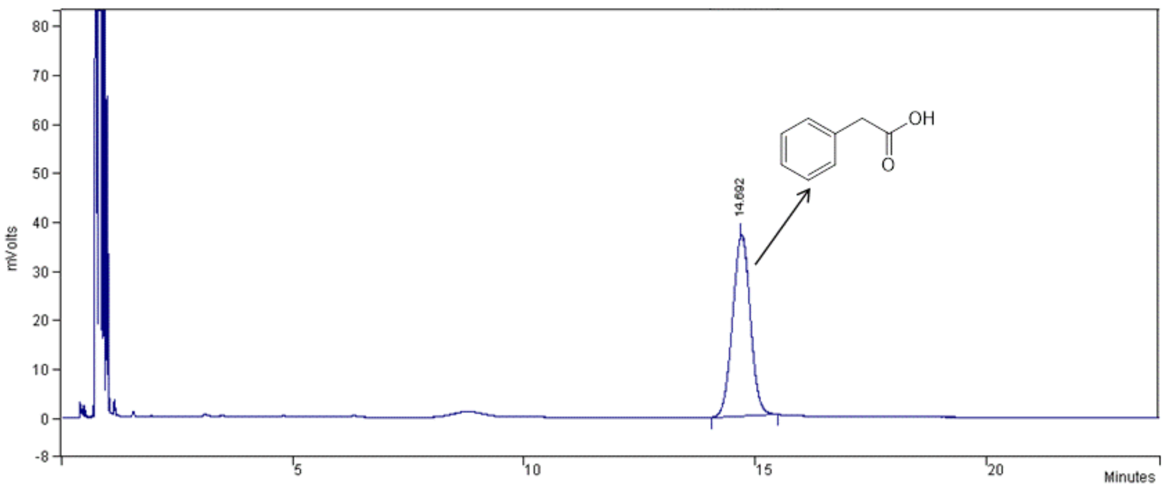

Peak No.	Peak Name	Result ()	Ret. Time (min)	Time Offset (min)	Area (counts)	Sep. Code	Width 1/2 (sec)	Status Codes
1		100.000	14.692	0.000	985746	BB	24.8	
	Totals:	100.000		0.000	985746			

Figure S52. Gas chromatogram of 2-phenyl-1-ethanol (Table 3 Entry 19)

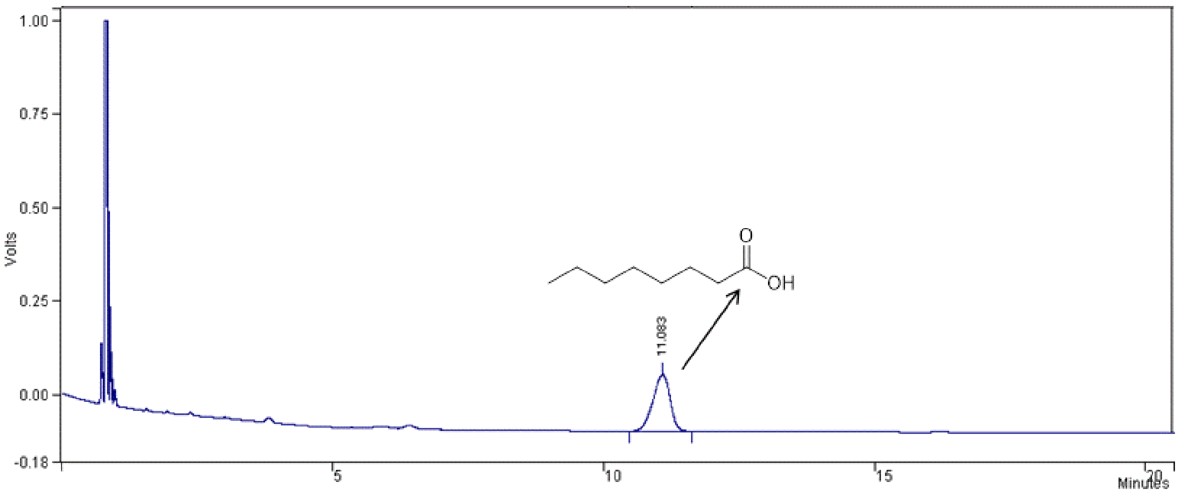

Peak No.	Peak Name	Result ()	Ret. Time (min)	Time Offset (min)	Area (counts)	Sep. Code	Width 1/2 (sec)	Status Codes
1		100.000	11.083	0.000	3348473	BB	20.6	
	Totals:	100.000		0.000	3348473			

Figure S53. Gas chromatogram of 1-octanol (Table 3 Entry 20)

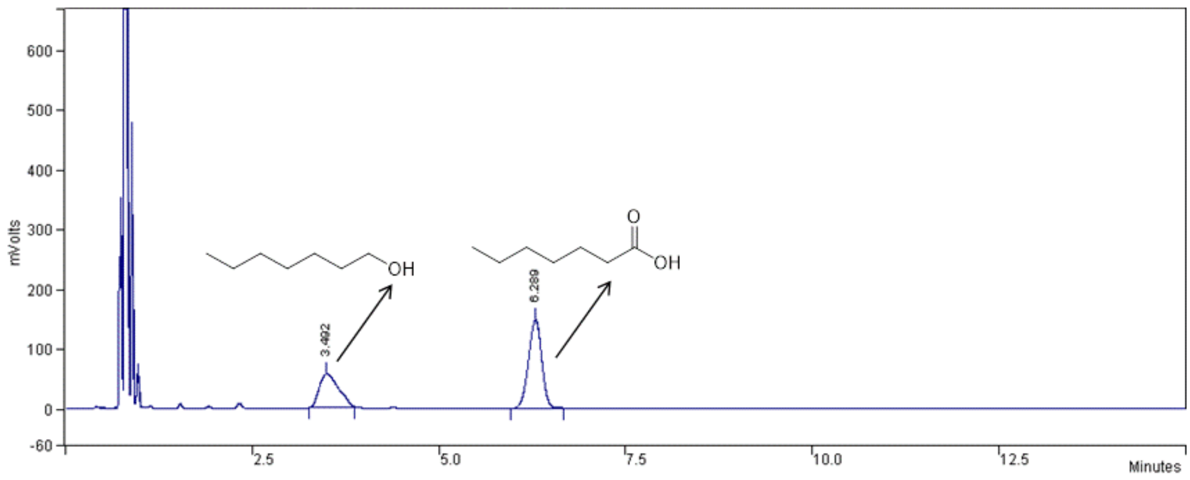

Peak No.	Peak Name	Result ()	Ret. Time (min)	Time Offset (min)	Area (counts)	Sep. Code	Width 1/2 (sec)	Status Codes
1		35.344	3.492	0.000	997097	BB	17.8	
2		64.656	6.289	0.000	1824015	BB	11.5	
	Totals:	100.000		0.000	2821112			

Figure S54. Gas chromatogram of 1-heptanol (Table 3 Entry 21)

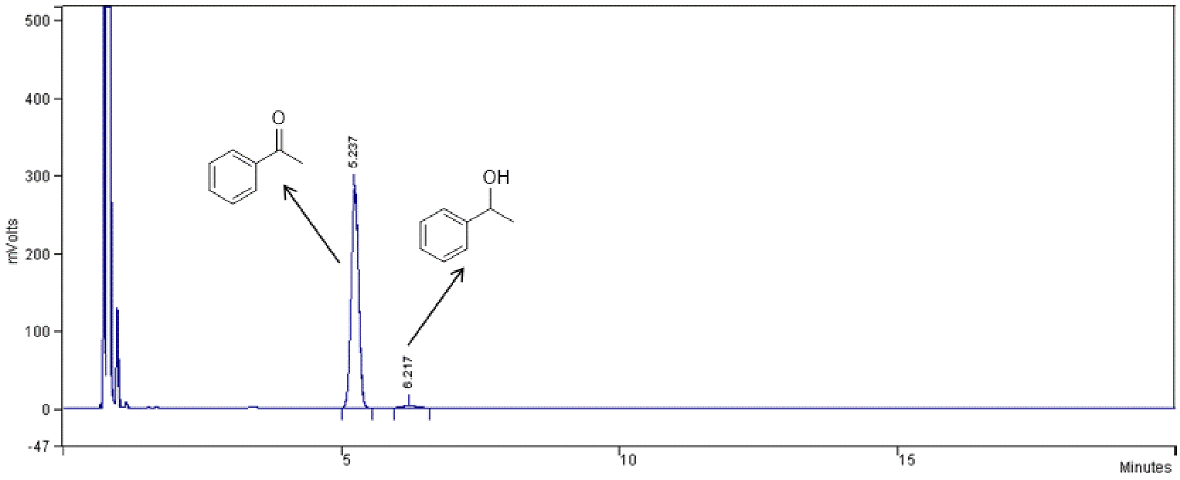

Peak No.	Peak Name	Result ()	Ret. Time (min)	Time Offset (min)	Area (counts)	Sep. Code	Width 1/2 (sec)	Status Codes
1		98.346	5.237	0.000	2633156	BB	8.6	
2		1.654	6.217	0.000	44280	BB	16.4	
	Totals:	100.000		0.000	2677436			

Figure S55. Gas chromatogram of 1-phenyl-1-ethanol (Table 3 Entry 22)

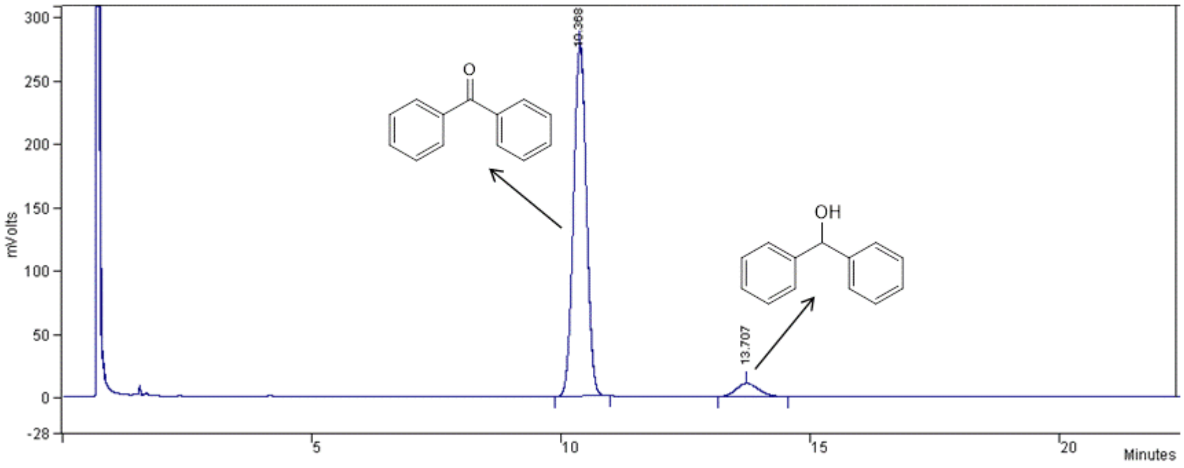

Peak No.	Peak Name	Result ()	Ret. Time (min)	Time Offset (min)	Area (counts)	Sep. Code	Width 1/2 (sec)	Status Codes
1		93.859	10.368	0.000	5033913	BB	16.7	
2		6.141	13.707	0.000	329331	BB	30.0	
	Totals:	100.000		0.000	5363244			

Figure S56. Gas chromatogram of benzhydrol (Table 3 Entry 23)

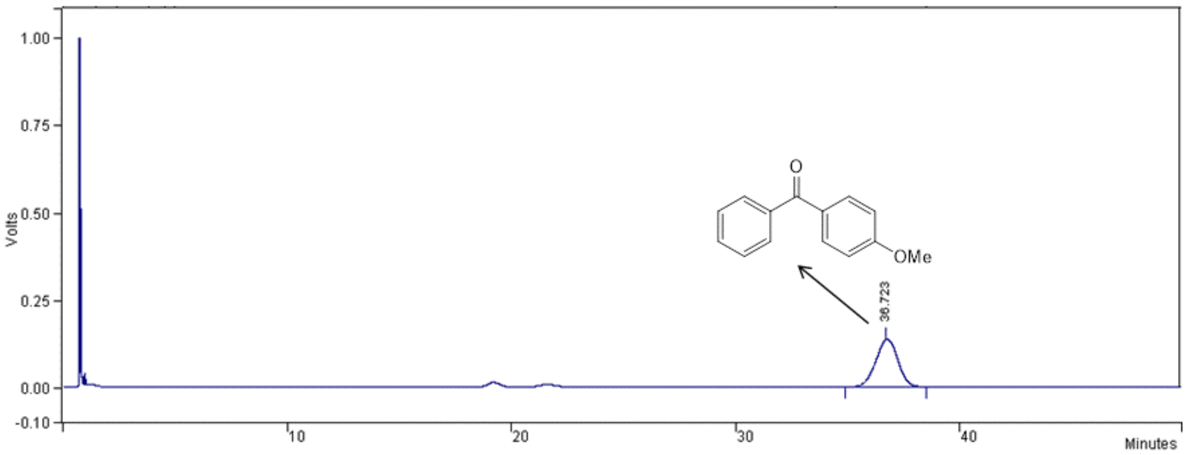

Peak No.	Peak Name	Result ()	Ret. Time (min)	Time Offset (min)	Area (counts)	Sep. Code	Width 1/2 (sec)	Status Codes
1		100.000	36.723	0.000	9274871	BB	62.6	
	Totals:	100.000		0.000	9274871			

Figure S57. Gas chromatogram of 4-methoxybenzhydrol (Table 3 Entry 24)

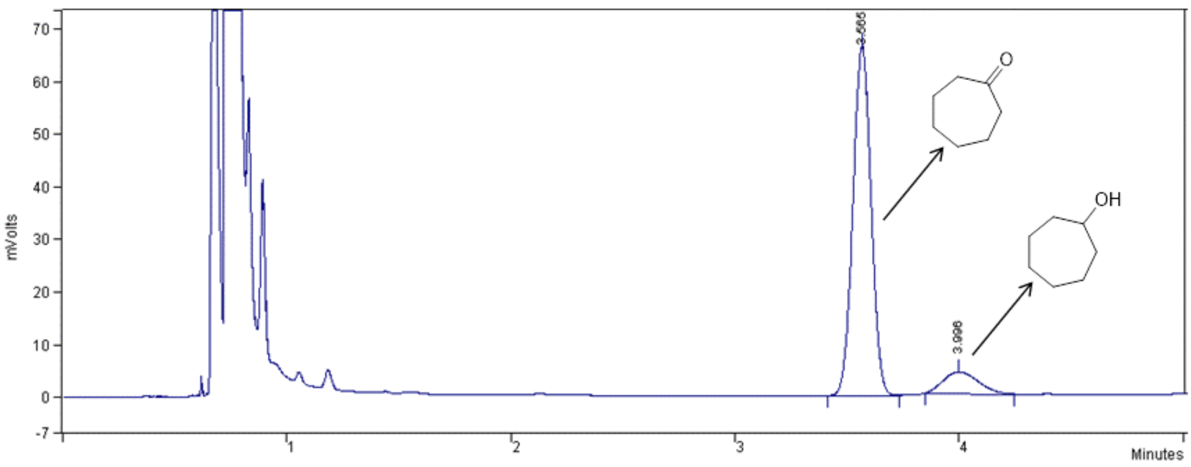

Peak No.	Peak Name	Result ()	Ret. Time (min)	Time Offset (min)	Area (counts)	Sep. Code	Width 1/2 (sec)	Status Codes
1		89.477	3.565	0.000	373849	BB	5.3	
2		10.523	3.996	0.000	43966	BB	10.2	
	Totals:	100.000		0.000	417815			

Figure S58. Gas chromatogram of cycloheptanol (Table 3 Entry 25)

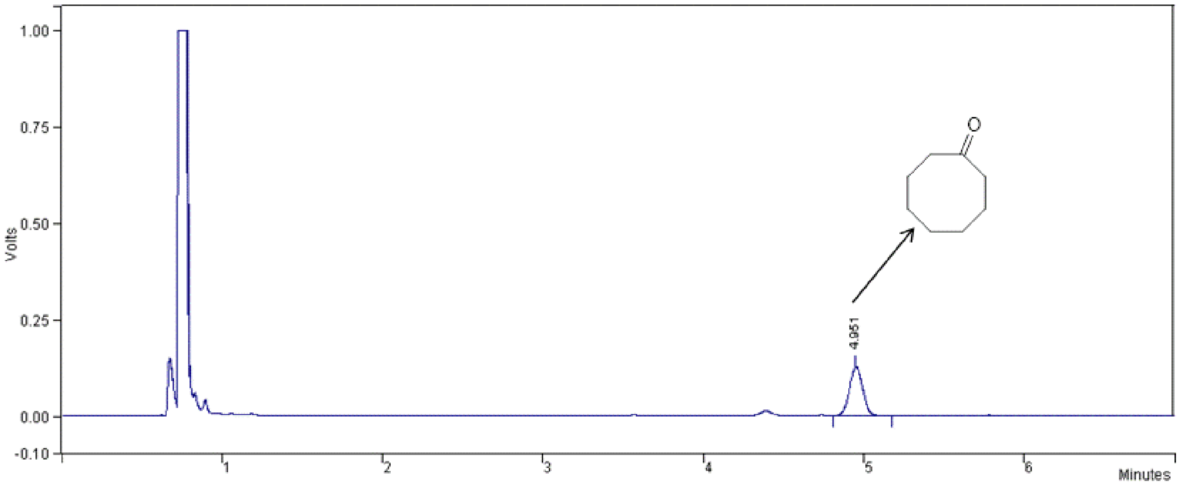

Peak No.	Peak Name	Result ()	Ret. Time (min)	Time Offset (min)	Area (counts)	Sep. Code	Width 1/2 (sec)	Status Codes
1		100.000	4.951	0.000	719869	BB	5.4	
	Totals:	100.000		0.000	719869			

Figure S59. Gas chromatogram of cycloheptanol (Table 3 Entry 26)
